# TumorNext: A comprehensive tumor profiling assay that incorporates high resolution copy number analysis and germline status to improve testing accuracy

**DOI:** 10.18632/oncotarget.11910

**Published:** 2016-09-08

**Authors:** Phillip N. Gray, Huy Vuong, Pei Tsai, Hsaio-Mei Lu, Wenbo Mu, Vickie Hsuan, Jayne Hoo, Swati Shah, Lisa Uyeda, Susanne Fox, Harshil Patel, Mike Janicek, Sandra Brown, Lavinia Dobrea, Lawrence Wagman, Elizabeth Plimack, Ranee Mehra, Erica A. Golemis, Marijo Bilusic, Matthew Zibelman, Aaron Elliott

**Affiliations:** ^1^ Ambry Genetics, Aliso Viejo, CA, 92656, USA; ^2^ Arizona Oncology, Scottsdale, AZ, 85258, USA; ^3^ St. Joseph Health, Orange, CA, 92868, USA; ^4^ Fox Chase Cancer Center, Philadelphia, PA, 19111, USA

**Keywords:** tumor profiling, actionable mutations, next generation sequencing, copy number variants, germline mutations

## Abstract

The development of targeted therapies for both germline and somatic DNA mutations has increased the need for molecular profiling assays to determine the mutational status of specific genes. Moreover, the potential of off-label prescription of targeted therapies favors classifying tumors based on DNA alterations rather than traditional tissue pathology. Here we describe the analytical validation of a custom probe-based NGS tumor panel, TumorNext, which can detect single nucleotide variants, small insertions and deletions in 142 genes that are frequently mutated in somatic and/or germline cancers. TumorNext also detects gene fusions and structural variants, such as tandem duplications and inversions, in 15 frequently disrupted oncogenes and tumor suppressors. The assay uses a matched control and custom bioinformatics pipeline to differentiate between somatic and germline mutations, allowing precise variant classification. We tested 170 previously characterized samples, of which > 95% were formalin-fixed paraffin embedded tissue from 8 different cancer types, and highlight examples where lack of germline status may have led to the inappropriate prescription of therapy. We also describe the validation of the Affymetrix OncoScan platform, an array technology for high resolution copy number variant detection for use in parallel with the NGS panel that can detect single copy amplifications and hemizygous deletions. We analyzed 80 previously characterized formalin-fixed paraffin-embedded specimens and provide examples of hemizygous deletion detection in samples with known pathogenic germline mutations. Thus, the TumorNext combined approach of NGS and OncoScan potentially allows for the identification of the “second hit” in hereditary cancer patients.

## INTRODUCTION

Traditional methods for tumor characterization are tumor-type specific and include assays such as immunohistochemistry (IHC), *in situ* hybridization (ISH), quantitative PCR (qPCR), Sanger sequencing and gene signature microarrays [1–8]. Such assays are highly specific but provide limited information. In contrast, whole genome sequencing (WGS) of tumors using next generation sequencing (NGS) is an unbiased approach that provides extensive genomic information about a tumor. Unfortunately, the cost of sequencing and associated bioinformatics handling of results is still too high for routine clinical WGS of tumor specimens. An alternative approach is exome sequencing, but even this method is not cost effective due to the large amount of data required to detect variants occurring at low frequencies (i.e. at minor allele frequencies ≤ 5%) and time needed to analyze thousands of genes [13]. Targeted gene panels are currently the best option for tumor characterization as they allow multiple genes to be analyzed and can provide enough depth of coverage to detect minor allele frequencies in a cost-effective manner [9–12].

Due to the increasing popularity of NGS-based tumor testing, guidelines for detecting tumor variants have recently been established by the State of New York Health Department [13]. Based on these recommendations, samples should have enough sequence data for a minimum average coverage of 500x so that minor allele frequencies of 5% can be reliably detected. Coverage and its reliability as a quality metric can differ depending on whether amplification-based or probe-based enrichment is utilized [14]. Amplification-based NGS panels are based on the polymerase chain reaction (PCR) and are ideal when analyzing archived tissues for which material is limited. However, unless molecular barcodes are employed, the true depth of coverage cannot be determined with amplicon-based target enrichment, as PCR duplicates cannot be distinguished from amplicons generated from the original template. In addition, allele drop-out (i.e. failed primer binding due to a variant in the primer binding site that results in only one allele being amplified) resulting in false negatives is a potential concern when utilizing primer based enrichment.

In contrast to amplification-based approaches, probe-based enrichment methods use biotinylated oligonucleotide probes of up to 120 nucleotides in length designed to capture a region of interest. Since the probes are much longer than typical PCR primers, variants in the probe binding sites typically do not affect hybridization to the target region, and thus allele drop-out is not an issue. Most probe-based methods require traditional NGS library preparation in which DNA is randomly sheared, so sequence reads for a particular target will have several different start and stop coordinates. As a result, PCR duplicates can be identified and removed from the dataset, which reduces the false positive rate [15] and allows the true sequencing coverage depth to be determined.

The incorporation of a matched blood or normal tissue control from the same patient to differentiate somatic from germline mutations increases the accuracy of NGS-based tumor panels. Jones *et al*. published a comparison of tumor-only vs tumor/germline paired analysis and found up to one-third of actionable mutations in tumor-only analysis are classified incorrectly as somatic when they are actually germline [16]. The misclassification of variants could result in inappropriate treatment recommendations. For example, a tumor may contain a germline variant of unknown significance (VUS) in an oncogene that is predicted to be an activating mutation, but with only an intermediate confidence level. If the VUS was known to be germline, it may instead be classified as benign since inherited mutations in oncogenes are relatively rare [17]. In contrast, if the germline status of the VUS was unknown, it is more likely to be treated as a driver of disease, potentially leading to inappropriate therapy, increased health care costs and lost time for the patient. Thus, knowing the germline status of the variant can not only aid in the clinical management of the patient and their family members but also help correctly classify the variant as likely pathogenic or likely benign. Although the inclusion of incidental germline findings may complicate patient management for the oncologist [18], tumor-only analysis may lead to incorrect variant classification with possible negative impacts on patient safety and healthcare costs.

A number of clinical laboratories offering NGS panels use the data to detect copy number variations (CNVs) in addition to single nucleotide variations (SNVs), insertions and deletions (indels) and structural variants (SVs). CNV analysis of somatic specimens using data from NGS panels is limited, as such analysis cannot detect amplifications below 5× or polyploidy [10], and is restricted to genes on the NGS panel. In contrast, microarray-based platforms can provide whole genome CNV analysis and are highly sensitive, capable of detecting single copy amplifications as well as aneuploidy and polyploidy [19, 20], and have become a standard clinical laboratory test for CNV determination [21–23]. While the number of clinically actionable CNVs is currently limited, it is important to collect whole genome CNV profiles rather than a limited panel as new CNVs involved in oncogenesis may be revealed with data from greater numbers of whole genome profiles. Also, ploidy determination can have clinical significance and serve as a prognostic factor. For example, in prostate cancer, DNA aneuploidy increases with stage and grade and can serve as a prognostic indicator for hormone therapy [24, 25]. Also, aneuploidy of chromosome 17 is a prognostic indictor in breast cancer [26–28]. Although there is an added cost associated with microarray analysis, coupling NGS with a microarray provides the most accurate and reliable genomic profile of a tumor.

Here we describe the analytical validation of a custom probe-based NGS tumor panel, TumorNext, for the detection of SNVs, indels and SVs that uses a matched blood (i.e. non-tumor) control to differentiate between somatic and germline mutations. The panel targets all exons of 142 genes and select introns of 15 genes associated with solid tumors. The genes on the panel are associated with hereditary cancer or known to have clinically actionable mutations, which are genomic alterations that may predict sensitivity or resistance to standard or investigational therapies and include mutations that (*i*) have an FDA approved therapy in the patient's tumor type; (*ii*) have an FDA approved therapy in a different tumor type (off-label); (*iii*) have a drug targeting the altered gene currently in a clinical trial; or (*iv*) provide prognostic information [9, 12]. Also described is the validation of the Affymetrix OncoScan platform, a molecular inversion probe (MIP) array technology for CNV detection in FFPE specimens, intended for use in parallel with the NGS panel.

## RESULTS

### *In silico* testing for bioinformatics pipeline optimization

We conducted an *in silico* analysis using a targeted sequencing dataset comprised of 5,615 target regions covering approximately 3.8 Mb and 413 genes to test the analytical sensitivity and specificity of the TumorNext bioinformatics pipeline. For each type of variant (SNV, deletion and insertion), a total of 32 datasets were simulated by combining 4 different average coverages (100×, 250×, 500× and 1000×) and 8 different heterozygous variant frequency ranges (0 − 3%, 3% − 5%, 5% − 10%, 10% − 20%, 20% − 30%, 30% − 50%, 50% − 80%, 80% − 100%). To test reproducibility, each dataset was simulated three times. For the simulated SNV dataset, simulations were conducted 3 times to generate 650K SNVs total (Supplementary Table S1). The simulation showed that ≥ 500× coverage yielded a sensitivity of 100% and allows detection of alleles down to 3% (Supplementary Table S2). Specificity was also 100% at ≥ 500× coverage (Supplementary Table S3). For the indel simulations, 15 different size lengths were randomly generated and binned: 1 bp, 2 bp, 3 bp, 4 bp, 5 bp, 6 bp, 7 bp, 8 bp, 9 bp, 10 bp, 11−20 bp, 21−30 bp, 31−40 bp, 41−50 bp, > 50 bp (Supplementary Table S4 for deletions and Supplementary Table S7 for insertions). For deletions, ≥ 500× coverage gave a sensitivity of 100% and allowed detection of alleles down to 3% (Supplementary Table S5) and specificity was 100% at all coverage levels (Supplementary Table S6). For insertions, ≥ 500× coverage gave a sensitivity of 100% and allowed detection of alleles down to 3% for most insertion sizes (Supplementary Table S8). Sensitivity began to decline when insertion sizes reach 31bp. Also, insertion detection sensitivity dropped below 100% for 7 bp insertions. Specificity was 100% at all coverage levels (Supplementary Table S9). Based on these results, all somatic samples were sequenced to a minimum average depth of coverage of 500×, which is in line with the New York Health Department guidelines.

### NGS panel

**Figure 1:**
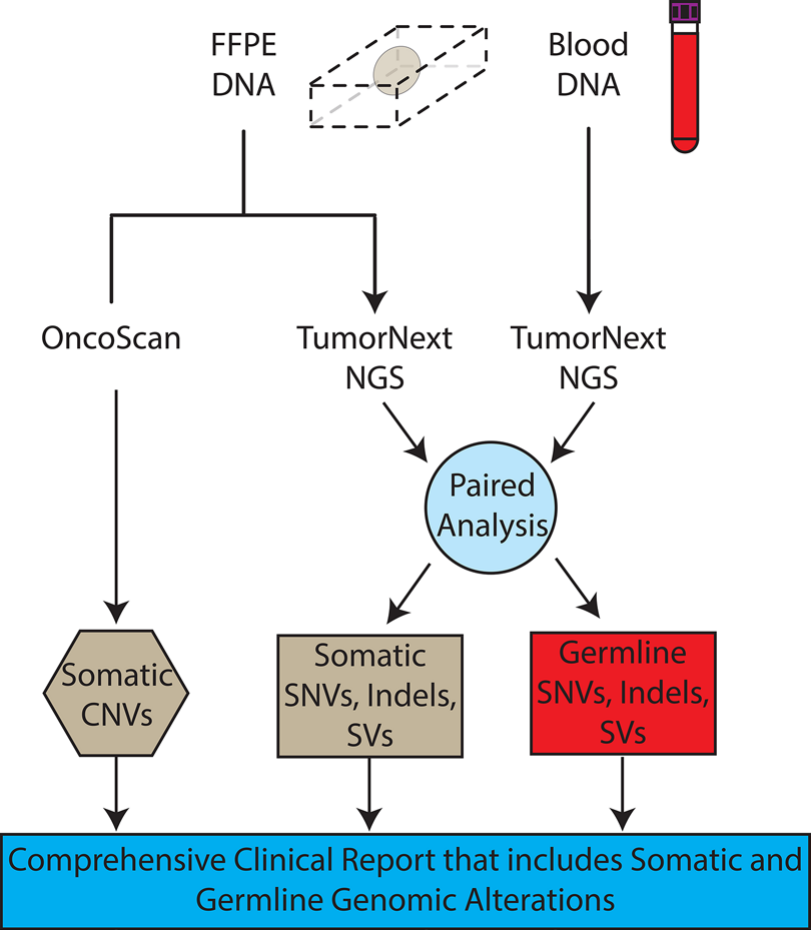
TumorNext workflow DNA is extracted from both FFPE specimens and blood. FFPE DNA is analyzed using OncoScan for CNV detection and TumorNext for SNVs, indels and SVs detection. The TumorNext bioinformatics pipeline uses data from the blood sample to perform a paired analysis, which can differentiate between germline and somatic variants. The TumorNext final report provides a comprehensive analysis of somatic and germline alterations.

A custom panel was designed to analyze 2,350 exons, 153 introns and 4 UTR regions in 142 genes associated with solid tumor cancers (Supplementary Table S10). Approximately 99 genes are frequently mutated in solid tumors and may be targets of FDA-approved or experimental therapies, and/or are prognostic indicators [29]. The remaining 43 genes are associated with hereditary cancers and the mutational status of a subset of these genes may guide treatment (i.e. BRCA1 status and PARP inhibitors). The assay consists of a hybridization-based target enrichment and NGS. DNA is extracted from both FFPE tissue and blood and dual analysis is performed to differentiate between germline and somatic mutations. Deletion and duplication analysis, utilizing whole genome OncoScan microarray technology, is performed concurrently (Figure 1).

### Limit of detection

**Figure 2:**
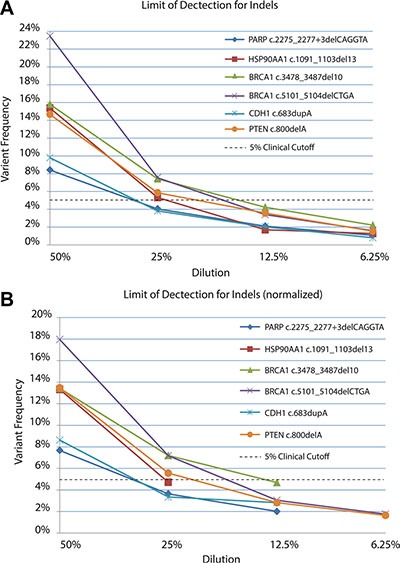
Limit of detection for known indels in non-normalized (A) and normalized (B) datasets DNA extracted from tumors was serial diluted in matched control/blood DNA and samples sequenced to determine the limit of detection.

**Figure 3:**
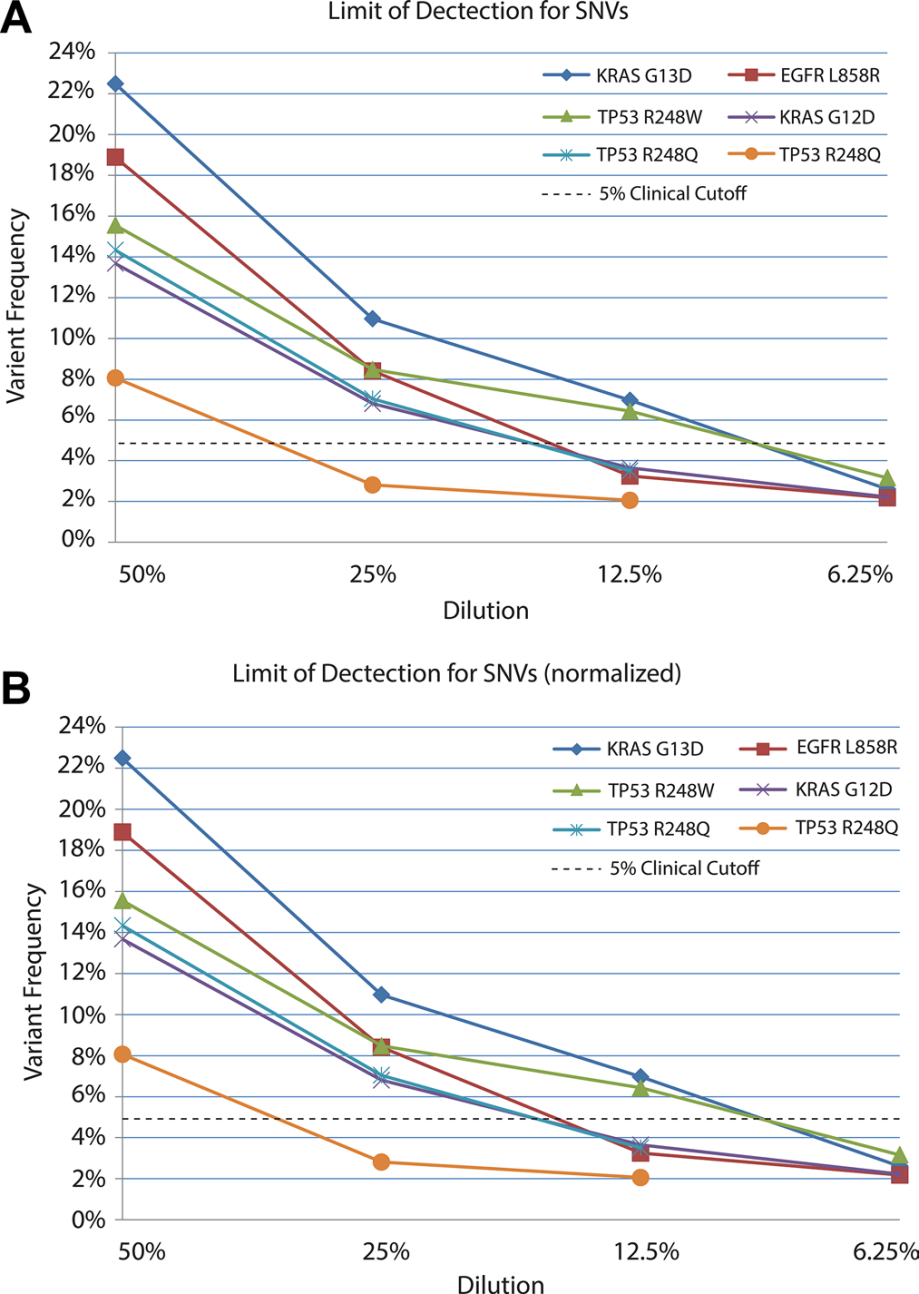
Limit of detection for known SNVs in non-normalized (A) and normalized (B) datasets DNA extracted from tumors was serial diluted in matched control/blood DNA and samples sequenced to determine the limit of detection.

Solid tumor samples are usually a heterogeneous mixture of cells derived from both tumor and normal tissue. Moreover, the tumor cells may consist of several subpopulations of mutant genotypes [30, 31] similar to a viral quasispecies [32]. As a result, allele frequencies within the tumor may occur across a broad range, with mutations of interest present at low levels (i.e. 5%) [33]. *In silico* analysis using simulated datasets indicated our bioinformatics pipeline could detect SNVs and indels down to a minor allele frequency (MAF) of 3% with 500× coverage, as described above. To evaluate the limit of detection of the TumorNext NGS panel, serial dilutions were performed with tumor samples where tumor DNA was diluted with their respective matched normal DNA to generate a range of known heterozygous allele frequencies. Sequencing coverage for indels varied from 390× to 5700×. Analysis for these samples was performed using all sequence data as well as a normalized data set in which reads were removed from samples with high coverage (> 2000×) to yield an average depth of coverage of 1000× for most samples (Table 1). In the non-normalized data set, all variants were detected by the pipeline, including a c.683dupA/p.Y228* mutation in CDH1 that was detected at a MAF of 0.8% in a sample with 3187× coverage (Figure 2A). The detection of variants below 1% was not surprising due to the high depth of coverage for this sample. For the normalized data set, the pipeline was able to detect MAFs below 5% (Figure 2B). Coverage was more uniform for samples with SNVs (Table 2) and similar results were obtained with normalized and non-normalized samples (Figure 3A and 3B) with a MAF of 1.52% detected (with 2436× coverage) in the non-normalized data set, and 2.06% detected (with 1066× coverage) in the normalized data set. Similar to what others have reported [34], the TumorNext analysis pipeline is limited by specificity rather than sensitivity. Therefore, our assay can detect variants with allele frequencies of 5% or higher with 100% concordance.

**Table 1:** Limit of detection study: normalized coverage for samples containing known indels

Sample	Variant Type	Gene Symbol	Annotated Variant based on HGVS	1:2 Coverage	1:4 Coverage	1:8 Coverage	1:16 Coverage
RD_008	deletion	PARP1	c.2275_2277+3delCAGGTA	964	935	946	914
	deletion	HSP90AA1	c.1091_1103del13 p.Y364Ffs*10	390	594	483	656
RD_009	deletion	BRCA1	c.3478_3487del10 p.K1160Lfs*47	1489	1527	1534	1509
RD_015	deletion	BRCA1	c.5101_5104delCTGA p.L1701Nfs*4	1119	1130	1086	1136
BR13_102	insertion	CDH1	c.683dupA p.Y228*	928	956	955	962
BR12_110	deletion	PTEN	c.800delA p.K267Rfs*9	905	846	922	977

**Table 2:** Limit of detection study: normalized coverage for samples containing known SNVs

Sample	Variant Type	Gene Symbol	Annotated Variant based on HGVS	1:2 Coverage	1:4 Coverage	1:8 Coverage	1:16 Coverage
BR13_184	SNV	KRAS	c.38G > A p.G13D	1023	1077	1106	1072
BR14_75	SNV	EGFR	c.2573T > G p.L858R	1038	1047	1109	1053
BR13_104	SNV	TP53	c.742C > T p.R248W	997	1003	1057	1045
BR12_110	SNV	KRAS	c.35G > A p.G12D	1061	1132	1069	1127
BR13_10	SNV	TP53	c.743G > A p.R248Q	1055	966	1029	1023
BR14_67	SNV	TP53	c.743G > A p.R248Q	955	960	1066	1019

### Analytical sensitivity

Four sets of samples were used to determine analytical sensitivity: HapMap samples with known genotypes (2 samples), FFPE tumor specimens previously characterized using the OncoScan hotspot panel (111 samples), tumor specimens previously characterized on CytoSNP850K (35 samples) and tumor specimens previously characterized by Foundation Medicine, Guardant Health or Caris (16 samples). A summary of all mutations analyzed from FFPE specimens is listed in Table 3. First, we analyzed two HapMap samples (NA10857 and NA07019) and found 91/94 calls were concordant with the reference sequence with 3 discordant calls in NA070019 (rs6685892, rs3899528 and rs619203). These 3 SNPs were Sanger sequenced and found to be concordant with the NGS data; indicating the reference genotype for these SNPs was incorrect (Supplementary Figure S1). Overall, concordance was 100% (94/94 calls) when comparing TumorNext NGS data to the 2 HapMap sample genotypes (Supplementary Tables S11 and S12).

**Table 3:** FFPE validation samples with previously known mutations

Mutation Class	Gene	Mutation	Mutations Recovered
SNV	AKT2	p.W22*	1/1
SNV	APC	p.L1129S	1/1
SNV	ATM	p.V2424G	1/1
SNV	AURKB	p.R248H	1/1
SNV	BRAF	p.V600E	3/3
SNV	BRCA2	p.F233V	1/1
SNV	EGFR	p.L858R	1/1
SNV	EPHA5	p.P141T	1/1
SNV	EPHB1	p.F699Y	1/1
SNV	ERBB2	p.R896C	1/1
SNV	ERBB2	c.575-3C > T	1/1
SNV	ESR1	p.A571V	1/1
SNV	FANCD2	p.P593S	1/1
SNV	FGFR1	p.S393L	1/1
SNV	FGFR1	p.R822C	1/1
SNV	FLT3	p.V197L	1/1
SNV	FLT4	p.T810K	1/1
SNV	JAK3	p.V628D	1/1
SNV	KRAS	p.G12D	9/9
SNV	KRAS	p.G12V	4/4
SNV	KRAS	p.G12C	1/1
SNV	KRAS	p.G12S	1/1
SNV	KRAS	p.G13D	1/1
SNV	KRAS	p.Q61K	1/1
SNV	KRAS	p.Q61H	1/1
SNV	MLL	p.A3247A	1/1
SNV	MYD88	p.V188L	1/1
SNV	NF1	p.W2494*	1/1
SNV	NF2	p.R198*	1/1
SNV	NOTCH1	p.G1342S	1/1
SNV	NRAS	p.G12D	1/1
SNV	NRAS	p.Q61R	1/1
SNV	PDGFRB	p.V316M	1/1
SNV	PIK3CA	p.E545K	3/3
SNV	PIK3CA	p.E542K	1/1
SNV	PIK3CA	p.H1047R	4/4
SNV	PIK3CG	p.R359H	1/1
SNV	RET	p.R77H	1/1
SNV	ROS1	p.R2116I	1/1
SNV	TP53	p.R282W	1/1
SNV	TP53	p.R306*	1/1
SNV	TP53	p.R213*	3/3
SNV	TP53	p.R175H	2/2
SNV	TP53	p.R248Q	5/5
SNV	TP53	p.K132N	2/2
SNV	TP53	p.S215G	1/1
SNV	TP53	c.782 + 1G > A	1/1
SNV	TP53	p.R273H	3/3
SNV	TP53	p.R273C	2/2
SNV	TP53	p.G245V	1/1
SNV	TP53	c.673-1G > C	1/1
SNV	TP53	p.G266V	1/1
SNV	TP53	p.C275F	1/1
SNV	TP53	p.Y220C	1/1
SNV	TSC2	p.A196T	1/1
SNV	VHL	p.R3L	1/1
Indel	BRCA1	p.S1655fs*16	2/2
Indel	BRCA1	p.K1160Lfs*47	1/1
Indel	BRCA1	p.L1701Nfs*4	1/1
Indel	HSP90AA1	p.Y364Ffs*10	1/1
Indel	PARP1	c.2275_2277 + 3delCAGGTA	1/1
Indel	PTEN	p.K267fs*9	1/1
SV	BRCA1	ins6kbEx13 duplication	1/1

The OncoScan array was designed primarily to detect CNVs, but it also contains a small hot spot panel that can detect a total of 74 clinically actionable somatic mutations in 9 genes, which includes both SNVs and indels (Supplementary Table S13). A total of 111 samples were run on OncoScan and later analyzed on TumorNext (Supplementary Table S14). TumorNext detected all mutations detected by the OncoScan hotspot panel, but one mutation was inappropriately filtered out. The mutation was present in 22.66% of tumor sequence reads and 13.39% of control reads, so it was classified at a germline variant and filtered out of the somatic data. The call was PIK3CA p.H1047R/c.3140A > G, which is a well-known oncogene mutation [35] and highly unlikely to be a germline mutation. Notably, the matched control for this specimen was tissue, whereas most other control DNA was derived from blood, and contained 15% tumor. As a result, the PIK3CA H1047R mutation was filtered out because it was present in both the tumor and matched control. While 15% tumor is high, this sample was provided to us as a non-tumor matched control and the possibility of this occurring with matched “normal” tissue with less tumor contamination (i.e. < 10%) is a concern. As a result, our pipeline was modified to retain all calls when using tissue as a matched control. When taking all samples (111) and mutations screened (74) into account, the total number of regions analyzed was 8214, and overall concordance was 100% (8214/8214).

The Illumina CytoSNP850K bead chip array contains ∼850,000 SNPs spanning the entire genome with enriched coverage for 3,262 genes frequently containing copy number alterations in somatic cancer cells. The number of SNPs covered by both TumorNext and CytoSNP850K was determined to be 2,035. A total of 35 FFPE samples that were previously run on CytoSNP850K with a SNP confidence score > 95 (n.b., Illumina recommends > 99% for non-FFPE samples) were used to determine accuracy of TumorNext. Although 138 out of the 2,035 SNPs were discordant, this reflected poor calls for only 27 SNPs in 19 genes (Supplementary Tables S15 and S16), suggesting these SNPs do not perform well on the CytoSNP850K array with FFPE samples. All discordant calls were Sanger sequenced and 134 of 135 were determined to be false positives on the CytoSNP850K array. The one discordant call was detected by TumorNext, but filtered out due to low coverage. The sample (SP11_180A14) had a depth of coverage of 587×, but coverage for the discordant SNP was only 67× (below the 100× threshold for variant calling), causing it to be filtered out. This particular SNP (EPHA2 rs2230597) showed up as a false positive in 3 other samples (i.e. Sanger sequencing did not confirm the call) and is located in a G/C rich region. A total of 223 samples (including samples run multiple times) were run for the validation and the average coverage was over 1100×, so this sample had below average coverage. The overall concordance was 99.94% (1,979/1,980) between TumorNext and CytoSNP850K array.

The third measure of accuracy consisted of comparing samples previously analyzed by Caris, Guardant Health or Foundation Medicine to results from TumorNext. A total of 29 variants (mutations and variants of unknown significance) were reported by Foundation Medicine and Guardant Health in targets covered by TumorNext. TumorNext detected 29 of 29 variants, in addition to 1 intronic variant outside Foundation's reporting range, which was Sanger confirmed. A total of 7 variants were reported by Caris in targets covered by TumorNext. TumorNext detected 7 of 7 variants in addition to 14 variants, which were all Sanger confirmed. Seven of these variants were on the Caris NGS panel, but not listed in their report (Table 4). Combined, a total of 51 variants were detected by TumorNext for this sample set and all concordant with either: *i)* results reported by the other labs, *ii)* Sanger confirmed variants, *iii)* results observed on the OncoScan hotspot panel or *iv)* results observed from multiple assays (i.e. Sanger and OncoScan).

**Table 4:** Concordance between TumorNext and Foundation Medicine, Guardant Health and Caris clinical reports

Sample	TumorNext Results	Caris, Foundation Medicine or Guardant Health
RD_001_C1	ERBB2 R896C - germline	ERBB2 R896C (F)
	BRCA1 S1655fs*16 - germline	BRCA1 S1655fs*16 (F)
	TP53 K132N	TP53 K132N (F)
	ESR1 A571V – germline	ESR1 A571V – VUS (F)
RD_001_G1	BRCA1 S1655Yfs*16 – germline (SC)	BRCA1 S1655fs*16 (C)
	TP53 K132N (SC)	TP53 K132N (C)
RD_003	TP53 S215G (SC)	TP53 S215G (F)
	FLT4 T810K (SC) - germline	FLT4 T810K – VUS (F)
RD_004	TP53 c.782 + 1G > A (SC)	No somatic mutations detected (C)
RD_005	NRAS G12D (OS) (SC)	NRAS G12D (F)
	PIK3CA E545K (OS) (SC)	PIK3CA E545K (F)
	FANCD2 P593S - germline	FANCD2 P593S – VUS (F)
	PIK3CG R359H - germline	PIK3CG R359H – VUS (F)
RD_007	EPHB1 F699Y (SC) - germline	EPHB1 F699Y – VUS (F)
	BRCA2 F233V (SC) - germline	BRCA2 F233V – VUS (F)
	TP53 R273H (SC)	TP53 R273H (F)
	AURKB R248H – germline	AURKB R248H – VUS (F)
	FGFR1 S393L, R822C – germline	FGFR1 S393L, R822C – VUS (F)
	PDGFRB V316M - germline	PDGFRB V316M – VUS (F)
RD_008	^**^NF1 W2494* (SC)	No somatic mutations detected (C)
	^**^PARP1 c.2275_2277+3delCAGGTA (SC)	
	^**^HSP90AA1 Y364Ffs*10 (SC)	
RD_009	TP53 R282W (SC) (OS)	TP53 R282W (F)
	ERBB2 c.575-3C > T (SC)	
	NF2 R198* (SC)	NF2 R198* (F)
	BRCA1 K1160Lfs*47 (SC)	BRCA1 K1160Lfs*47 (F)
	NOTCH1 G1342S - germline	NOTCH1 G1342S - VUS (F)
RD_010	^**^MLL A3247A (SC)	
	TP53 G245V (SC)	TP53 G245V (C)
	APC L1129S - germline	APC L1129S - VUS (C)
RD_012	^**^MYD88 V188L (SC)	No somatic mutations detected (C)
	^**^EPHA5 P141T (SC)	
	FLT3 V197L (SC)	
	TP53 c.673-1G > C (SC)	
RD_013	RET R77H (SC)	No somatic mutations detected (C)
RD_014	TP53 G266V (SC)	TP53 G266V (G)
RD_015	TP53 C275F (SC)	TP53 C275F (C)
	BRCA1 L1701Nfs*4 (SC)	BRCA1 L1701fs (C)
	VHL R3L (SC)	
	JAK3 V628D (SC)	
	^**^ROS1 R2116I (SC)	
RD_016	TP53 Y220C (OS)	TP53 Y220C (F)
	AKT2 W22* - germline	AKT2 W22* – VUS (F)
	TSC2 A196T - germline	TSC2 A196T – VUS (F)
RD_017	ATM V2424G - germline	ATM V2424G – VUS (F)
RD_018	KIT p.S123F - germline	KIT S123F (F)
	BRCA2 K3326* - germline	BRCA2 K3326* - VUS (F)
	TSC1 H732Y - germline	TSC1 H732Y - VUS (F)
	TP53 M237I	TP53 M237I (F)
	BRAF D594N	BRAF D594N (F)

** Gene not on Caris NGS panel

Legend: (C) = Caris, (F) = Foundation Medicine, (G) = Guardant Health, (OS) = detected on the OncoScan Hotspot panel, (SC) = Sanger confirmed, VUS = Listed as variant of unknown significance on the Foundation One report.

In total, 2303 genotypes determined by other platforms (including novel findings that were Sanger confirmed) were compared to TumorNext and 0 false positives were detected and 1 false negative was observed. Based on these results, the analytical sensitivity (True Positives/True Positives + False Negatives) of the TumorNext panel was 2302/2302 + 1 = 99.96% (95% CI, 99.76%-100%).

We determined some variants listed on the Foundation Medicine reports to be germline. Most of these were listed at VUSs, however, two were listed as actionable and therapy recommended. Sample RD_001_C1 had a germline mutation in ERBB2 (R896C), which was reported as actionable and treatment recommended. This mutation does have a COSMIC mutation Id (COSM14066) and reported as a HER2 activating mutation [36], so it is understandable why it was included in the report. However, germline mutations in oncogenes are rare, so it is highly unlikely this mutation is the driver of disease. The same observation was made for Sample RD_018, which had a germline mutation in the oncogene KIT (S123F), which also has a COSMIC mutation ID (COSM317523). Again, Foundation Medicine recommended treatment based on this mutation, but as discussed, this variant is unlikely to be a driver of disease. These examples highlight the value of determining germline status of variants for accurate classification/assessment of contribution to disease when performing tumor profiling.

**Figure 4:**
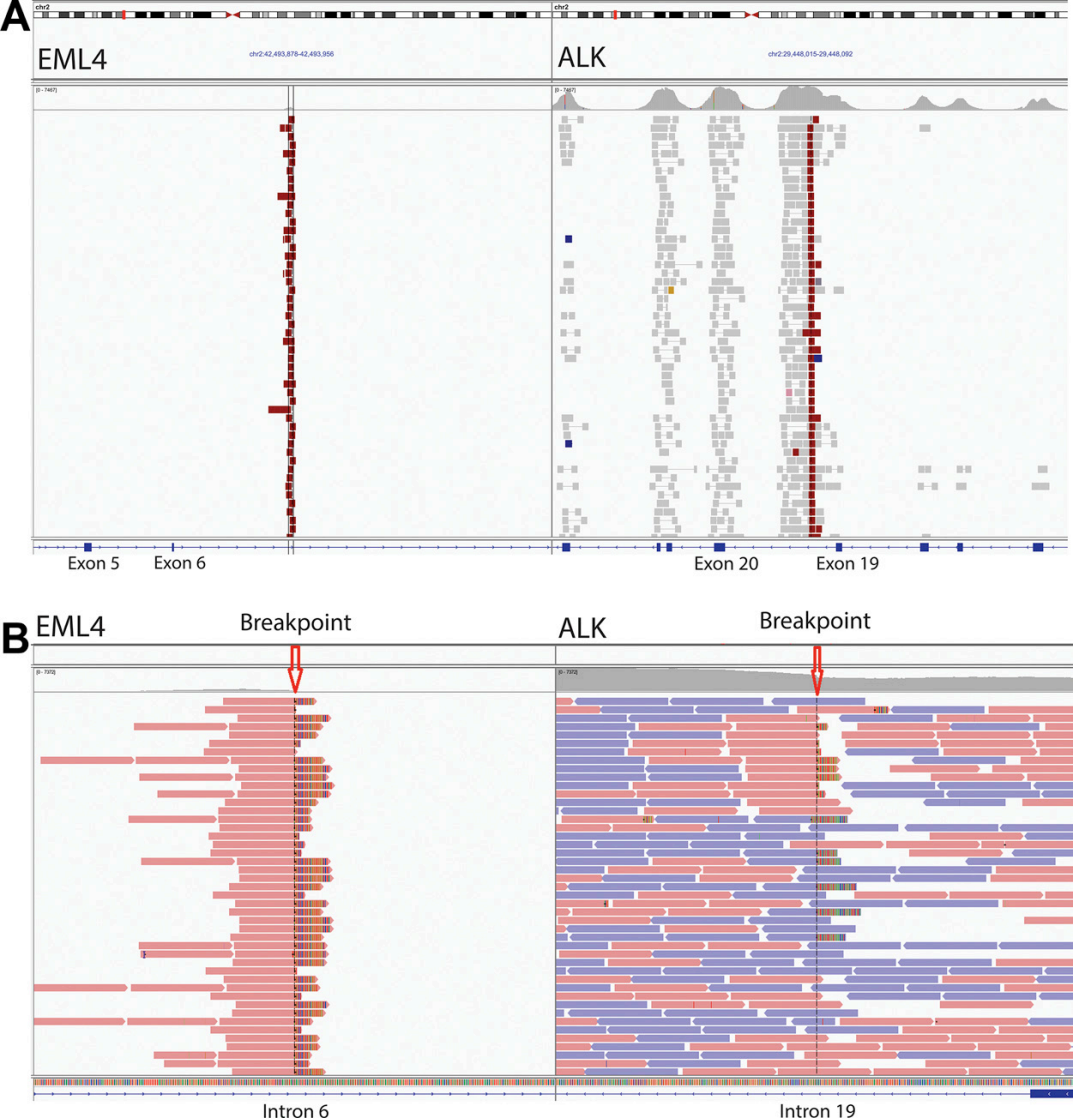
Gene fusion detection by TumorNext (**A**) IGV screenshot of the EML4-ALK inversion showing high coverage of the breakpoint and surrounding exons in ALK. (**B**) Zoomed in screenshot showing the breakpoints and several split reads that support the fusion (Note: the multi-colored part of the read aligns to the fusion partner).

**Figure 5:**
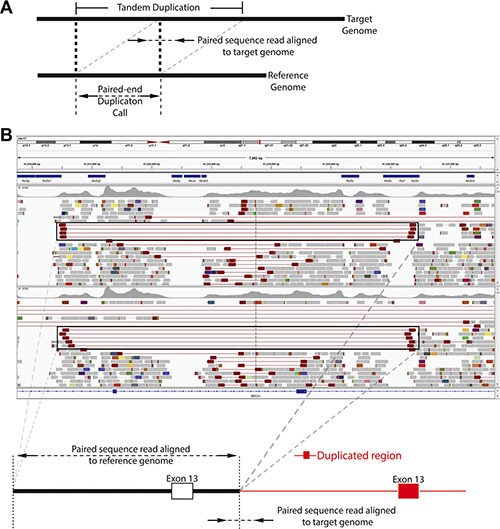
Tandem duplication detection by TumorNext (**A**) Schematic of tandem duplication detection using paired end reads. Reads are represented by arrows. Standard paired end reads face each other as represented in the target genome alignment. A tandem duplication will result in paired end reads facing away from each other when aligned to a reference genome. (**B**) IGV screenshot of two tumor specimens with a known BRCA1 exon-13 6 kb duplication and schematic of the duplication. The supporting paired end reads are located inside the boxes.

The TumorNext panel was designed to detect gene fusions and structural variants by capturing both introns and exons in 15 genes. For some genes, such as ALK, only one intron was captured as the characteristic breakpoint for the oncogenic fusion of interest (EML4-ALK) is well defined. For other genes, all introns were captured as the breakpoints for fusions of interest are unknown. For validation, the cell line H2228 containing the EML4-ALK fusion and a clinical specimen containing the BRCA1 exon-13 6kb duplication (Supplementary Figure S2) were used to demonstrate the panel's ability to detect fusions and structural variants. Figure 4 shows Integrative Genomics Viewer (IGV) screenshots of the EML4-ALK fusion. A total of 102 paired end reads and 118 split reads supported the inversion event in the H2228 cell line. Figure 5 shows IGV screenshots of the BRCA1 exon-13 6kb duplication from two different tumor blocks from the same patient, and two separate sequencing runs. Only paired end reads were detected in support of this tandem duplication, which numbered 4 or 5 per run. The breakpoint for this tandem duplication is located in a low complexity region containing several Alu-repeat elements, so the capture efficiency for this region is low (compatible with the low number of supporting reads).

### Analytical specificity

Two of approaches were used to determine analytical specificity. First, two HapMap reference and sixteen characterized samples (from Foundation, Guardant Health or Caris) were sequenced and 0 false positive calls were made from the 411,115 bp panel (or 7,400,070 total base pairs for 18 samples). Fourteen new variants were detected in the Caris samples, and all were Sanger confirmed. These data indicate the analytical specificity is 100%, as none were false positives. Second, data from 8214 sites genotyped by OncoScan (111 samples × 74 mutations analyzed) were concordant with the TumorNext NGS data. Of these sites, 8164 were true negatives and there were no false positives, so the analytical specificity (True Negatives/True Negatives + False Positives) is 8164/8164 + 0 = 100% (95% CI, 99.95%−100%).

### Precision and reproducibility

**Figure 6:**
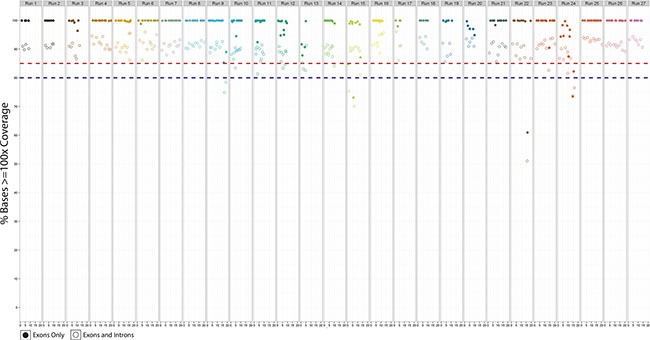
The percent bases covered for target exons only (solid circles) and target exons and introns (hollow circles) for all 27 validation runs.

Seven samples with Sanger-confirmed somatic mutations were used to assess intra- and inter-reproducibility. Mutations included point mutations, deletions and a gene fusion. Samples were assayed in triplicate as intra- (1 sample) or inter-run replicates (6 samples) and were prepared separately by different technicians on multiple dates using non-redundant barcodes to minimize potential barcode bias. All known variants were detected in each run, with the majority detected at similar frequencies and normalized coverage across replicates (Table 5). The reproducibility of coverage was also assessed by determining the percent of bases detected ≥ 100× across all 27 validation runs for exons only, and for the total capture region (Figure 6). As expected, the percentage of bases with ≥ 100× coverage was higher for exons, as exon targets typically are higher complexity sequences and are not typically associated with increased off-target sequence (which reduces overall target specificity and coverage). In contrast, introns are comprised mostly of low complexity sequences and are associated with higher off-target sequence when enriched with probe-based capture methods.

**Table 5:** Results of reproducibility study for TumorNext

Sample	Known Variant	Run	Barcode	Sample Coverage	Variant Called?	Variant Coverage	Variant Frequency	Normalized Coverage
RD_006	KRAS p.G12D	1	B03	2187	Yes	2615	35.45%	1.20
	NM_033360	2	B01	1492	Yes	1474	28.85%	0.99
	c.35G>A	3	B09	2622	Yes	3122	31.62%	1.19
	NF1 p.A132G	1	B03	2187	Yes	2391	33.71%	1.09
	NM_001042492	2	B01	1492	Yes	1506	29.97%	1.01
	c.395C>G	3	B09	2622	Yes	2787	32.05%	1.06
	BRAF p.G464V	1	B03	2187	Yes	2297	35.50%	1.05
	NM_004333	2	B01	1492	Yes	1799	30.91%	1.21
	c.1391G>T	3	B09	2622	Yes	3217	35%	1.23
RD_007	BRCA2 p.F233V	1	B04	742	Yes	815	34.97%	1.10
	NM_000059	2	B02	294	Yes	345	30.72%	1.17
	c.697T>G	3	B10	1420	Yes	1732	33.25%	1.22
	TP53 p.R273H	1	B04	742	Yes	461	68.11%	0.62
	NM_000546	2	B02	294	Yes	291	69.66%	0.99
	c.818G>A	3	B10	1420	Yes	1186	70.35%	0.84
	EPHB1 p.F699Y	1	B04	742	Yes	1018	23.99%	1.37
	NM_004441	2	B02	294	Yes	465	24.24%	1.58
	c.2096T>A	3	B10	1420	Yes	1970	22.19%	1.39
RD_008	PARP1	1	B08	1710	Yes	1725	35.50%	1.01
	NM_001618 c.2275_2277+3delCAGGTA	2	B03	397	Yes	416	27.16%	1.05
		3	B11	917	Yes	884	30.67%	0.96
	HSP90AA1 p.Y364Ffs^*^10 NM_005348 c.1091_1103del13	1	B08	1710	Yes	1913	29.41%	1.12
		2	B03	397	Yes	242	16.12%	0.61
		3	B11	917	Yes	906	20.94%	0.99
	NF1 p.W2494^*^	1	B08	1710	Yes	870	65.44%	0.51
	NM_001042492	2	B03	397	Yes	143	52.82%	0.36
	c.7482G>A	3	B11	917	Yes	436	59.95%	0.48
RD_009	ERBB2	1	B09	1423	Yes	888	50.96%	0.62
	NM_004448	2	B04	1162	Yes	623	48.48%	0.54
	c.575-3C>T	3	B19	1989	Yes	1232	44.32%	0.62
	BRCA1 p.K1160Lfs^*^47 NM_007294 c.3478_3487del10	1	B09	1423	Yes	1567	41.86%	1.10
		2	B04	1162	Yes	1423	38.89%	1.22
		3	B19	1989	Yes	2363	39.62%	1.19
	TP53 p.R282W	1	B09	1423	Yes	713	56.68%	0.50
	NM_000546	2	B04	1162	Yes	829	48.55%	0.71
	c.844C>T	3	B19	1989	Yes	1217	49.12%	0.61
	NF2 p.R198^*^	1	B09	1423	Yes	965	48.70%	0.68
	NM_000268	2	B04	1162	Yes	753	38.18%	0.65
	c.592C>T	3	B19	1989	Yes	1334	44.83%	0.67
RD_010	MLL p.A3247A	1	B10	1035	Yes	1308	85.87%	1.26
	NM_001197104	2	B08	504	Yes	544	83.43%	1.08
	c.9741C>T	3	B20	695	Yes	788	86.83%	1.13
	TP53 p.G245V	1	B10	1035	Yes	554	82.31%	0.54
	NM_000546	2	B08	504	Yes	383	85.04%	0.76
	c.734G>T	3	B20	695	Yes	370	86.62%	0.53
H2228	EML4 (intron 6)-ALK fusion (intron 19) chr2:29448092>chr2:42493956	1	B21	5428	Yes	934	^*^N/A	0.17
		2	B02	4129	Yes	756	^*^N/A	0.18
		3	B19	5672	Yes	1051	^*^N/A	0.19
RD_005	NRAS p.G12D	4	B20,B22,B25	1317	Yes	1399	89.34%	1.06
	NM_002524	4	B20,B22,B25	1783	Yes	1975	88.29%	1.11
	c.35G>A	4	B20,B22,B25	1141	Yes	1297	86.31%	1.14
	PIK3CA p.E545K	4	B20,B22,B25	1317	Yes	1035	32.21%	0.79
	NM_006218	4	B20,B22,B25	1783	Yes	1232	31.11%	0.69
	c.1633G>A	4	B20,B22,B25	1141	Yes	921	30.84%	0.81

* Frequency cannot be determined for structural variants using DELLY

### OncoScan to detect gross abnormalities

The Affymetrix OncoScan system is a microarray-based platform that utilizes molecular inversion probes (MIPs), a target enrichment technology that functions via capture by circularization [37]. This process works by designing a single stranded DNA probe that has complementary regions to a genomic target at its 5′ and 3′ends. The complementary regions hybridize to the target leaving a single nucleotide gap (typically a SNP) between the ends that is subsequently filled in, resulting in a circularized probe. Non-reactive probes and genomic DNA are removed by exonuclease treatment. The circularized probes are processed further and hybridized to the OncoScan array for CNV analysis. The main advantage of this technology is the fact the sample DNA is only used for the initial MIP hybridization. All subsequent steps use the MIPs that successfully bound to their target region. As a result, the assay is not as sensitive to DNA quality as other methods, making it ideal for FFPE specimens. The platform is very robust and its utility has been demonstrated in over 140 publications.

### Analytical sensitivity

The analytical sensitivity of OncoScan was determined using FFPE specimens previously characterized by ISH, IHC, qPCR, CytoSNP850K and NGS. First, a total of 30 breast and 9 ovarian cancer specimens with known HER2/*neu* status were analyzed on OncoScan (Table 6). The HER2/*neu* status was determined by either FISH (23 samples; 3 equivocal), IHC (7 samples), Oncotype DX (2 samples), a qPCR assay offered by Genomic Health (Redwood City, CA), or CISH (7 samples). All IHC and Oncotype DX results were negative (i.e. not amplified) and matched the OncoScan data. There were some discordant calls between the ISH data and OncoScan; however, some of the discrepancies may be due to the limitations inherent in ISH assays. FISH and CISH scoring is calculated by comparing the ratio of HER2 (ERBB2) probe signals to probes targeting the centromeric region of chromosome 17 (CEP17), as well as the average HER2 probe signals per cell. Per ASCO guidelines for duel probe analysis [38], ratios < 2.0 with an average HER2 copy number < 4.0 signals per cell indicate a non-amplification. Ratios < 2.0 with an average HER2 ≥ 4.0 and < 6.0 are equivocal. To classify as HER2-positive, three scenarios are possible for dual probes: 1) ratios ≥ 2.0 with an average HER2 copy number ≥ 4.0, 2) ratios ≥ 2.0 with an average HER2 copy number < 4.0 and 3) ratios < 2.0 with an average HER2 copy number ≥ 6.0 [38]. It is well known the HER2/CEP17 ratio can be skewed if the copy number of the CEP17 region is altered [39, 40]. For example, if CEP17 is co-amplified with HER, no difference will be observed in the HER2/CEP17 ratio.

**Table 6:** HER2/*neu* status: comparison of oncoscan to previously characterized samples

Specimen ID	HER2/*neu* Status	Average HER2 spot count	OncoScan Result
	FISH Result (ratio)		
H2N1	Unamplified (1.2)	2.6	2n
H2N2	Unamplified (0.7)	1.9	2n
H2N3	Unamplified (1.3)	2.4	2n
H2N4	Unamplified (0.9)	1.7	1.67n^**^
H2N5	Unamplified (1.2)	3.1	2n
H2N6	Unamplified (1.2)	2.3	2n
H2N7	Unamplified (1.2)	2	2n
H2N8	Equivocal (1.5)	4.4	2n
H2N9	Amplified (2.4)	6.5	4n
H2N10	Amplified (2.0)	4.3	3n
H2N11	Amplified (1.2)	6	2.33n^**^
H2N12	Unamplified (1.1)	1.6	2n
H2N13	Unamplified (1.1)	3	3n^*^
H2N14	Unamplified (1.1)	3.1	4n^*^
H2N15	Unamplified (1.1)	2.9	2n
H2N16	Unamplified (1.1)	3	2n
H2N17	Equivocal (1.4)	4.1	2n
H2N18	Amplified (2.4)	5	4n
H2N19	Unamplified (1.1)	3.5	2n
H2N20	Unamplified (1.6)	3.4	4n^*^
H2N21	Unamplified (1.1)	3.3	4n^*^
H2N22	Amplified (2.1)	3.3	6n
H2N23	Equivocal (1.9)	4.9	4n^*^
	**CISH Results (ratio)**		
H2N24	Unamplified (0.94)	1.8	2n
H2N25	Unamplified (1.2)	2.1	2n
H2N26	Unamplified (1.03)	1.85	1n
H2N27	Unamplified (1.09)	1.8	2n
H2N28	Unamplified (1.1)	2.2	2n
H2N29	Unamplified (1.03)	1.95	3n
H2N30	Unamplified (1.16)	1.85	2n
	**IHC Results**		
H2N31	Negative	N/A	2n
H2N32	Negative	N/A	2n
H2N33	Negative	N/A	2n
H2N34	Negative	N/A	2n
H2N35	Negative	N/A	2n
H2N36	Negative	N/A	2n
H2N37	Negative	N/A	2n
	**Oncotype DX Results**		
H2N38	Negative	N/A	2n
H2N39	Negative	N/A	1n

* Sample has CEP17 amplification.

** Note: The copy number reported by OncoScan is the average copy number for aberrant/tumor cells only. If the TuScan software cannot determine % aberrant cells/tumor, the copy number reported will be from the total cell population and will be a non-integer (i.e. 1.67, 2.33, etc.).

A total of 30 specimens with known HER2/*neu* status determined by FISH or CISH were analyzed on OncoScan. Three of these specimens (H2N8, H2N17 and H2N23) were classified as equivocal due to an average HER2 spot count of ≥ 4.0 and < 6.0 and HER2/CEP17 ratio of < 2.0. OncoScan determined 2 of these specimens to be unamplified (CN = 2) and 1 amplified (CN = 4). The amplified specimen contained a CEP17 amplification, which will skew the HER2/CEP17 ratio. With the HER2 spot count at 4.9, this sample would easily be classified as Her2+ with a corrected HER2/CEP17 ratio. The other 2 samples did not contain CEP17 amplifications, and their HER2/CEP17 ratios match the reported OncoScan copy number (CN = 2). The average HER2 spot count was determined to be greater than 4 for both samples, which highlights a drawback from using ISH for determining HER2/*neu* status. ISH analysis only evaluates a limited number of cells (40 cells) and HER2 status may vary in different regions of the analyzed tumor section and throughout the specimen due to heterogeneity. In contrast, OncoScan analyzes DNA from 80 ng of DNA, which equates to ∼13K diploid cells (1ng of DNA contains DNA from 167 diploid cells). Moreover, ISH analysis can be biased as a pathologist may focus on an area with high staining, whereas OncoScan provides data from the entire specimen. Sample H2N11 also highlights this issue as it was classified as HER2/*neu* amplified solely from the average HER2 spot count. OncoScan determined this sample to be highly heterogeneous with HER2 amplified (CN = 2.33) in only a minority of cells. This was a case where the HER2 spot count determination was biased due to the region of the specimen evaluated.

There were 4 additional specimens with CEP17 amplifications and these were classified as unamplified (i.e. HER2-negative). OncoScan detected a copy number of 4 for three of the specimens, suggesting these samples were HER2-positive and the HER2 amplification was masked due to the CEP17 amplification. OncoScan detected a copy number of 3 for the fourth sample (H2N13), which is a single copy amplification; the CEP17 amplification may have also masked this amplification as specimen H2N10 is classified as amplified (HER2/CEP17 ratio = 2.0 and HER2 spot count = 4.3), and also contains a copy number of 3 as determined by OncoScan. One specimen (H2N29) that was classified as unamplified based on CISH, OncoScan reported as CN = 3. This sample did not contain a CEP17 amplification and may represent a false positive. Conversely, this sample may be true positive, but CISH failed to detect the HER2 amplification due to the limited number of cells analyzed.

A second set of samples (6 total) that had been previously characterized by Foundation Medicine or Guardant Health were also analyzed for accuracy. Foundation Medicine and Guardant Health use NGS data to detect CNVs; Foundation Medicine can detect CNVs for any gene on their panel, whereas Guardant analyzes 16 out of 68 genes for CNVs (as of March 2015). Table 7 summarizes the results from OncoScan, Foundation Medicine, and Guardant Health. A total of 24 genes were listed in Foundation Medicine reports as having CNVs and 1 gene was listed in a Guardant Health report. OncoScan produced the same result for 23/25 instances. The discordant calls include:

**Table 7:** OncoScan CNV result comparison to clinical reports from Foundation Medicine and Guardant Health

Sample	Gene	OncoScan Results	Foundation Medicine or Guardant Health Result	Concordant
RD_001_T_C1	MYC	Amp	Amp (F)	Yes
	ATM	Copy Neutral Loss in region containing exons 1-59	Del exons 1-28 (F)	No
	KLHL6	Amp	Amp (F)	Yes
	MCL1	No Change (only 2 probes in 2Mb region of gene)	Del (F)	No
RD_003_T	BCL6	Amp	Amp (F)	Yes
	KLHL6	Amp	Amp (F)	Yes
	PIK3CA	Amp	Amp(F)	Yes
	TERC	Amp	Amp (F)	Yes
	PRKCI	Amp	Amp (F)	Yes
	QKI	Amp	Amp (F)	Yes
	PARK2	Amp	Amp (F)	Yes
RD_007_T	EGFR	Amp	Amp (F)	Yes
	ERBB2	Amp	Amp (F)	Yes
	CCNE1	Amp	Amp (F)	Yes
	CEBPA	Amp	Amp (F)	Yes
	FGF10	Amp	Amp (F)	Yes
	IL7R	Amp	Amp (F)	Yes
	RICTOR	Amp	Amp (F)	Yes
RD_009_T	MYC	Amp	Amp (F)	Yes
	SDHA	Amp	Amp (F)	Yes
RD_017_T	MITF	Amp	Amp (F)	Yes
	FOXP1	Amp	Amp (F)	Yes
	IKZF1	Del	Del (F)	Yes
	EGFR	Del	Del (F)	Yes
RD_014_T	PIK3CA	Amp	Amp (G)	Yes

Legend: Amp = amplification, Del = deletion, (F) = Foundation Medicine and (G) = Guardant Health.

- The MCL1 gene in sample RD001_T_C1 - OncoScan did not report a deletion in this region as the probe density is very low (2 probes within a 2Mb region of the gene). Moreover, OncoScan did not detect any CNVs in the adjacent regions.
- The ATM gene in sample RD001_T_C1 - Foundation reported a homozygous deletion for exons 1−28, whereas OncoScan detected copy neutral loss (LOH in the diploid state) for exons 1–59.

A total of 35 FFPE samples that were previously characterized on the Illumina CytoSNP850K array with a SNP confidence score > 95 were used to determine accuracy of OncoScan. Unlike OncoScan, the CytoSNP850K array was not designed specifically to analyze FFPE samples. Rather than analyze all regions throughout the genome, we restricted the accuracy study to 98 genes that are considered clinically actionable (Supplementary Table S17). Based on the CytoSNP850K data, a total of 25 samples had a CNV in at least one of the 98 genes. OncoScan detected 199 of 201 CNVs, however, there were a total of 72 discordant calls based on classification of the CNV (i.e. CN Gain, CN Loss or LOH). We observed the majority of CNVs occurred in large regions (> 10Mb), so all discordant calls were easily evaluated by analyzing the B allele frequency (BAF) and log-R ratio (logR) plots (Note: A distinction was not made between “Copy Number Gain” and “High Copy Number Gain” calls, so a “Copy Number Gain” call in one platform was considered concordant with “High Copy Number Gain” in the other platform). With the exception of 1 call, all discordant calls were found to be concordant after review of the BAF and logR plots. Examples of resolved discordant calls are included in Supplementary Table S18. In one sample, half the target (ATM gene) was deleted and the other half amplified. In this case, OncoScan classified the event as CN Loss and CytoSNP850K classified as CN Gain.

Concordance was determined by dividing the number of common calls by the total calls (number of genes analyzed) and was 99% or higher for all samples (Table 8). The one discordant call that was not resolved by analysis of BAF and logR plots includes a slight amplification of the *CDK2NA* gene in sample RD_008. The OncoScan logR and BAF plots show LOH, but no amplification. The Genome Studio plots show a slight amplification and LOH in this region.

**Table 8:** OncoScan concordance for 98 actionable genes

Sample	Number of Genes Analyzed	Number of Altered Genes	Discordant Calls	Concordance (%)**
RD_002	98	5	0	100
RD_005A1	98	9	0	100
RD_008	98	17	1	99
1002837_84444	98	4	0	100
1005934	98	5	0	100
1006138_169167	98	19	0	100
1006807_182985	98	12	0	100
1008712-139858	98	10	0	100
1009749-251424	98	3	0	100
1009953-256930	98	4	0	100
1011142	98	1	0	100
1012045-334325	98	7	0	100
1012374_346270	98	1	0	100
1013301-375808	98	9	0	100
1013490-384009	98	12	0	100
1014032-395561	98	9	0	100
1014378-402958	98	3	0	100
1014577-406010	98	2	0	100
3000642-384350	98	5	0	100
3000643-383734	98	8	0	100
BR14-194 05-01T	98	29	0	100
S005-1060-1C-54417	98	8	0	100
S09-259	98	3	0	100
SP11-180 A14	98	14	0	100
Total		199	1	99.5%

Average Concordance (does not include 10 samples w/o calls in the 98 genes).

In total, 263 CNVs determined by other platforms were compared to OncoScan and 2 false negatives (both in sample RD_001_T_C1) were observed. Based on these results (and excluding equivocal FISH cases), the analytical sensitivity (True Positives/True Positives + False Negatives) of the TumorNext panel is 257/257 + 2 = 99.2% (95% CI, 97.24%−99.91%).

### Analytical specificity

The analytical specificity (i.e. the false positive rate) is determined by dividing the true negatives (TN) by the sum of the false positives (FP) and true negatives (TN/FP + TN). We estimated the analytical specificity of OncoScan using the concordance data from the HER2/*neu* study. A total of 29 true negatives (i.e. unamplified) were confirmed with OncoScan with 1 false positive call (H2N29). Overall, the analytical specificity is 29/1 + 29 = 96.7% (95% CI, 82.8% − 99.9%).

We were unable to use the data from the Foundation Medicine reports to calculate the analytical specificity due to the lack of true negatives (i.e. their assay cannot detect amplifications below 5 copies, so the absence of a call for a particular gene does not mean it is 2n). This highlights the challenge of validating a more sensitive platform as OncoScan can detect more CNVs than NGS methods. Moreover, OncoScan provides whole genome CNV status, whereas NGS panels are limited to the number of genes analyzed. While the number of clinically actionable genes based on CNVs is limited, relying on a panel for CNV determination can produce misleading results. This is illustrated in sample RD_017, which exhibits extensive polyploidy (Supplementary Figure S3). Most NGS bioinformatic pipelines include differences in read depth as a mechanism to determine CNV status. In this specimen, almost the entire genome was amplified uniformly to 4n, so there are only a small number of regions where read depth differences can be distinguished. Thus, using an NGS based approach for CNV analysis in polyploidy samples may yield misleading results. Practically every gene was amplified in this specimen and the FoundationOne report only lists 2 (both classified as equivocal); MITF and FOXP1, which are located in the same region of chromosome 3 with a copy number of 6n. A region containing the genes ZNF217, AURKA, GNAS, which are on the FoundationOne panel, was also amplified to 6n but not reported. This is probably due to the fact MITF and FOXP1 are flanked by regions at a lower copy number, whereas the amplified fragment containing ZNF217, AURKA and GNAS is located at the end of the chromosome arm and only flanked by one region at a lower copy number (Supplementary Figure S3).

Samples RD_003 and RD_007 also exhibited extensive polyploidy and have several amplifications ≥ 5× that were not reported by Foundation Medicine. With the exception of ERBB2 (HER2), all focal amplifications detected by NGS appear to be limited to regions surrounded by a dramatic drop in sequencing coverage (Supplementary Figures S4 and S5). OncoScan is capable of detecting more subtle amplifications. For example, RD_007 contains focal amplifications in IDH2, AXIN1, ARFRP1 and SOCS1, clinically actionable genes on Foundation's panel, but the copy number difference to the surrounding areas may be too small for NGS to detect (Supplementary Figure S5). Interestingly, ARFRP1 was recently found to be co-amplified with cycling genes (CCND1, CCND2, CCND3 and CCNE1) and this sample also contains a CCNE1 amplification at 33× [41]. Also, FGF10, IL7R and RICTOR are all reported as CNVs by Foundation Medicine, but SDHA is part of the same amplification (at a copy number of 7 on chromosome 5) and not reported. This may be due to its proximity to the region where a drop in coverage is observed, which is several Mbs away (Supplementary Figure S4). Sample RD_003 contains focal amplifications in RAD51B (partial), VHL, KIT, KDR, PDRGFRA and FANCD2 at a copy number of 4 and PRSS8 amplification at a copy number of 5, all of which are below the level of detection for NGS methods (Supplementary Figure S5), so were not reported by Foundation Medicine. A CDKN1A focal amplification is also present in this sample at a copy number of 6 and was not reported (Supplementary Figure S5).

**Figure 7:**
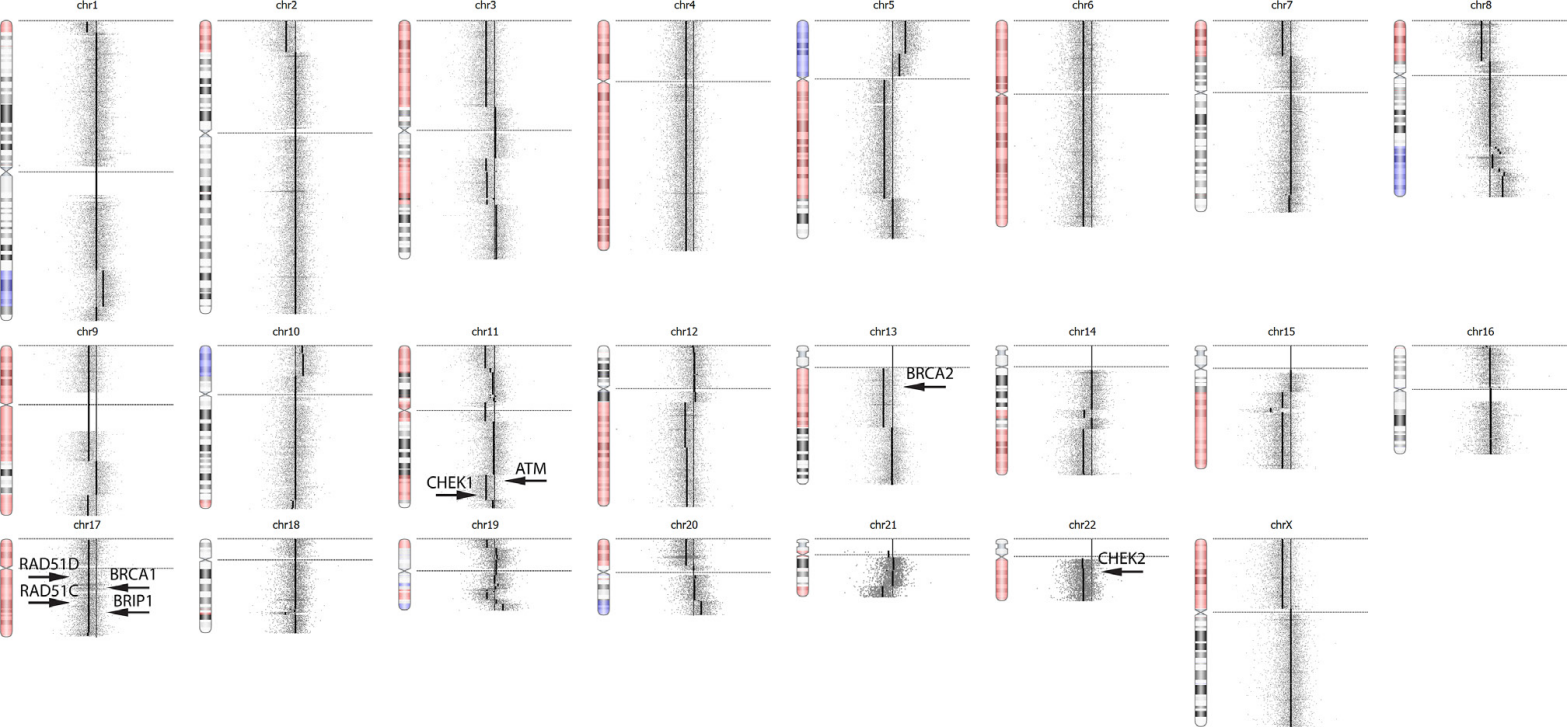
OncoScan Result for RD_009 OncoScan revealed RD_009 has hemizygous deletions in ATM, CHEK1, CHEK2, BRCA1, BRCA2, BRIP1, RAD51C and RAD51D, which represents 8 of the 13 genes involved in homologous recombination DNA repair and suggests this patient is a candidate for PARP inhibitor treatment. These hemizygous deletions were not detected using NGS methods for CNV analysis.

Another advantage of using OncoScan rather than NGS methods for CNV detection in somatic tumors is the ability to detect hemizygous mutations. For hereditary cancer patients, the so-called “second hit” described in the two-hit hypothesis [42] often times is loss of heterozygosity with the mutant allele in a hemizygous state in the tumor, driving disease [43]. The detection of hemizygous deletions in somatic tumors using NGS methods has been demonstrated using whole exome sequencing of tumor and matched blood [44], but remains challenging for gene panels. The clinical significance of detecting hemizygous mutants is illustrated in a recent publication by Mateo el al., which showed response to olaparib (a PARP inhibitor) in a patient with a somatic hemizygous deletion in both BRCA2 and PALB2 [45], homologous recombination (HR) DNA repair genes. Sample RD_001_C1 has a germline mutation in BRCA1 (S1655fs*16) at 49% frequency (in 80% tumor) in the NGS data and hemizygous deletion (i.e. LOH) in BRCA1, suggesting the mutant allele is present in a hemizygous state in the tumor and driver of disease. OncoScan also revealed RD_009 has hemizygous deletions in ATM, CHEK1, CHEK2, BRCA1, BRCA2, BRIP1, RAD51C and RAD51D (Figure 7), which represents 8 of the 13 genes involved in HR DNA repair [46] and suggests this patient is a candidate for PARP inhibitor treatment. These deletions were not reported by Foundation Medicine. This patient also has a somatic frameshift mutation in BRCA1 (p.K1160Lfs*47) at 32% in the NGS data (in 50% tumor), suggesting this allele is present in the tumor in a hemizygous state.

### Precision and reproducibility

OncoScan provides a genome-wide CNV profile, but only a limited number of genes (approximately 98) are considered clinically actionable. For the precision and reproducibility study, we restricted analysis to reported CNVs in these 98 clinically actionable genes. Samples were prepared separately by different technicians on different dates and ran in triplicate as inter-assay replicates (5 samples) and intra-assay (1 sample) replicates. Concordance ranged from 95% to 100% for the inter-assay reproducibility study with lowest concordance observed in 1003748_120089, a specimen with increased heterogeneity (Table 9). The CNV events varied slightly each time a sampling of the DNA pool was taken, resulting in slightly reduced reproducibility. Concordance was 100% for intra-assay reproducibility (Table 10).

**Table 9:** Results for the inter-assay reproducibility study

Sample No	(Val 1) DATES: 10/31/14, 11/7/14, 12/1/14 and 12/10/14 Technician 1	Precision Study (Val 2) DATE: 12/11/14,1/23/15, 2/10/15 Technician 2	Reproducibility Study (Val 3) DATE:1/27/15 Technician 1	Overall Concordance
10-SU4610-B2	73/74 CNVs	74/74 CNVs	72/74 CNVs	99%
1003748_120089	21/22 CNVs	21/22 CNVs	21/22 CNVs	95%^*^
1006138_169167	16/16 CNVs	16/16 CNVs	16/16 CNVs	100%
1013214_374406	82/82 CNVs	82/82 CNVs	81/82 CNVs	99%
1013301_375808	13/13 CNVs	13/13 CNVs	12/13 CNVs	97%

* % Tumor could not be determined for this sample, indicating increased heterogeneity. The CNV events varied slightly each time a sampling of the DNA pool was taken, resulting in slightly reduced reproducibility.

**Table 10:** Results for the intra-assay reproducibility study

Sample No	Date: 2/11/15 Technician 1	Date: 2/11/15 Technician 1	Date: 2/11/15 Technician 1	Overall Concordance
1006138_169167	16/16 CNVs	16/16 CNVs	16/16 CNVs	100%

## DISCUSSION

The ability to classify tumors based on DNA alterations rather than traditional tissue pathology is changing the paradigm of cancer treatment into a more personalized approach. As more targeted therapies are developed and if basket trials show patient benefit from “off-label” use of drugs, there will be an increased need for comprehensive tumor profiling as more patients will be prescribed therapy based on DNA mutations. TumorNext was developed to detect actionable mutations with the added benefit of determining whether a mutation is either somatic or germline. Our validation highlighted cases where knowing the germline status of variants was critical for accurate variant classification. The assay leverages both probe-based target enrichment coupled with NGS for SNV, indel and SV detection, and incorporates use of OncoScan for global CNV detection. Our decision to incorporate this technology stems from the recent observation by Ciriello *et al*. that showed a bias in the type of mutations found in specific tumor types, such that specific tumors contained primarily either CNVs or SNVs/indels, but not both [47]. Breast and ovarian cancers displayed the largest percentage of CNVs in the Ciriello study and make up the vast majority of specimens analyzed at Ambry Genetics. Moreover, OncoScan provides hemizygous deletion status, which is often times the “second hit” for patients with germline mutations.

**Figure 8:**
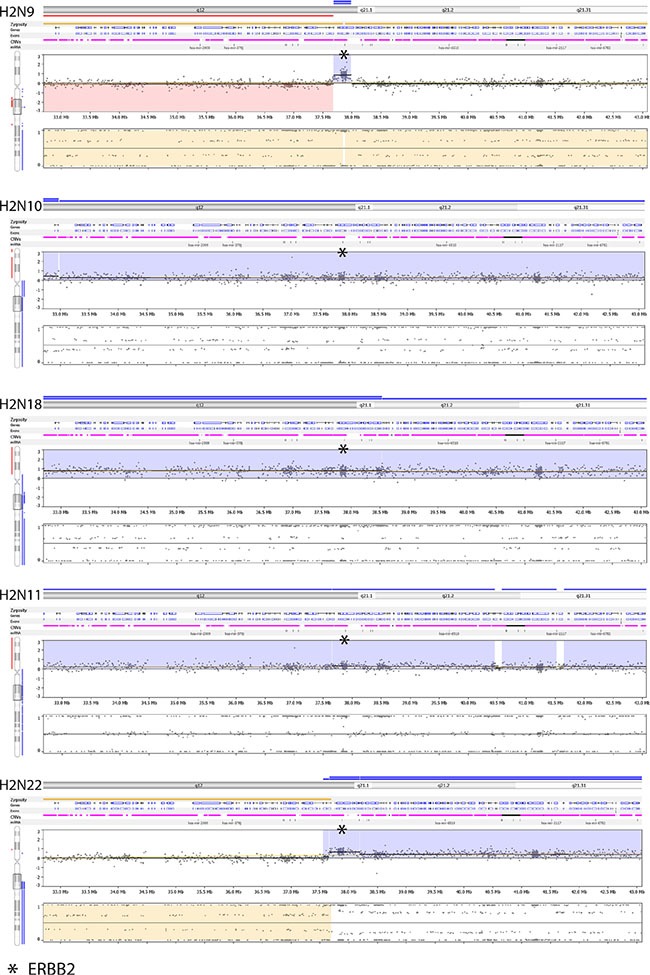
Nexus screenshots of HER2-positive specimens ran on OncoScan Only two (H2N9 and H2N22) out of the five have a focal amplification and one of them is adjacent to a large event (H2N22 – ERBB2 copy number is 6, which is flanked by normal (CN = 2) and amplified (CN = 4) regions.

Most of the CNVs detected in the validation samples, including HER2-postive samples, consisted of either whole or partial (i.e. quarter to half) chromosome copy number changes and most samples had an excessive number of CNVs. As a result, the clinical reporting of CNVs is restricted to genes listed in Supplementary Table S17 as well as all HR DNA repair genes. All CNVs reported by OncoScan for these genes are manually reviewed. Moreover, deletions (both homo and hemizygous) are only reported for tumor suppressor and DNA repair genes, and only focal amplifications greater than 3 are reported for oncogenes. However, this brings into question the ISH HER2-postive cases that were compared to OncoScan as only 2 of the 5 positive cases contained a focal amplification. The other 3 specimens were classified as HER2-positive based on ISH, but the ERBB2 amplifications were part of a large event (Figure 8). One possible explanation for the difference may be that the assignment of these cases as HER2-positve may not be correct since ERBB2 was not selectively amplified.

TumorNext has been launched in our clinical lab and initial results have identified actionable germline and somatic mutations (SNVs, CNVs and gene fusions) for most cases submitted. Ambry has decided to follow the ACMG recommendations for incidental findings and automatically reports germline findings in 20 genes on TumorNext that fall within the ACMG gene list [48]. Ambry's client base primarily consists of treating physicians that routinely order germline testing, so we have not encountered a situation where the treating physician does not want to know the germline status of the ACMG genes. Patients who do not want to know their germline status are eligible for TumorNext, but this will require the treating physician to withhold the germline results. Also, unlike Ambry's germline tests, such as CancerNext, germline findings identified by TumorNext are not Sanger confirmed and only germline variants classified as pathogenic and likely pathogenic are included on the TumorNext report. The TAT is 2–3 weeks, which is comparable to other somatic tests on the market.

The described validation was conducted primarily with FFPE tumor blocks and slides using approximately 50 uM of tissue at > 20% tumor tissue. This amount of tissue was sufficient to yield a minimum of 500 – 1000 ng of DNA, which is the optimal DNA input for NGS library prep for bait-capture protocols, so this was established as tissue requirements for TumorNext. DNA extracted from core-needle biopsies has been analyzed with TumorNext using 100ng input, but the assay was not optimized for low DNA input. Only a portion of the DNA added to a reaction is analyzed as not all DNA fragments are converted successfully to the NGS library (i.e. not all input DNA undergoes successful ligation and amplification). Moreover, DNA extracted from FFPE somatic tumors is heterogeneous and will have varying degrees of damage that may limit amplification. It is essential that sufficient DNA input is used to compensate for the DNA damage and heterogeneity in FFPE specimens to provide an unbiased, representative sampling of the total DNA. The limited amount of DNA isolated from core-needle biopsies can exacerbate this problem. As a result, we are developing a separate protocol for low input FFPE samples to limit sampling bias.

## MATERIALS AND METHODS

### Validation samples

For the validation, two types of samples were used: 1) HapMap DNA samples and 2) genomic DNA isolated from FFPE tissue (tumor and normal) from the following cancer types: breast, ovarian, uterine, colon, kidney, liver, melanoma, head and neck. All FFPE specimens had been previously characterized by either Illumina CytoSNP850K array, Sanger Sequencing, NGS panel (FoundationOne^™^, Guardant Health or Caris), OncoScan hotspot panel, FISH, CISH, IHC, qPCR or multiple methods. In addition, a matched blood sample was included when available. All FFPE specimens were reviewed by a pathologist and contained ≥ 20% tumor cellularity.

### NGS gene panel design and capture protocol

A custom panel was designed to analyze 2,350 exons, 153 introns and 4 UTR regions in 142 genes associated with solid tumor cancers (Supplementary Table S18). Approximately 99 genes are frequently mutated in somatic solid tumors and may be targets of FDA-approved or experimental therapies, or prognostic indicators. The remaining 43 genes are associated with hereditary cancers and the mutational status of a subset of these genes may guide treatment (i.e. BRCA1 status and PARP inhibitors). The assay consists of a hybridization-based capture/target enrichment and sequence analysis using massively parallel sequencing. The panel is composed of 9,564 biotinylated xGen Lockdown probes synthesized by Integrated DNA Technologies (IDT, Coralville, IA).

DNA was extracted from tumor and normal tissue FFPE blocks using the Qiagen GeneRead FFPE DNA extraction kit (Qiagen, Santa Clarita, CA), which has a repair step to minimize C to T transitions by removing deanimated cytosines. NGS libraries were constructed according to the protocol outlined by KAPA Biosystems. Briefly, DNA was sheared to an average size of 250-350 bp using sonication (Covaris). DNA fragment ends were repaired and phosphorylated using Klenow, T4 DNA Polymerase, and T4 Polynucleotide Kinase. An ‘A’ base was added to the 3′ end of the blunted fragments, followed by ligation of single-indexed adapters via T-A mediated ligation. The library was PCR-amplified using 8 cycles, and tumor and normal libraries were pooled together at a 20:1 ratio (228ng tumor sample library and 12 ng matched control). Five Tumor/Normal libraries were pooled together prior to capture and incubated with IDT xGen Lockdown probes and blocking oligos for 16–24 hours at 65°C. Captured DNA was washed, eluted and PCR amplified using 10 cycles. The size and concentration of the amplified captured DNA library were determined using the Agilent TapeStation (Agilent Technologies, Santa Clara, CA). All samples were sequenced on an Illumina HiSeq2500, which generated 100 × 100 paired-end reads.

### Bioinformatic analysis

Demultiplexing by barcode and sequence quality filtering was done in the Illumina Consensus Assessment of Sequence and Variation (CASAVA) software (v.1.8.2, Illumina, Hayward, CA). A custom bioinformatics pipeline was developed to perform paired analysis of tumor and germline DNA. Briefly, FASTQ files from CASAVA were aligned to the hg19 version of the human genome using Novoalign V3.02.07. Next, paired-sample analysis was performed using VarScan2 (v2.3.8). For both SNP and indel calling by VarScan2, the minimum variant frequency was set to 1% and the minimum coverage in tumor and normal was set to 6× and 4× respectively. Optimized variant calling filters were set at a read coverage of ≥ 100× for tumor DNA and ≥ 10× for matched control. Gene fusions including deletions, tandem duplications, inversions and translocations were detected using DELLY (v0.6.1). The minimum pair-end mapping quality was set at 1 while other settings were set at default. DELLY filtering required at least 3 paired-end reads supporting the fusion and paired-end mapping quality ≥ 20. SV calls by DELLY were filtered out if determined to be due to repetitive regions. Paired normal samples were also analyzed using a custom bioinformatics pipeline that utilizes Novoalign V3.02.07 to align FASTQ reads to a reference sequence (hg19) and GATK (V3.2.2) to generate variants and no/low coverage reports. Germline variants were filtered using a Q score of ≥ 30, coverage of ≥ 10×, het ratio of ≥ 10% and filtered out if determined to be a sequencing artifact or common polymorphism utilizing population frequency data from multiple sources including NCBI dbSNP, NHLBI Exome Sequencing Project (ESP), 1000 Genomes, and internal Ambry data. Known causative variants outside reportable range are also protected from filtering. For quality control, the pipeline generates coverage metrics including: 1) number of total read pairs, 2) % of mapped read pairs, 3) % of PCR duplicates, 4) number of on-target read-pairs, 5) average coverage in target region, 6) target specificity and 7) % of bases at ≥ 10×, ≥ 20×, ≥ 50×, ≥ 100×, ≥ 200×, ≥ 500×, and ≥ 1000×.

Annotation of variants was performed using custom scripts based on recommendations from the Human Genome Variation Society (HGVS). Gene fusions were annotated using Oncofuse v1.0.7 (ref: doi: 10.1093/bioinformatics/btt445) to report only gene fusions where one of the two fusion partners is in our target genes.

The TumorNext pipeline was designed to achieve maximum sensitivity in detecting somatic variants in tumor samples whether matched control samples are available or not. In tumor-normal analysis mode, we applied Varscan2 (v2.3.6), a highly sensitive, heuristic based algorithm to detect somatic variants as low as 3% minor allele frequency. Current efforts are focused on developing custom filters to remove low confidence calls with evidence from literature and public repositories. For structural variants, we applied DELLY v.0.6.1 to identify potential breakpoints supported by both paired-end and split reads from tumor sequence data.

### OncoScan

The OncoScan workflow is based on the hybridization of MIPs to FFPE DNA samples and subsequent circularization, amplification and labeling. The labeled MIPs are hybridized to the OncoScan array, washed and scanned. The assays were set up according to the OncoScan sample preparation manual (P/N 703175 Rev. 1) using DNA isolated from FFPE tumor specimens using the Qiagen GeneRead FFPE DNA extraction kit (Qiagen, Santa Clarita, CA). Briefly, DNA samples are normalized to 12 ng/μL, mixed with MIPs and incubated overnight to anneal (16–18 hours). Next, each reaction was divided equally into A and B reactions and “Gap Fill” master mix was added with either AT dNTPs (A reaction) or GC dNTPs (B reaction) and incubated. Following the “Gap Fill” reaction, exonuclease was added to remove unligated probes and genomic DNA. Next, MIPs were linearized with a restriction enzyme and PCR amplified (PCR 1). Reactions were taken through a second round of amplification (PCR 2) and subsequently digested with HaeIII restriction enzyme. The digested products were hybridized to the OncoScan Array for 16–18 hrs. Arrays were stained and washed using the GeneChip^®^ Fluidics Station 450 and loaded on the GeneChip^®^ Scanner 3000 7G (Affymetrix, Santa Clara, CA) where fluorescence intensity was scanned to generate array images (DAT files). Next, array fluorescence intensity data (CEL) files were generated and used to produce OSCHP-TuScan files with the OncoScan^®^ Console software version 1.1 using the reference files OncoScan.FFPE.n33.r1.REF_MODEL for CNVs and OncoScan.FFPE.n33.r1.SOM_REF_MODEL for SNPs.

The TuScan algorithm is based on the ASCAT (allele-specific copy number analysis of tumors) algorithm, which determines allele specific copy number and simultaneously estimates and adjusts for both percent tumor and ploidy [49]. It provides copy number in log2 and linear scale, which can be viewed in Nexus Express for OncoScan. OncoScan uses the logR and BAF to determine copy number. The logR ratio is the logged ratio of observed probe intensity to the expected intensity – any deviations from zero indicate copy number change. BAF allows detection of allelic imbalance. A value near 0.5 indicates a heterozygous genotype (AB), whereas 0 and 1 indicate a homozygous genotype (AA and BB) – in a normal diploid sample, there is a mix of AA, AB and BB genotypes. Deletions, copy neutral loss of heterozygosity, imbalanced amplifications and mosaic samples exhibit altered BAF plots. Unlike algorithms that use uniform thresholds, TuScan can detect CNVs when only present in a minority of cells as the algorithm determines what deviations from logR and BAF are consistent with the percent tumor and ploidy of the sample.

## SUPPLEMENTARY MATERIALS FIGURES AND TABLES





























## References

[1] Igbokwe A, Lopez-Terrada DH (2011). Molecular testing of solid tumors. Arch Pathol Lab Med.

[2] McCourt CM, Boyle D, James J, Salto-Tellez M (2013). Immunohistochemistry in the era of personalised medicine. J Clin Pathol.

[3] Pauletti G, Dandekar S, Rong H, Ramos L, Peng H, Seshadri R, Slamon DJ (2000). Assessment of methods for tissue-based detection of the HER-2/neu alteration in human breast cancer: a direct comparison of fluorescence *in situ* hybridization and immunohistochemistry. J Clin Oncol.

[4] Lebeau A, Deimling D, Kaltz C, Sendelhofert A, Iff A, Luthardt B, Untch M, Lohrs U (2001). Her-2/neu analysis in archival tissue samples of human breast cancer: comparison of immunohistochemistry and fluorescence *in situ* hybridization. J Clin Oncol.

[5] Angulo B, Garcia-Garcia E, Martinez R, Suarez-Gauthier A, Conde E, Hidalgo M, Lopez-Rios F (2010). A commercial real-time PCR kit provides greater sensitivity than direct sequencing to detect KRAS mutations: a morphology-based approach in colorectal carcinoma. J Mol Diagn.

[6] Gonzalez de Castro D, Angulo B, Gomez B, Mair D, Martinez R, Suarez-Gauthier A, Shieh F, Velez M, Brophy VH, Lawrence HJ, Lopez-Rios F (2012). A comparison of three methods for detecting KRAS mutations in formalin-fixed colorectal cancer specimens. Br J Cancer.

[7] Lopez-Rios F, Angulo B, Gomez B, Mair D, Martinez R, Conde E, Shieh F, Vaks J, Langland R, Lawrence HJ, de Castro DG (2013). Comparison of testing methods for the detection of BRAF V600E mutations in malignant melanoma: pre-approval validation study of the companion diagnostic test for vemurafenib. PLoS One.

[8] Solin LJ, Gray R, Baehner FL, Butler SM, Hughes LL, Yoshizawa C, Cherbavaz DB, Shak S, Page DL, Sledge GW, Davidson NE, Ingle JN (2013). A multigene expression assay to predict local recurrence risk for ductal carcinoma *in situ* of the breast. J Natl Cancer Inst.

[9] Wagle N, Berger MF, Davis MJ, Blumenstiel B, Defelice M, Pochanard P, Ducar M, Van Hummelen P, Macconaill LE, Hahn WC, Meyerson M, Gabriel SB, Garraway LA (2011). High-throughput detection of actionable genomic alterations in clinical tumor samples by targeted, massively parallel sequencing. Cancer Discov.

[10] Frampton GM, Fichtenholtz A, Otto GA, Wang K, Downing SR, He J, Schnall-Levin M, White J, Sanford EM, An P, Sun J, Juhn F, Brennan K (2013). Development and validation of a clinical cancer genomic profiling test based on massively parallel DNA sequencing. Nat Biotechnol.

[11] Cottrell CE, Al-Kateb H, Bredemeyer AJ, Duncavage EJ, Spencer DH, Abel HJ, Lockwood CM, Hagemann IS, O'Guin SM, Burcea LC, Sawyer CS, Oschwald DM, Stratman JL (2014). Validation of a next-generation sequencing assay for clinical molecular oncology. J Mol Diagn.

[12] Pritchard CC, Salipante SJ, Koehler K, Smith C, Scroggins S, Wood B, Wu D, Lee MK, Dintzis S, Adey A, Liu Y, Eaton KD, Martins R (2014). Validation and implementation of targeted capture and sequencing for the detection of actionable mutation, copy number variation, and gene rearrangement in clinical cancer specimens. J Mol Diagn.

[13] (2015). “Next Generation” Sequencing (NGS) guidelines for somatic genetic variant detection.

[14] Mamanova L, Coffey AJ, Scott CE, Kozarewa I, Turner EH, Kumar A, Howard E, Shendure J, Turner DJ (2010). Target-enrichment strategies for next-generation sequencing. Nat Methods.

[15] Smith EN, Jepsen K, Khosroheidari M, Rassenti LZ, D'Antonio M, Ghia EM, Carson DA, Jamieson CH, Kipps TJ, Frazer KA (2014). Biased estimates of clonal evolution and subclonal heterogeneity can arise from PCR duplicates in deep sequencing experiments. Genome Biol.

[16] Jones S, Anagnostou V, Lytle K, Parpart-Li S, Nesselbush M, Riley DR, Shukla M, Chesnick B, Kadan M, Papp E, Galens KG, Murphy D, Zhang T (2015). Personalized genomic analyses for cancer mutation discovery and interpretation. Sci Transl Med.

[17] Hodgson S (2008). Mechanisms of inherited cancer susceptibility. J Zhejiang Univ Sci B.

[18] Parsons DW, Roy A, Plon SE, Roychowdhury S, Chinnaiyan AM (2014). Clinical tumor sequencing: an incidental casualty of the American College of Medical Genetics and Genomics recommendations for reporting of incidental findings. J Clin Oncol.

[19] Pinkel D, Segraves R, Sudar D, Clark S, Poole I, Kowbel D, Collins C, Kuo WL, Chen C, Zhai Y, Dairkee SH, Ljung BM, Gray JW (1998). High resolution analysis of DNA copy number variation using comparative genomic hybridization to microarrays. Nat Genet.

[20] Pollack JR, Perou CM, Alizadeh AA, Eisen MB, Pergamenschikov A, Williams CF, Jeffrey SS, Botstein D, Brown PO (1999). Genome-wide analysis of DNA copy-number changes using cDNA microarrays. Nat Genet.

[21] Hagenkord JM, Chang CC (2009). The rewards and challenges of array-based karyotyping for clinical oncology applications. Leukemia.

[22] Gunn SR, Mohammed MS, Gorre ME, Cotter PD, Kim J, Bahler DW, Preobrazhensky SN, Higgins RA, Bolla AR, Ismail SH, de Jong D, Eldering E, van Oers MH (2008). Whole-genome scanning by array comparative genomic hybridization as a clinical tool for risk assessment in chronic lymphocytic leukemia. J Mol Diagn.

[23] Lyons-Weiler M, Hagenkord J, Sciulli C, Dhir R, Monzon FA (2008). Optimization of the Affymetrix GeneChip Mapping 10K 2. 0 Assay for routine clinical use on formalin-fixed paraffin-embedded tissues. Diagn Mol Pathol.

[24] Lo J, Kerns BJ, Amling CL, Robertson CN, Layfield LJ (1996). Correlation of DNA ploidy and histologic diagnosis from prostate core-needle biopsies: is DNA ploidy more sensitive than histology for the diagnosis of carcinoma in small specimens?. J Surg Oncol.

[25] Koivisto P (1997). Aneuploidy and rapid cell proliferation in recurrent prostate cancers with androgen receptor gene amplification. Prostate Cancer Prostatic Dis.

[26] Reinholz MM, Bruzek AK, Visscher DW, Lingle WL, Schroeder MJ, Perez EA, Jenkins RB (2009). Breast cancer and aneusomy 17: implications for carcinogenesis and therapeutic response. The Lancet.

[27] Krishnamurti U, Hammers JL, Atem FD, Storto PD, Silverman JF (2009). Poor prognostic significance of unamplified chromosome 17 polysomy in invasive breast carcinoma. Mod Pathol.

[28] Watters AD, Going JJ, Cooke TG, Bartlett JM (2003). Chromosome 17 aneusomy is associated with poor prognostic factors in invasive breast carcinoma. Breast Cancer Res Treat.

[29] Mehta S, Shelling A, Muthukaruppan A, Lasham A, Blenkiron C, Laking G, Print C (2010). Predictive and prognostic molecular markers for cancer medicine. Ther Adv Med Oncol.

[30] Heppner GH (1984). Tumor heterogeneity. Cancer research.

[31] Marusyk A, Polyak K (2010). Tumor heterogeneity: causes and consequences. Biochimica et biophysica acta.

[32] Lauring AS, Andino R (2010). Quasispecies theory and the behavior of RNA viruses. PLoS Pathog.

[33] Shah SP, Morin RD, Khattra J, Prentice L, Pugh T, Burleigh A, Delaney A, Gelmon K, Guliany R, Senz J, Steidl C, Holt RA, Jones S (2009). Mutational evolution in a lobular breast tumour profiled at single nucleotide resolution. Nature.

[34] Cheng DT, Mitchell TN, Zehir A, Shah RH, Benayed R, Syed A, Chandramohan R, Liu ZY, Won HH, Scott SN, Brannon AR, O'Reilly C, Sadowska J (2015). Memorial Sloan Kettering-Integrated Mutation Profiling of Actionable Cancer Targets (MSK-IMPACT): A Hybridization Capture-Based Next-Generation Sequencing Clinical Assay for Solid Tumor Molecular Oncology. J Mol Diagn.

[35] Samuels Y, Wang Z, Bardelli A, Silliman N, Ptak J, Szabo S, Yan H, Gazdar A, Powell SM, Riggins GJ, Willson JK, Markowitz S, Kinzler KW (2004). High frequency of mutations of the PIK3CA gene in human cancers. Science (New York, NY.

[36] Bose R, Kavuri SM, Searleman AC, Shen W, Shen D, Koboldt DC, Monsey J, Goel N, Aronson AB, Li S, Ma CX, Ding L, Mardis ER (2013). Activating HER2 mutations in HER2 gene amplification negative breast cancer. Cancer Discov.

[37] Wang Y, Carlton VE, Karlin-Neumann G, Sapolsky R, Zhang L, Moorhead M, Wang ZC, Richardson AL, Warren R, Walther A, Bondy M, Sahin A, Krahe R (2009). High quality copy number and genotype data from FFPE samples using Molecular Inversion Probe (MIP) microarrays. BMC Med Genomics.

[38] Wolff AC, Hammond ME, Hicks DG, Dowsett M, McShane LM, Allison KH, Allred DC, Bartlett JM, Bilous M, Fitzgibbons P, Hanna W, Jenkins RB, Mangu PB (2013). Recommendations for human epidermal growth factor receptor 2 testing in breast cancer: American Society of Clinical Oncology/College of American Pathologists clinical practice guideline update. J Clin Oncol.

[39] Gunn S, Yeh IT, Lytvak I, Tirtorahardjo B, Dzidic N, Zadeh S, Kim J, McCaskill C, Lim L, Gorre M, Mohammed M (2010). Clinical array-based karyotyping of breast cancer with equivocal HER2 status resolves gene copy number and reveals chromosome 17 complexity. BMC Cancer.

[40] Sapino A, Goia M, Recupero D, Marchio C (2013). Current Challenges for HER2 Testing in Diagnostic Pathology: State of the Art and Controversial Issues. Front Oncol.

[41] Schwaederle M, Daniels GA, Piccioni DE, Fanta PT, Schwab RB, Shimabukuro KA, Parker BA, Kurzrock R (2015). Cyclin alterations in diverse cancers: Outcome and co-amplification network. Oncotarget.

[42] Knudson AG (1971). Mutation and cancer: statistical study of retinoblastoma. Proc Natl Acad Sci USA.

[43] Knudson AG (2001). Two genetic hits (more or less) to cancer. Nat Rev Cancer.

[44] Grasso CS, Wu YM, Robinson DR, Cao X, Dhanasekaran SM, Khan AP, Quist MJ, Jing X, Lonigro RJ, Brenner JC, Asangani IA, Ateeq B, Chun SY (2012). The mutational landscape of lethal castration-resistant prostate cancer. Nature.

[45] Mateo J, Carreira S, Sandhu S, Miranda S, Mossop H, Perez-Lopez R, Nava Rodrigues D, Robinson D, Omlin A, Tunariu N, Boysen G, Porta N, Flohr P (2015). DNA-Repair Defects and Olaparib in Metastatic Prostate Cancer. N Engl J Med.

[46] Pennington KP, Walsh T, Harrell MI, Lee MK, Pennil CC, Rendi MH, Thornton A, Norquist BM, Casadei S, Nord AS, Agnew KJ, Pritchard CC, Scroggins S (2014). Germline and somatic mutations in homologous recombination genes predict platinum response and survival in ovarian, fallopian tube, and peritoneal carcinomas. Clin Cancer Res.

[47] Ciriello G, Miller ML, Aksoy BA, Senbabaoglu Y, Schultz N, Sander C (2013). Emerging landscape of oncogenic signatures across human cancers. Nat Genet.

[48] Green RC, Berg JS, Grody WW, Kalia SS, Korf BR, Martin CL, McGuire A, Nussbaum RL, O'Daniel JM, Ormond KE, Rehm HL, Watson MS, Williams MS (2013). ACMG Recommendations for Reporting of Incidental Findings in Clinical Exome and Genome Sequencing. Genet Med.

[49] Van Loo P, Nordgard SH, Lingjaerde OC, Russnes HG, Rye IH, Sun W, Weigman VJ, Marynen P, Zetterberg A, Naume B, Perou CM, Borresen-Dale AL, Kristensen VN (2010). Allele-specific copy number analysis of tumors. Proc Natl Acad Sci USA.

