# Supplementary material for: TumorNext: A comprehensive tumor profiling assay that incorporates high resolution copy number analysis and germline status to improve testing accuracy
Source: Oncotarget. 2016 Sep 8;7(42):68206–28. doi: 10.18632/oncotarget.11910 (PMC5356550; doi:10.18632/oncotarget.11910)
Supplement: Supplementary file 1 [file oncotarget-07-68206-s001.pdf]

TumorNext: A comprehensive tumor profiling assay that incorporates high resolution copy number analysis and germline status to improve testing accuracy

Supplementary Materials

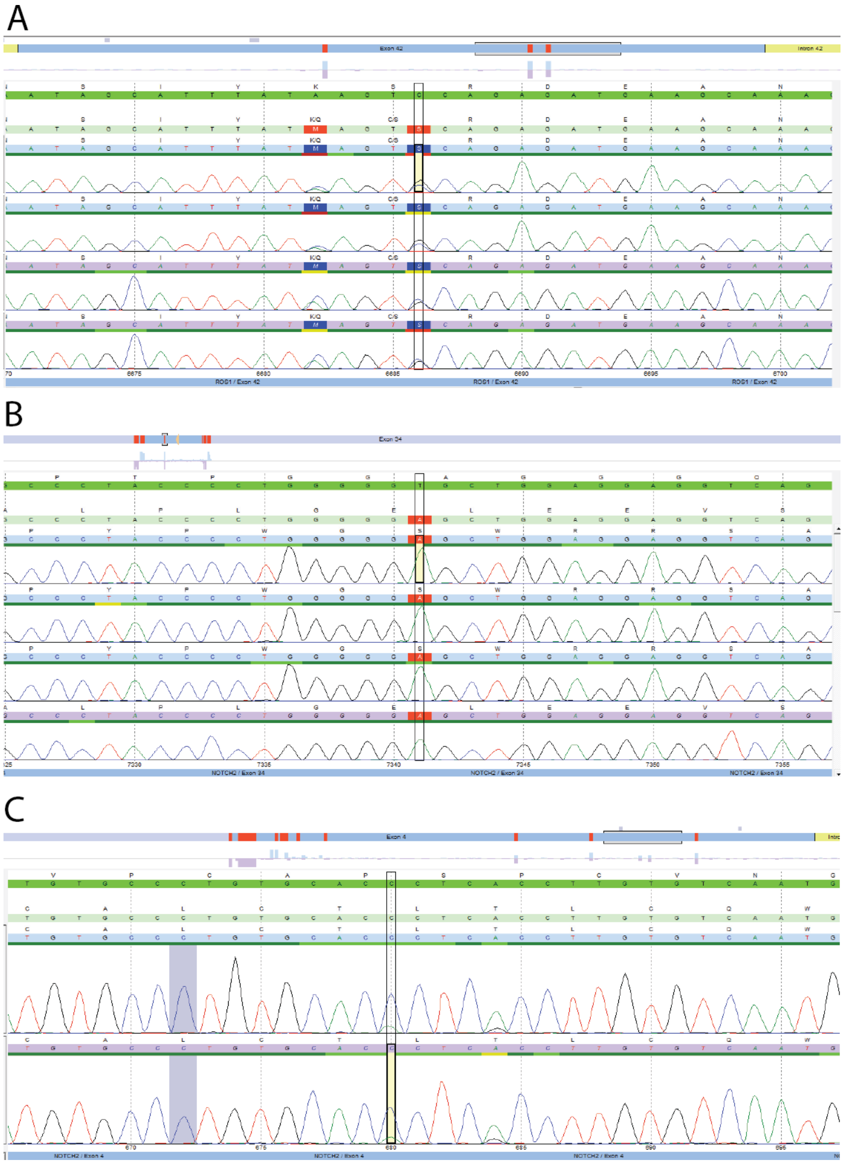

Supplementary Figure S1: Sanger Sequencing Results for HapMap sample NA07019. The reference genotype for SNPs rs619203 (**A**) rs6685892 (**B**) and rs3899528 (**C**) were shown to be discordant with TumorNext and Sanger sequencing data. The reference genotype for rs619203 is GG, but TumorNext and Sanger results are GC. The reference genotype for rs6685892 is AT, but TumorNext and Sanger results are TT/AA. The reference genotype for rs3899528 is GG, but TumorNext and Sanger results are GT/CA.

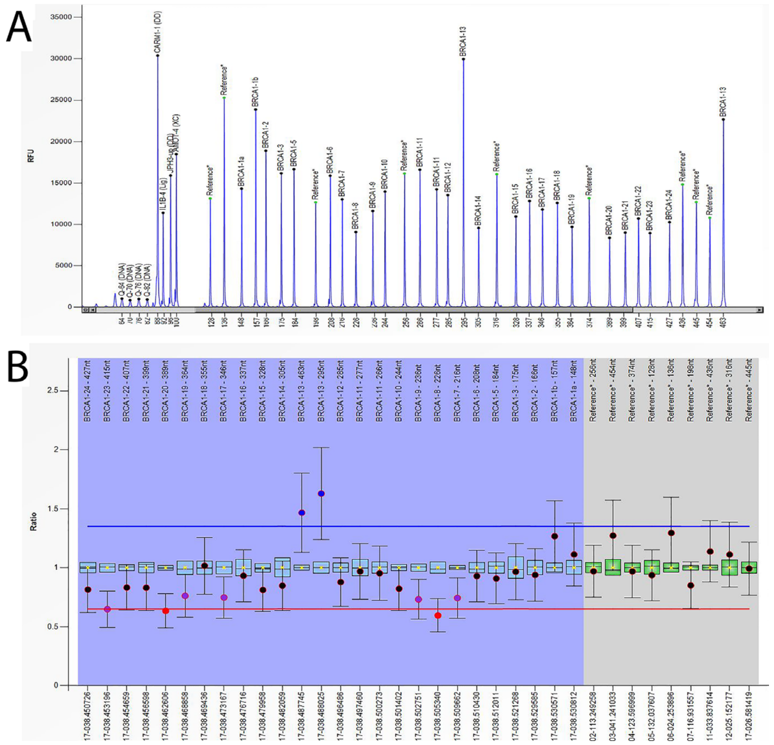

Supplementary Figure S2: MLPA (Multiplex Ligation-dependent Probe Amplification) for sample containing the BRCA1 exon-13 6kb duplication. Data was analyzed using Coffalyser software, which shows elevated relative fluorescence units (RFUs) for exon 13 (BRCA1-13) (**A**) and an elevated ratio for two BRCA1 exon 13 targets (BRCA1-13-463nt and BRCA1-13-295nt) when compared to a wild type control (**B**).

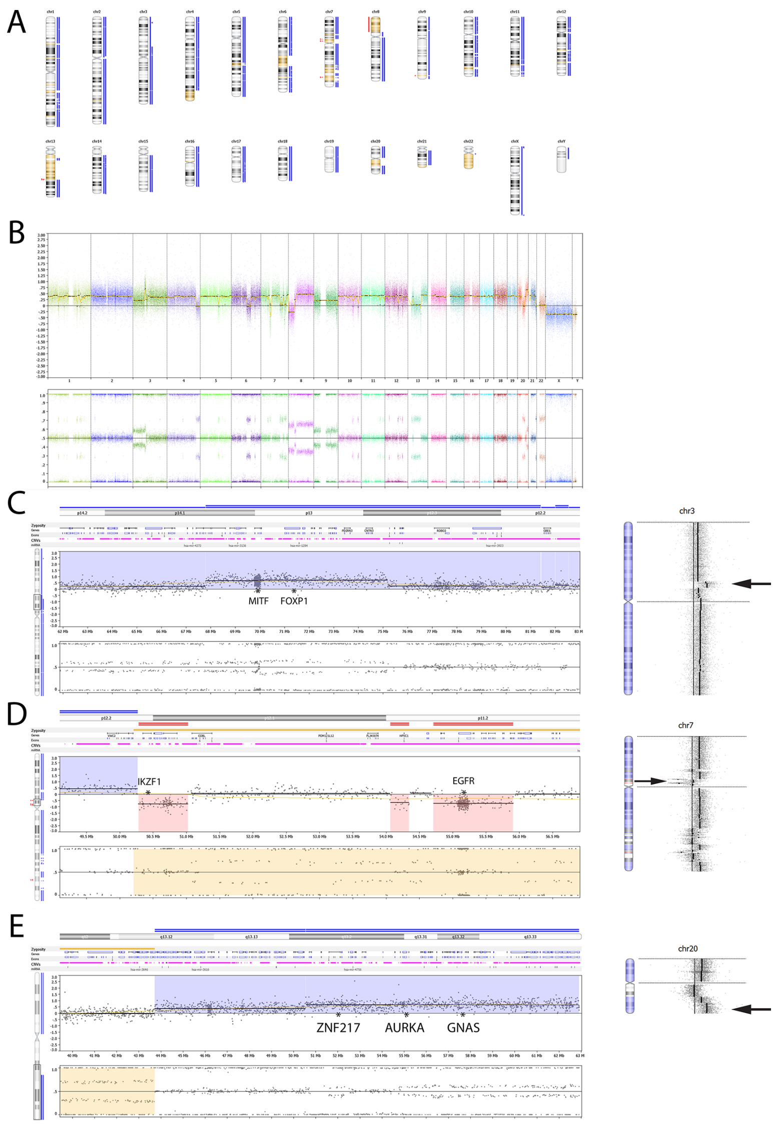

Supplementary Figure S3: OncoScan Result for RD_017. Karyotype (**A**), logR and BAF plots (**B**) show extensive polyploidy. Foundation Medicine reported amplifications for the genes MITF and FOXP1 (**C**) and a deletion for EGFR (**D**). These genes are surrounded by regions with significant differences in copy number (i.e. MITF and FOXP1 are located in a region at 6n, which is surround by regions of 4n and EGFR is in a region with a homozygous deletion surrounded by regions of 4n). A region containing the genes ZNF217, AURKA, GNAS was amplified to 6n (**E**), but was not reported. These genes are located at the end of the chromosome arm and only flanked by one region at a lower copy number.

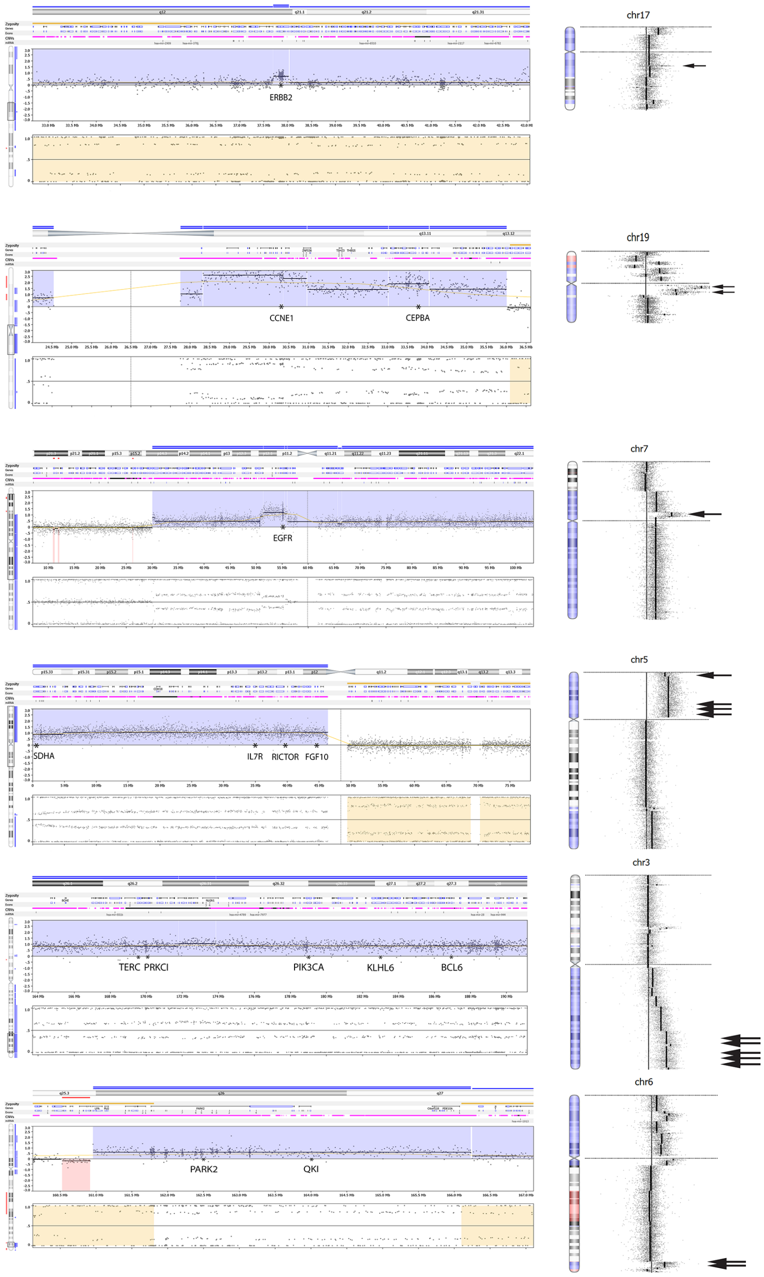

Supplementary Figure S4: OncoScan Result for RD_007. RD_007 contains focal amplifications in IDH2, AXIN1, ARFRP1 and SOCS1, clinically actionable genes on Foundation's panel, but the copy number difference to the surrounding areas may too small for NGS to detect. Interestingly, ARFRP1 was recently found to be co-amplified with cycling genes (CCND1, CCND2, CCND3 and CCNE1) and this sample also contains a CCNE1 amplification at 33×. Also, FGF10, IL7R and RICTOR are all reported as CNVs by Foundation Medicine, but SDHA is part of the same amplification (at a copy number of 7 on chromosome 5) and not reported. This may be due to its proximity to the region where a drop in coverage is observed, which is several Mbs away.

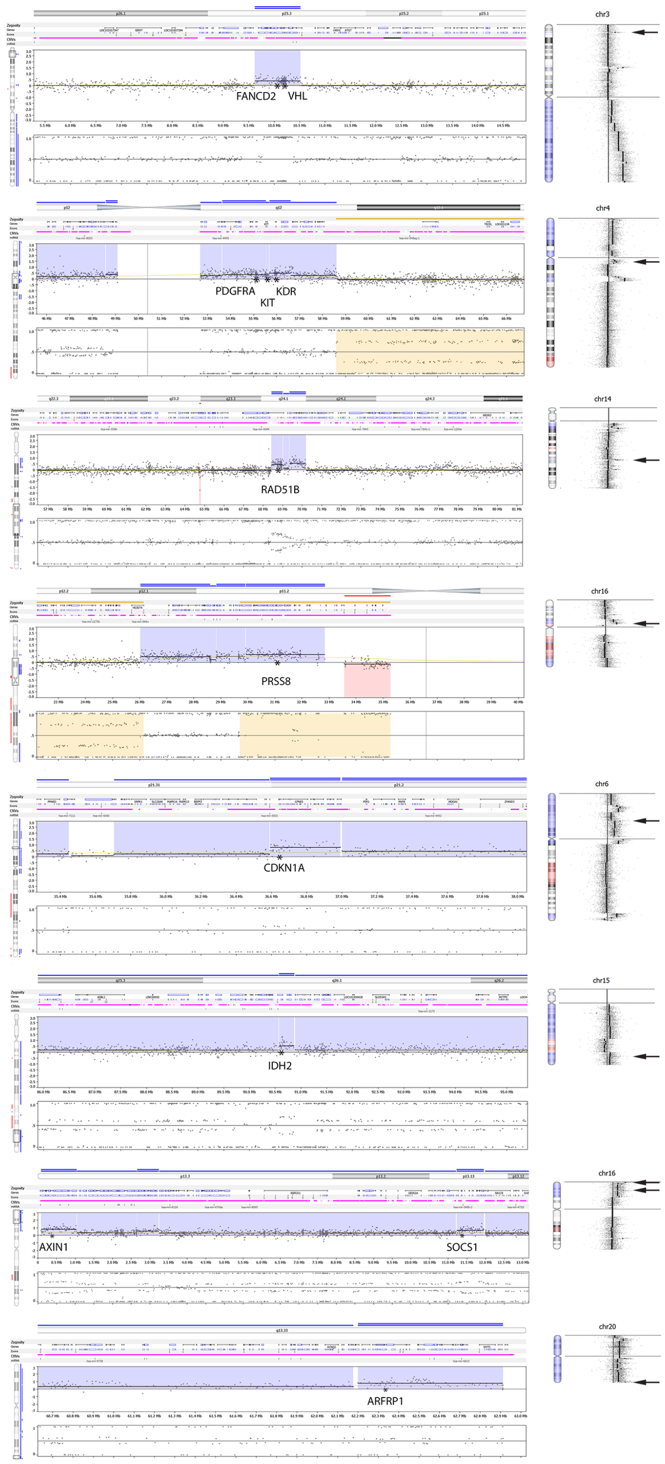

Supplementary Figure S5: OncoScan Result for Sample RD_003. RD_003 contains focal amplifications in RAD51B (partial), VHL, KIT, KDR, PDRGFRA and FANCD2 at a copy number of 4 and PRSS8 amplification at a copy number of 5, all of which are below the level of detection for NGS methods, so were not reported by Foundation Medicine. A CDKN1A focal amplification is also present in this sample at a copy number of 6, which should be detected by NGS methods, but was not reported.

Supplementary Table S1: Simulated SNV dataset

	Allele Frequencies							
Coverage	0 – 3%	3% – 5%	5% –10%	10% – 20%	20% – 30%	30% – 50%	50% – 80%	80% – 100%
**100×**	4,815	8,420	6,677	6,653	6,662	6,643	6,665	6,648
**250×**	6,796	6,794	6,787	6,764	6,777	6,780	6,757	6,817
**500×**	6,815	6,842	6,834	6,837	6,835	6,823	6,805	6,838
**1000×**	6,866	6,828	6,864	6,852	6,875	6,871	6,863	6,855
**Total**	25,292	28,884	27,162	27,106	27,149	27,117	27,090	27,158

Values indicate the number of simulated random test fragments.

Supplementary Table S2: Simulated SNV sensitivity

	Allele Frequencies							
Coverage	0 – 3%	3% – 5%	5% – 10%	10% – 20%	20% – 30%	30% – 50%	50% – 80%	80% – 100%
**100×**	80.04%	88.57%	99.93%	100.00%	100.00%	100.00%	100.00%	100.00%
**250×**	90.38%	99.26%	100.00%	100.00%	100.00%	100.00%	100.00%	100.00%
**500×**	99.91%	100.00%	100.00%	100.00%	100.00%	100.00%	100.00%	100.00%
**1000×**	99.97%	100.00%	100.00%	100.00%	100.00%	100.00%	100.00%	100.00%

Supplementary Table S3: Simulated SNV Specificity

	Allele Frequencies							
Coverage	0 – 3%	3% – 5%	5% – 10%	10% – 20%	20% – 30%	30% – 50%	50% – 80%	80% – 100%
**100×**	100.00%	100.00%	100.00%	100.00%	100.00%	100.00%	100.00%	100.00%
**250×**	100.00%	100.00%	100.00%	100.00%	100.00%	100.00%	100.00%	100.00%
**500×**	100.00%	100.00%	100.00%	100.00%	100.00%	100.00%	100.00%	100.00%
**1000×**	100.00%	100.00%	100.00%	100.00%	100.00%	100.00%	100.00%	100.00%

Supplementary Table S4: Simulated deletion dataset. See Supplementary_Table_S4

Supplementary Table S5: Sensitivity for deletion detection. See Supplementary_Table_S5

Supplementary Table S6: Specificity for deletion detection. See Supplementary_Table_S6

Supplementary Table S7: Simulated insertion dataset. See Supplementary_Table_S7

Supplementary Table S8: Sensitivity for insertion detection. See Supplementary_Table_S8

Supplementary Table S9: Specificity for insertion detection. See Supplementary_Table_S9

Supplementary Table S10: TumorNext solid tumor panel. See Supplementary_Table_S10

Supplementary Table S11: Concordance between TumorNext and hapmap NA07019 Reference. See Supplementary_Table_S11

Supplementary Table S12: Concordance between TumorNext and hapmap NA10857 Reference. See Supplementary_Table_S12

Supplementary Table S13: OncoScan hotspot panel. See Supplementary_Table_S13

Supplementary Table S14: Concordance between TumorNext and OncoScan hotspot panel. See Supplementary_Table_S14

Supplementary Table S15: Concordance between TumorNext and CytoSNP850K.
See Supplementary_Table_S15

Supplementary Table S16: Discordant CytoSNP850K Array SNPs. See Supplementary_Table_S16

Supplementary Table S17: Genes that are actionable based on CNV status

ABL1		CCNE1	CTNNB1	EWSR1	IDH1	MAP2K1	MTOR	PIK3R1	RUNX1
AKT1	BAP1	CDH1	DDR2	FBXW7	IDH2	MAP2k4	MYC	PML	SMAD4
AKT2	BCL2L11	CDK4	DNMT3A	FGF4	IGF1R	MCL1	NF1	PTEN	SMARCA4
ALK	BCR	CDK6	E2F3	FGFR1	JAK2	MDM2	NF2	PTPRD	SOX2
APC	BRAF	CDK8	EGFR	FGFR2	KDM6A	MDM4	NKX2-1	RARA	STK11
AR	BRCA1	CDKN1A	EML4	FGFR3	KDR	MET	NOTCH1	RB1	TET2
ARID1A	BRCA2	CDKN1B	EPHB2	FLT3	KIF5B	MGMT		RET	TP53
ASXL1	CCND1	CDKN2A	ERBB2	FRS2	KIT	MLL	NRAS	RICTOR	TSC1
ATM	CCND2	CDKN2B	ERBB3	HIF1A	KRAS	MPL	PDGFRA	ROS1	TSC2
AURKA	CCND3	CEBPA	ESR1	HRAS	LRP1B	MSH6	PIK3CA	RPTOR	VHL

Supplementary Table S18: Examples of discordant calls between OncoScan and CytoSNP850K

CytoSNP850K Call	OncoScan Call	BAF/logR Review	Notes
CN Gain	CN Loss	CN Loss	22 calls limited to 5 samples – these 5 samples showed identical BAF and LogR plots for all calls on both platforms.
CN Gain	LOH	LOH	20 calls - all calls determined to be LOH
LOH	No Event	No Event	18 calls – CytoSNP850K listed genes on the X chromosome in males as LOH and OncoScan did not list a CNV event. One LOH event on Chromosome 2 was not reported by OncoScan.
CN Gain	No Event	CN Gain and No Event	2 calls – logR and BAF plots matched for both calls. OncoScan did not call a CN Gain for 1 sample and CytoSNP incorrectly called a CN Gain for the other sample.
LOH	CN Gain	CN Gain	5 calls – all calls determined to be CN Gain
LOH	CN Loss	CN Loss	1 call
