# Supplementary material for: TumorNext: A comprehensive tumor profiling assay that incorporates high resolution copy number analysis and germline status to improve testing accuracy
Source: Oncotarget. 2016 Sep 8;7(42):68206–28. doi: 10.18632/oncotarget.11910 (PMC5356550; doi:10.18632/oncotarget.11910)
Supplement: Supplementary file 2 [file oncotarget-07-68206-s002.docx]

| **Supplemental Table 4. Simulated Deletion Dataset** | | | | | | | | | | | | | | | | |
| --- | --- | --- | --- | --- | --- | --- | --- | --- | --- | --- | --- | --- | --- | --- | --- | --- |
|  |  | **Simulated Deletions** | | | | | | | | | | | | | | |
| **Coverage** | **Allele Frequency** | **1bp** | **2bp** | **3bp** | **4bp** | **5bp** | **6bp** | **7bp** | **8bp** | **9bp** | **10bp** | **11-20bp** | **21-30bp** | **31-40bp** | **41-50bp** | **>50bp** |
| **100x** | **Total** | **813** | **346** | **256** | **211** | **168** | **134** | **129** | **107** | **117** | **93** | **604** | **321** | **155** | **82** | **59** |
|  | [0,0.03] | 168 | 51 | 53 | 38 | 36 | 27 | 25 | 23 | 17 | 20 | 123 | 65 | 29 | 16 | 13 |
|  | (0.03,0.05] | 125 | 58 | 36 | 40 | 25 | 26 | 21 | 14 | 15 | 14 | 104 | 57 | 21 | 14 | 16 |
|  | (0.05,0.1] | 123 | 53 | 40 | 31 | 23 | 15 | 16 | 17 | 16 | 6 | 67 | 47 | 28 | 9 | 6 |
|  | (0.1,0.2] | 138 | 64 | 50 | 30 | 23 | 19 | 19 | 19 | 22 | 21 | 101 | 58 | 25 | 18 | 10 |
|  | (0.2,0.3] | 81 | 40 | 24 | 25 | 19 | 12 | 17 | 11 | 12 | 9 | 57 | 36 | 15 | 7 | 5 |
|  | (0.3,0.5] | 105 | 45 | 29 | 24 | 21 | 23 | 18 | 12 | 17 | 13 | 92 | 33 | 24 | 13 | 2 |
|  | (0.5,0.8] | 66 | 32 | 22 | 20 | 19 | 11 | 10 | 8 | 15 | 8 | 54 | 23 | 12 | 4 | 7 |
|  | (0.8,1] | 7 | 3 | 2 | 3 | 2 | 1 | 3 | 3 | 3 | 2 | 6 | 2 | 1 | 1 | 0 |
| **250x** | **Total** | **840** | **352** | **265** | **219** | **172** | **135** | **131** | **107** | **119** | **97** | **624** | **334** | **161** | **84** | **59** |
|  | [0,0.03] | 203 | 76 | 71 | 56 | 46 | 43 | 36 | 26 | 25 | 24 | 167 | 92 | 36 | 24 | 23 |
|  | (0.03,0.05] | 93 | 29 | 23 | 24 | 15 | 12 | 10 | 9 | 10 | 10 | 59 | 33 | 13 | 6 | 6 |
|  | (0.05,0.1] | 133 | 56 | 41 | 31 | 23 | 13 | 17 | 19 | 16 | 10 | 72 | 50 | 32 | 10 | 6 |
|  | (0.1,0.2] | 141 | 65 | 50 | 33 | 25 | 20 | 19 | 18 | 20 | 20 | 113 | 61 | 28 | 17 | 11 |
|  | (0.2,0.3] | 86 | 42 | 25 | 25 | 18 | 12 | 18 | 10 | 12 | 10 | 58 | 40 | 14 | 8 | 4 |
|  | (0.3,0.5] | 105 | 47 | 31 | 26 | 24 | 23 | 18 | 14 | 18 | 13 | 91 | 33 | 25 | 14 | 2 |
|  | (0.5,0.8] | 72 | 34 | 21 | 21 | 19 | 11 | 10 | 8 | 15 | 8 | 56 | 23 | 11 | 4 | 7 |
|  | (0.8,1] | 7 | 3 | 3 | 3 | 2 | 1 | 3 | 3 | 3 | 2 | 8 | 2 | 2 | 1 | 0 |
| **500x** | **Total** | **842** | **352** | **267** | **220** | **173** | **136** | **130** | **108** | **119** | **98** | **630** | **336** | **161** | **84** | **59** |
|  | [0,0.03] | 204 | 76 | 73 | 55 | 47 | 43 | 35 | 26 | 24 | 24 | 167 | 86 | 36 | 24 | 23 |
|  | (0.03,0.05] | 92 | 29 | 23 | 24 | 14 | 13 | 11 | 9 | 10 | 10 | 59 | 38 | 13 | 6 | 6 |
|  | (0.05,0.1] | 133 | 55 | 40 | 33 | 24 | 13 | 17 | 18 | 18 | 10 | 75 | 52 | 32 | 10 | 6 |
|  | (0.1,0.2] | 143 | 62 | 49 | 33 | 25 | 20 | 19 | 20 | 20 | 20 | 117 | 62 | 28 | 17 | 10 |
|  | (0.2,0.3] | 86 | 46 | 27 | 25 | 18 | 12 | 18 | 10 | 11 | 10 | 56 | 39 | 14 | 8 | 5 |
|  | (0.3,0.5] | 107 | 47 | 30 | 26 | 24 | 23 | 18 | 13 | 18 | 13 | 91 | 34 | 25 | 14 | 2 |
|  | (0.5,0.8] | 70 | 34 | 22 | 21 | 19 | 11 | 9 | 9 | 15 | 9 | 58 | 23 | 11 | 4 | 7 |
|  | (0.8,1] | 7 | 3 | 3 | 3 | 2 | 1 | 3 | 3 | 3 | 2 | 7 | 2 | 2 | 1 | 0 |
| **1000x** | **Total** | **847** | **353** | **269** | **222** | **172** | **137** | **133** | **107** | **121** | **98** | **631** | **336** | **162** | **83** | **59** |
|  | [0,0.03] | 202 | 75 | 72 | 55 | 46 | 43 | 35 | 25 | 22 | 24 | 164 | 88 | 35 | 24 | 23 |
|  | (0.03,0.05] | 92 | 31 | 24 | 26 | 16 | 13 | 11 | 10 | 12 | 10 | 63 | 38 | 14 | 6 | 6 |
|  | (0.05,0.1] | 134 | 55 | 40 | 32 | 23 | 13 | 18 | 18 | 19 | 10 | 74 | 51 | 32 | 10 | 6 |
|  | (0.1,0.2] | 146 | 63 | 51 | 32 | 25 | 20 | 19 | 19 | 20 | 19 | 117 | 59 | 28 | 16 | 10 |
|  | (0.2,0.3] | 87 | 44 | 27 | 27 | 18 | 12 | 18 | 11 | 11 | 11 | 56 | 40 | 15 | 9 | 5 |
|  | (0.3,0.5] | 108 | 47 | 30 | 26 | 23 | 24 | 18 | 12 | 19 | 13 | 93 | 35 | 25 | 13 | 2 |
|  | (0.5,0.8] | 71 | 35 | 22 | 21 | 19 | 11 | 11 | 9 | 15 | 9 | 57 | 23 | 11 | 4 | 7 |
|  | (0.8,1] | 7 | 3 | 3 | 3 | 2 | 1 | 3 | 3 | 3 | 2 | 7 | 2 | 2 | 1 | 0 |
|  | **Grand Total** | **3342** | **1403** | **1057** | **872** | **685** | **542** | **523** | **429** | **476** | **386** | **2489** | **1327** | **639** | **333** | **236** |
| Values indicate the number of simulated random test fragments  Note: [0,0.03] = 0% to 3%, (0.03,0.05] = >3% to 5%, (0.05,0.1] = >5 to 10%, etc. | | | | | | | | | | | | | | | | |
