# Supplementary material for: TumorNext: A comprehensive tumor profiling assay that incorporates high resolution copy number analysis and germline status to improve testing accuracy
Source: Oncotarget. 2016 Sep 8;7(42):68206–28. doi: 10.18632/oncotarget.11910 (PMC5356550; doi:10.18632/oncotarget.11910)
Supplement: Supplementary file 3 [file oncotarget-07-68206-s003.docx]

| **Supplemental Table 5. Sensitivity for Deletion Detection** | | | | | | | | | | | | | | | | |
| --- | --- | --- | --- | --- | --- | --- | --- | --- | --- | --- | --- | --- | --- | --- | --- | --- |
| **Coverage** | **Allele Frequency** | **Simulated Deletions** | | | | | | | | | | | | | | |
|  |  | **1bp** | **2bp** | **3bp** | **4bp** | **5bp** | **6bp** | **7bp** | **8bp** | **9bp** | **10bp** | **11-20bp** | **21-30bp** | **31-40bp** | **41-50bp** | **>50bp** |
| 100x | [0,0.03] | 79.76% | 76.47% | 67.92% | 78.95% | 91.67% | 74.07% | 84.00% | 65.22% | 70.59% | 85.00% | 65.04% | 56.92% | 51.72% | 50.00% | 33.33% |
|  | (0.03,0.05] | 90.40% | 84.48% | 86.11% | 75.00% | 80.00% | 69.23% | 61.90% | 92.86% | 93.33% | 71.43% | 70.19% | 47.37% | 42.86% | 57.14% | 60.00% |
|  | (0.05,0.1] | 94.31% | 96.23% | 82.50% | 90.32% | 95.65% | 93.33% | 93.75% | 88.24% | 93.75% | 100% | 92.54% | 91.49% | 96.43% | 88.89% | 50.00% |
|  | (0.1,0.2] | 100% | 100% | 100% | 100% | 100% | 94.74% | 100% | 100% | 95.45% | 100% | 100% | 98.28% | 96.00% | 100% | 100% |
|  | (0.2,0.3] | 100% | 100% | 100% | 100% | 100% | 100% | 100% | 100% | 100% | 100% | 100% | 100% | 100% | 100% | 100% |
|  | (0.3,0.5] | 100% | 100% | 100% | 100% | 100% | 100% | 100% | 100% | 100% | 100% | 100% | 100% | 100% | 100% | 100% |
|  | (0.5,0.8] | 100% | 100% | 100% | 100% | 100% | 100% | 100% | 100% | 100% | 100% | 100% | 100% | 100% | 100% | 100% |
|  | (0.8,1] | 100% | 100% | 100% | 100% | 100% | 100% | 100% | 100% | 100% | 100% | 100% | 100% | 100% | 100% | NA |
| 250x | [0,0.03] | 63.05% | 59.21% | 63.38% | 55.36% | 65.22% | 58.14% | 58.33% | 34.62% | 68.00% | 66.67% | 56.29% | 54.35% | 50.00% | 33.33% | 53.85% |
|  | (0.03,0.05] | 98.92% | 89.66% | 95.65% | 95.83% | 100% | 100% | 80.00% | 88.89% | 90.00% | 100% | 89.83% | 93.94% | 84.62% | 100% | 100% |
|  | (0.05,0.1] | 100% | 100% | 100% | 100% | 100% | 100% | 100% | 100% | 100% | 100% | 100% | 100% | 100% | 100% | 100% |
|  | (0.1,0.2] | 100% | 100% | 100% | 100% | 100% | 100% | 100% | 100% | 100% | 100% | 100% | 100% | 100% | 100% | 100% |
|  | (0.2,0.3] | 100% | 100% | 100% | 100% | 100% | 100% | 100% | 100% | 100% | 100% | 100% | 100% | 100% | 100% | 100% |
|  | (0.3,0.5] | 100% | 100% | 100% | 100% | 100% | 100% | 100% | 100% | 100% | 100% | 100% | 100% | 100% | 100% | 100% |
|  | (0.5,0.8] | 100% | 100% | 100% | 100% | 100% | 100% | 100% | 100% | 100% | 100% | 100% | 100% | 100% | 100% | 100% |
|  | (0.8,1] | 100% | 100% | 100% | 100% | 100% | 100% | 100% | 100% | 100% | 100% | 100% | 100% | 100% | 100% | NA |
| 500x | [0,0.03] | 62.25% | 56.58% | 60.27% | 56.36% | 57.45% | 46.51% | 48.57% | 61.54% | 70.83% | 50.00% | 55.09% | 48.84% | 66.67% | 54.17% | 53.85% |
|  | (0.03,0.05] | 100% | 100% | 100% | 100% | 100% | 100% | 100% | 100% | 100% | 100% | 100% | 97.37% | 100% | 100% | 100% |
|  | (0.05,0.1] | 100% | 100% | 100% | 100% | 100% | 100% | 100% | 100% | 100% | 100% | 100% | 100% | 100% | 100% | 100% |
|  | (0.1,0.2] | 100% | 100% | 100% | 100% | 100% | 100% | 100% | 100% | 100% | 100% | 100% | 100% | 100% | 100% | 100% |
|  | (0.2,0.3] | 100% | 100% | 100% | 100% | 100% | 100% | 100% | 100% | 100% | 100% | 100% | 100% | 100% | 100% | 100% |
|  | (0.3,0.5] | 100% | 100% | 100% | 100% | 100% | 100% | 100% | 100% | 100% | 100% | 100% | 100% | 100% | 100% | 100% |
|  | (0.5,0.8] | 100% | 100% | 100% | 100% | 100% | 100% | 100% | 100% | 100% | 100% | 100% | 100% | 100% | 100% | 100% |
|  | (0.8,1] | 100% | 100% | 100% | 100% | 100% | 100% | 100% | 100% | 100% | 100% | 100% | 100% | 100% | 100% | NA |
| 1000x | [0,0.03] | 70.79% | 65.33% | 73.61% | 65.45% | 65.22% | 62.79% | 60.00% | 72.00% | 72.73% | 66.67% | 62.20% | 55.68% | 60.00% | 54.17% | 69.23% |
|  | (0.03,0.05] | 100% | 100% | 100% | 100% | 100% | 100% | 100% | 100% | 100% | 100% | 100% | 100% | 100% | 100% | 100% |
|  | (0.05,0.1] | 100% | 100% | 100% | 100% | 100% | 100% | 100% | 100% | 100% | 100% | 100% | 100% | 100% | 100% | 100% |
|  | (0.1,0.2] | 100% | 100% | 100% | 100% | 100% | 100% | 100% | 100% | 100% | 100% | 100% | 100% | 100% | 100% | 100% |
|  | (0.2,0.3] | 100% | 100% | 100% | 100% | 100% | 100% | 100% | 100% | 100% | 100% | 100% | 100% | 100% | 100% | 100% |
|  | (0.3,0.5] | 100% | 100% | 100% | 100% | 100% | 100% | 100% | 100% | 100% | 100% | 100% | 100% | 100% | 100% | 100% |
|  | (0.5,0.8] | 100% | 100% | 100% | 100% | 100% | 100% | 100% | 100% | 100% | 100% | 100% | 100% | 100% | 100% | 100% |
|  | (0.8,1] | 100% | 100% | 100% | 100% | 100% | 100% | 100% | 100% | 100% | 100% | 100% | 100% | 100% | 100% | NA |
| Note: [0,0.03] = 0% to 3%, (0.03,0.05] = >3% to 5%, (0.05,0.1] = >5 to 10%, etc. | | | | | | | | | | | | | | | | |
