# Supplementary material for: TumorNext: A comprehensive tumor profiling assay that incorporates high resolution copy number analysis and germline status to improve testing accuracy
Source: Oncotarget. 2016 Sep 8;7(42):68206–28. doi: 10.18632/oncotarget.11910 (PMC5356550; doi:10.18632/oncotarget.11910)
Supplement: Supplementary file 5 [file oncotarget-07-68206-s005.docx]

| **Supplemental Table 7. Simulated Insertion Dataset** | | | | | | | | | | | | | | | | |
| --- | --- | --- | --- | --- | --- | --- | --- | --- | --- | --- | --- | --- | --- | --- | --- | --- |
| **Coverage** | **Allele Frequency** | **Simulated Deletions** | | | | | | | | | | | | | | |
|  |  | **1bp** | **2bp** | **3bp** | **4bp** | **5bp** | **6bp** | **7bp** | **8bp** | **9bp** | **10bp** | **11-20bp** | **21-30bp** | **31-40bp** | **41-50bp** | **>50bp** |
| **100X** | **Total** | **809** | **305** | **239** | **205** | **177** | **158** | **150** | **113** | **105** | **92** | **683** | **330** | **171** | **78** | **54** |
|  | [0,0.03] | 144 | 56 | 39 | 36 | 33 | 37 | 23 | 18 | 26 | 19 | 128 | 71 | 34 | 16 | 12 |
|  | (0.03,0.05] | 129 | 56 | 40 | 28 | 30 | 21 | 25 | 17 | 15 | 19 | 122 | 56 | 25 | 13 | 7 |
|  | (0.05,0.1] | 134 | 38 | 35 | 28 | 27 | 17 | 21 | 12 | 12 | 13 | 98 | 46 | 23 | 10 | 8 |
|  | (0.1,0.2] | 161 | 60 | 41 | 38 | 25 | 31 | 29 | 20 | 15 | 15 | 125 | 53 | 38 | 15 | 13 |
|  | (0.2,0.3] | 86 | 26 | 28 | 24 | 29 | 17 | 11 | 20 | 11 | 11 | 68 | 34 | 17 | 7 | 4 |
|  | (0.3,0.5] | 87 | 32 | 31 | 28 | 19 | 20 | 23 | 18 | 11 | 11 | 71 | 41 | 19 | 11 | 5 |
|  | (0.5,0.8] | 59 | 31 | 21 | 20 | 12 | 14 | 15 | 8 | 12 | 4 | 58 | 24 | 9 | 5 | 5 |
|  | (0.8,1] | 9 | 6 | 4 | 3 | 2 | 1 | 3 | 0 | 3 | 0 | 13 | 5 | 6 | 1 | 0 |
| **250X** | **Total** | **836** | **310** | **240** | **211** | **180** | **161** | **152** | **119** | **110** | **97** | **696** | **332** | **176** | **79** | **55** |
|  | [0,0.03] | 202 | 83 | 56 | 48 | 48 | 44 | 27 | 30 | 34 | 31 | 179 | 103 | 42 | 21 | 13 |
|  | (0.03,0.05] | 71 | 28 | 23 | 19 | 17 | 17 | 19 | 6 | 10 | 9 | 75 | 24 | 18 | 7 | 7 |
|  | (0.05,0.1] | 138 | 40 | 33 | 31 | 27 | 17 | 25 | 15 | 13 | 14 | 93 | 44 | 26 | 10 | 9 |
|  | (0.1,0.2] | 178 | 62 | 46 | 38 | 26 | 31 | 29 | 21 | 15 | 18 | 132 | 54 | 39 | 17 | 12 |
|  | (0.2,0.3] | 83 | 26 | 26 | 23 | 28 | 17 | 12 | 19 | 12 | 9 | 70 | 36 | 19 | 6 | 4 |
|  | (0.3,0.5] | 94 | 33 | 32 | 27 | 20 | 20 | 23 | 19 | 10 | 12 | 76 | 43 | 17 | 12 | 5 |
|  | (0.5,0.8] | 60 | 32 | 21 | 22 | 12 | 14 | 13 | 9 | 13 | 4 | 58 | 21 | 9 | 5 | 5 |
|  | (0.8,1] | 10 | 6 | 3 | 3 | 2 | 1 | 4 | 0 | 3 | 0 | 13 | 7 | 6 | 1 | 0 |
| **500X** | **Total** | **834** | **313** | **244** | **211** | **180** | **163** | **152** | **120** | **111** | **97** | **699** | **333** | **176** | **79** | **55** |
|  | [0,0.03] | 201 | 85 | 54 | 46 | 47 | 44 | 27 | 31 | 30 | 29 | 175 | 101 | 41 | 21 | 12 |
|  | (0.03,0.05] | 72 | 28 | 24 | 22 | 16 | 16 | 14 | 6 | 14 | 9 | 81 | 26 | 19 | 7 | 8 |
|  | (0.05,0.1] | 138 | 41 | 35 | 30 | 29 | 18 | 30 | 15 | 14 | 16 | 95 | 44 | 25 | 10 | 8 |
|  | (0.1,0.2] | 177 | 62 | 48 | 38 | 26 | 33 | 29 | 21 | 15 | 18 | 132 | 54 | 39 | 17 | 13 |
|  | (0.2,0.3] | 83 | 26 | 27 | 22 | 29 | 17 | 12 | 19 | 12 | 9 | 67 | 36 | 19 | 6 | 4 |
|  | (0.3,0.5] | 94 | 33 | 31 | 28 | 19 | 20 | 23 | 19 | 10 | 12 | 77 | 44 | 18 | 12 | 5 |
|  | (0.5,0.8] | 60 | 32 | 21 | 22 | 12 | 14 | 13 | 9 | 13 | 4 | 59 | 21 | 9 | 5 | 5 |
|  | (0.8,1] | 9 | 6 | 4 | 3 | 2 | 1 | 4 | 0 | 3 | 0 | 13 | 7 | 6 | 1 | 0 |
| **1000X** | **Total** | **838** | **312** | **242** | **210** | **180** | **161** | **153** | **121** | **111** | **99** | **708** | **333** | **178** | **79** | **55** |
|  | [0,0.03] | 200 | 84 | 54 | 46 | 46 | 43 | 26 | 30 | 30 | 31 | 175 | 99 | 44 | 21 | 11 |
|  | (0.03,0.05] | 72 | 29 | 24 | 21 | 17 | 17 | 16 | 6 | 14 | 9 | 79 | 28 | 19 | 6 | 9 |
|  | (0.05,0.1] | 141 | 40 | 34 | 30 | 29 | 16 | 30 | 17 | 14 | 16 | 99 | 42 | 23 | 11 | 8 |
|  | (0.1,0.2] | 177 | 62 | 47 | 38 | 26 | 33 | 29 | 21 | 15 | 17 | 130 | 56 | 40 | 17 | 13 |
|  | (0.2,0.3] | 83 | 26 | 26 | 22 | 29 | 17 | 12 | 19 | 12 | 10 | 73 | 36 | 18 | 6 | 4 |
|  | (0.3,0.5] | 94 | 33 | 32 | 28 | 19 | 20 | 22 | 19 | 10 | 12 | 78 | 43 | 19 | 12 | 5 |
|  | (0.5,0.8] | 62 | 32 | 22 | 22 | 12 | 14 | 14 | 9 | 13 | 4 | 60 | 23 | 9 | 5 | 5 |
|  | (0.8,1] | 9 | 6 | 3 | 3 | 2 | 1 | 4 | 0 | 3 | 0 | 14 | 6 | 6 | 1 | 0 |
|  | **Grand Total** | **3317** | **1240** | **965** | **837** | **717** | **643** | **607** | **473** | **437** | **385** | **2786** | **1328** | **701** | **315** | **219** |
| Values indicate the number of simulated random test fragments  Note: [0,0.03] = 0% to 3%, (0.03,0.05] = >3% to 5%, (0.05,0.1] = >5 to 10%, etc. | | | | | | | | | | | | | | | | |
