# Supplementary material for: TumorNext: A comprehensive tumor profiling assay that incorporates high resolution copy number analysis and germline status to improve testing accuracy
Source: Oncotarget. 2016 Sep 8;7(42):68206–28. doi: 10.18632/oncotarget.11910 (PMC5356550; doi:10.18632/oncotarget.11910)
Supplement: Supplementary file 6 [file oncotarget-07-68206-s006.docx]

| **Supplemental Table 8. Sensitivity for Insertion Detection** | | | | | | | | | | | | | | | | |
| --- | --- | --- | --- | --- | --- | --- | --- | --- | --- | --- | --- | --- | --- | --- | --- | --- |
| **Coverage** | **Allele Frequency** | **Simulated Deletions** | | | | | | | | | | | | | | |
|  |  | **1bp** | **2bp** | **3bp** | **4bp** | **5bp** | **6bp** | **7bp** | **8bp** | **9bp** | **10bp** | **11-20bp** | **21-30bp** | **31-40bp** | **41-50bp** | **>50bp** |
| 100x | [0,0.03] | 76.39% | 73.21% | 74.36% | 72.22% | 72.73% | 59.46% | 73.91% | 61.11% | 69.23% | 68.42% | 64.06% | 40.85% | 17.65% | 25.00% | 20.00% |
|  | (0.03,0.05] | 85.27% | 80.36% | 90.00% | 82.14% | 70.00% | 71.43% | 88.00% | 82.35% | 66.67% | 47.37% | 63.11% | 51.79% | 44.00% | 7.69% | 0.00% |
|  | (0.05,0.1] | 92.54% | 100% | 88.57% | 85.71% | 96.30% | 100% | 85.71% | 100% | 83.33% | 92.31% | 94.90% | 84.78% | 78.26% | 90.00% | 33.33% |
|  | (0.1,0.2] | 99.38% | 100% | 100% | 100% | 100% | 100% | 100% | 100% | 100% | 100% | 97.60% | 100% | 89.47% | 93.33% | 100% |
|  | (0.2,0.3] | 100% | 100% | 100% | 100% | 100% | 100% | 100% | 100% | 100% | 100% | 100% | 100% | 100% | 100% | 100% |
|  | (0.3,0.5] | 100% | 100% | 100% | 100% | 100% | 100% | 100% | 100% | 100% | 100% | 100% | 100% | 100% | 100% | 100% |
|  | (0.5,0.8] | 100% | 100% | 100% | 100% | 100% | 100% | 100% | 100% | 100% | 100% | 100% | 100% | 100% | 100% | 100% |
|  | (0.8,1] | 100% | 100% | 100% | 100% | 100% | 100% | 100% | NA | 100% | NA | 100% | 100% | 100% | 100% | NA |
| 250x | [0,0.03] | 58.42% | 61.45% | 53.57% | 64.58% | 52.08% | 68.18% | 48.15% | 66.67% | 52.94% | 58.06% | 44.69% | 24.27% | 23.81% | 23.81% | 7.69% |
|  | (0.03,0.05] | 95.77% | 96.43% | 95.65% | 94.74% | 100% | 94.12% | 94.74% | 100% | 80.00% | 77.78% | 89.33% | 83.33% | 83.33% | 71.43% | 14.29% |
|  | (0.05,0.1] | 100% | 100% | 96.97% | 100% | 100% | 100% | 100% | 100% | 100% | 100% | 100% | 93.18% | 96.15% | 60.00% | 100% |
|  | (0.1,0.2] | 100% | 100% | 100% | 100% | 100% | 100% | 100% | 100% | 100% | 100% | 100% | 100% | 100% | 100% | 100% |
|  | (0.2,0.3] | 100% | 100% | 100% | 100% | 100% | 100% | 100% | 100% | 100% | 100% | 100% | 100% | 100% | 100% | 100% |
|  | (0.3,0.5] | 100% | 100% | 100% | 100% | 100% | 100% | 100% | 100% | 100% | 100% | 100% | 100% | 100% | 100% | 100% |
|  | (0.5,0.8] | 100% | 100% | 100% | 100% | 100% | 100% | 100% | 100% | 100% | 100% | 100% | 100% | 100% | 100% | 100% |
|  | (0.8,1] | 100% | 100% | 100% | 100% | 100% | 100% | 100% | NA | 100% | NA | 100% | 100% | 100% | 100% | NA |
| 500x | [0,0.03] | 57.21% | 58.82% | 72.22% | 60.87% | 48.94% | 61.36% | 40.74% | 70.97% | 60.00% | 62.07% | 44.00% | 29.70% | 34.15% | 19.05% | 0.00% |
|  | (0.03,0.05] | 100% | 100% | 100% | 100% | 100% | 100% | 100% | 83.33% | 100% | 100% | 97.53% | 100% | 78.95% | 57.14% | 66.67% |
|  | (0.05,0.1] | 100% | 100% | 100% | 100% | 100% | 100% | 100% | 100% | 100% | 100% | 100% | 100% | 100% | 100% | 100% |
|  | (0.1,0.2] | 100% | 100% | 100% | 100% | 100% | 100% | 100% | 100% | 100% | 100% | 100% | 100% | 100% | 100% | 100% |
|  | (0.2,0.3] | 100% | 100% | 100% | 100% | 100% | 100% | 100% | 100% | 100% | 100% | 100% | 100% | 100% | 100% | 100% |
|  | (0.3,0.5] | 100% | 100% | 100% | 100% | 100% | 100% | 100% | 100% | 100% | 100% | 100% | 100% | 100% | 100% | 100% |
|  | (0.5,0.8] | 100% | 100% | 100% | 100% | 100% | 100% | 100% | 100% | 100% | 100% | 100% | 100% | 100% | 100% | 100% |
|  | (0.8,1] | 100% | 100% | 100% | 100% | 100% | 100% | 100% | NA | 100% | NA | 100% | 100% | 100% | 100% | NA |
| 1000x | [0,0.03] | 62.00% | 61.90% | 75.93% | 69.57% | 56.52% | 74.42% | 46.15% | 73.33% | 70.00% | 67.74% | 53.71% | 41.41% | 22.73% | 19.05% | 0.00% |
|  | (0.03,0.05] | 100% | 100% | 100% | 100% | 100% | 100% | 100% | 100% | 100% | 100% | 100% | 100% | 89.47% | 66.67% | 100% |
|  | (0.05,0.1] | 100% | 100% | 100% | 100% | 100% | 100% | 100% | 100% | 100% | 100% | 100% | 100% | 100% | 100% | 100% |
|  | (0.1,0.2] | 100% | 100% | 100% | 100% | 100% | 100% | 100% | 100% | 100% | 100% | 100% | 100% | 100% | 100% | 100% |
|  | (0.2,0.3] | 100% | 100% | 100% | 100% | 100% | 100% | 100% | 100% | 100% | 100% | 100% | 100% | 100% | 100% | 100% |
|  | (0.3,0.5] | 100% | 100% | 100% | 100% | 100% | 100% | 100% | 100% | 100% | 100% | 100% | 100% | 100% | 100% | 100% |
|  | (0.5,0.8] | 100% | 100% | 100% | 100% | 100% | 100% | 100% | 100% | 100% | 100% | 100% | 100% | 100% | 100% | 100% |
|  | (0.8,1] | 100% | 100% | 100% | 100% | 100% | 100% | 100% | NA | 100% | NA | 100% | 100% | 100% | 100% | NA |
| Note: [0,0.03] = 0% to 3%, (0.03,0.05] = >3% to 5%, (0.05,0.1] = >5 to 10%, etc. | | | | | | | | | | | | | | | | |
