# Supplementary material for: TumorNext: A comprehensive tumor profiling assay that incorporates high resolution copy number analysis and germline status to improve testing accuracy
Source: Oncotarget. 2016 Sep 8;7(42):68206–28. doi: 10.18632/oncotarget.11910 (PMC5356550; doi:10.18632/oncotarget.11910)
Supplement: Supplementary file 8 [file oncotarget-07-68206-s008.docx]

| **Supplemental Table 10. TumorNext Solid Tumor Panel** | | | | | | | | | |
| --- | --- | --- | --- | --- | --- | --- | --- | --- | --- |
| Genes with full exon coverage | | | | | | | | | |
| ABL1 | BCL2L1 | CSF1R | FANCA | GNAQ | MAP2K2 | MYD88 | PDGFRB | RB1 | TET2 |
| ABL2* | BCL2L2 | CTNNB1 | FANCC | GNAS | MAP2K4 | NBN | PIK3CA | RET | TMEM127 |
| AKT1 | BMPR1A | DDR2 | FANCD2 | HRAS | MAX | NF1 | PIK3CB | ROS1 | TOP1 |
| AKT2 | BRAF | DNMT3A | FBXW7 | HSP90AA1 | MDM2 | NF2 | PIK3CG | RUNX1 | TP53 |
| AKT3 | BRCA1 | EGFR | FGFR1 | IDH1 | MET | NOTCH1 | PIK3R1 | SDHA | TSC1 |
| ALK | BRCA2 | EPHA2 | FGFR2 | IDH2 | MITF | NOTCH2 | PIK3R2** | SDHAF2 | TSC2 |
| APC | BRIP1 | EPHA3 | FGFR3 | IGF1R | MLH1 | NOTCH3 | PMS2*** | SDHB | VHL |
| AR | BTK | EPHA5 | FGFR4 | IL7R | MLL | NOTCH4 | PTCH1 | SDHC |  |
| ARAF | CBL | EPHA7 | FH | JAK1 | MPL | NRAS | PTEN | SDHD |  |
| ATM | CCND1 | EPHB1 | FLCN | JAK2 | MRE11A | NTRK1 | PTPN11 | SMAD4 |  |
| AURKA | CCND3 | ERBB2 | FLT1 | JAK3 | MSH2 | PAK7 | RAD50 | SMARCB1 |  |
| AURKB | CDH1 | ERBB3 | FLT3 | KDR | MSH6 | PALB2 | RAD51C | SMO |  |
| AXL | CDK4 | ERBB4 | FLT4 | KIT | MTOR | PARP1 | RAD51D | SRC |  |
| BARD1 | CDKN2A | ESR1 | FOXL2 | KRAS | MUTYH | PARP2 | RAF1 | STAT3 |  |
| BCL2 | CHEK2 | EZH2 | GNA11 | MAP2K1 | MYCN | PDGFRA | RARA | STK11 |  |
| Genes analyzed for stuctural variants | | | | | | | | | |
| ALK | BRAF | BRCA2 | FGFR2 | KIT | NTRK1 | RAF1 | ROS1 |  |  |
| BCL2 | BRCA1 | FGFR1 | FGFR3 | NOTCH2 | PDGFRA | RET |  |  |  |
| *ABL2 exon 2 is not included in the assay  **PIK3R2 exon 13 is not included in the assay  ***Only *PMS2* exons 1-10 are analyzed | | | | | | | | | |
