# Supplementary material for: TumorNext: A comprehensive tumor profiling assay that incorporates high resolution copy number analysis and germline status to improve testing accuracy
Source: Oncotarget. 2016 Sep 8;7(42):68206–28. doi: 10.18632/oncotarget.11910 (PMC5356550; doi:10.18632/oncotarget.11910)
Supplement: Supplementary file 9 [file oncotarget-07-68206-s009.docx]

| **Supplemental Table 11. Concordance between TumorNext and HapMap NA07019 Reference** | | | | | | |
| --- | --- | --- | --- | --- | --- | --- |
| **HapMap SNP** | **Gene** | **Variant** | **Hapmap Alleles** | **NA07019 Reference Genotype** | **TumorNext Result** | **Concordant?** |
| rs6685892 | NOTCH2 | c.7341T>A | A/T | AT | TT | N* |
| rs3899528 | NOTCH2 | c.680C>A | G/T | GG | GT | N* |
| rs2298258 | DDR2 | c.1260C>G | C/G | CG | CG | Y |
| rs2746462 | SDHB | c.18C>A | . | TT | TT | Y |
| rs1805415 | PARP1 | c.1056A>G | C/T | CT | CT | Y |
| rs3219489 | MUTYH | c.1014G>C | C/G | CG | CG | Y |
| rs1800861 | RET | c.2307G>T | . | GT | GT | Y |
| rs2071702 | MLL | c.7254C>T | C/T | CT | CT | Y |
| rs7993418 | FLT1 | c.3639C>T | A/G | AA | AA | Y |
| rs206076 | BRCA2 | c.6513G>C | C/G | CC | CC | Y |
| rs169547 | BRCA2 | c.7397T>C | C/T | CC | CC | Y |
| rs1058808 | ERBB2 | c.3508C>G | C/G | CG | CG | Y |
| rs1799949 | BRCA1 | c.2082C>T | A/G | AG | AG | Y |
| rs1042522 | TP53 | c.215C>G | C/G | CG | CG | Y |
| rs1548555 | NOTCH3 | c.5362+3T>C | A/G | GG | GG | Y |
| rs273269 | PIK3R2 | c.1911T>C | C/T | CC | CC | Y |
| rs10250 | MAP2K2 | c.660C>A | G/T | GT | GT | Y |
| rs2070094 | BARD1 | c.1519G>A | C/T | CT | CT | Y |
| rs2070093 | BARD1 | c.1518T>C | A/G | GG | GG | Y |
| rs2229571 | BARD1 | c.1134G>C | C/G | CG | CG | Y |
| rs2070096 | BARD1 | c.1053G>C | C/G | CG | CG | Y |
| rs1048108 | BARD1 | c.70C>T | A/G | AG | AG | Y |
| rs1670283 | ALK | c.4381A>G | C/T | CC | CC | Y |
| rs2293564 | ALK | c.1500A>G | C/T | CC | CC | Y |
| rs2246745 | ALK | c.702T>A | A/T | AT | AT | Y |
| rs1047972 | AURKA | c.169A>G | C/T | CC | CC | Y |
| rs7688609 | FGFR3 | c.1953G>A | A/G | AA | AA | Y |
| rs1870377 | KDR | c.1416A>T | A/T | AT | AT | Y |
| rs67622085 | APC | c.4326T>A | . | TA | TA | Y |
| rs246388 | PDGFRB | c.3252A>G | C/T | CC | CC | Y |
| rs351855 | FGFR4 | c.1162G>A | A/G | AG | AG | Y |
| rs1126417 | SDHA | c.891T>C | . | CC | CC | Y |
| rs619203 | ROS1 | c.6686C>G | C/G | GG | GC | N* |
| rs2243378 | ROS1 | c.303A>T | A/T | AA | AA | Y |
| rs4986934 | ESR1 | c.729T>C | C/T | CC | CC | Y |
| rs423023 | NOTCH4 | c.1044C>G | C/G | CG | CG | Y |
| rs1051130 | CCND3 | c.775T>G | A/C | AC | AC | Y |
| rs345730 | EPHA7 | c.2076G>A | C/T | TT | TT | Y |
| rs849389 | PIK3CG | c.972A>G | A/G | GG | GG | Y |
| rs2230460 | PIK3CG | c.2850C>T | C/T | CT | CT | Y |
| rs2228617 | SMO | c.1164G>C | C/G | CG | CG | Y |
| rs1050171 | EGFR | c.2361G>A | A/G | AG | AG | Y |
| rs4489420 | NOTCH1 | c.312T>C | A/G | GG | GG | Y |
| *Note: For HapMap NA07019, the reference genotypes for rs6685892, rs3899528 and rs619203 were initially discordant, but all were Sanger sequenced and found to be concordant with the NGS calls. | | | | | | |
