# Supplementary material for: TumorNext: A comprehensive tumor profiling assay that incorporates high resolution copy number analysis and germline status to improve testing accuracy
Source: Oncotarget. 2016 Sep 8;7(42):68206–28. doi: 10.18632/oncotarget.11910 (PMC5356550; doi:10.18632/oncotarget.11910)
Supplement: Supplementary file 10 [file oncotarget-07-68206-s010.docx]

| **Supplemental Table 12. Concordance between TumorNext and HapMap NA10857 Reference** | | | | | | |
| --- | --- | --- | --- | --- | --- | --- |
| **HapMap SNP** | **Gene** | **Variant** | **Hapmap Alleles** | **NA10857 Reference Genotype** | **TumorNext Result** | **Concordant?** |
| rs6685892 | NOTCH2 | c.7341T>A | A/T | AT | AT | Y |
| rs2746462 | SDHB | c.18C>A | . | TT | TT | Y |
| rs1805415 | PARP1 | c.1056A>G | C/T | CC | CC | Y |
| rs3219489 | MUTYH | c.1014G>C | C/G | CG | CG | Y |
| rs3737139 | JAK1 | c.2097C>G | C/G | CG | CG | Y |
| rs1800861 | RET | c.2307G>T | . | GT | GT | Y |
| rs1800863 | RET | c.2712C>G | C/G | CG | CG | Y |
| rs17537350 | FLT1 | c.1704G>A | C/T | CT | CT | Y |
| rs28897727 | BRCA2 | c.4258G>T | . | GT | GT | Y |
| rs206076 | BRCA2 | c.6513G>C | C/G | CC | CC | Y |
| rs1799955 | BRCA2 | c.7242A>G | A/G | AG | AG | Y |
| rs169547 | BRCA2 | c.7397T>C | C/T | CC | CC | Y |
| rs2231301 | BCL2L2 | c.123G>A | A/G | AG | AG | Y |
| rs1748 | TSC2 | c.5202T>C | C/T | CT | CT | Y |
| rs1051771 | TSC2 | c.5397G>C | C/G | CG | CG | Y |
| rs11649210 | FANCA | c.3807G>C | C/G | CG | CG | Y |
| rs2285892 | NF1 | c.2034G>A | A/G | AG | AG | Y |
| rs56013763 | NF1 | c.7045C>T | . | CT | CT | Y |
| rs9901455 | RAD51D | c.234C>T | . | GA | GA | Y |
| rs1058808 | ERBB2 | c.3508C>G | C/G | GG | GG | Y |
| rs1799949 | BRCA1 | c.2082C>T | A/G | GA | GA | Y |
| rs1548555 | NOTCH3 | c.5362+3T>C | A/G | GG | GG | Y |
| rs273269 | PIK3R2 | c.1911T>C | C/T | CC | CC | Y |
| rs4900 | GNA11 | c.771C>T | C/T | TT | TT | Y |
| rs10250 | MAP2K2 | c.660C>A | G/T | TT | TT | Y |
| rs2070094 | BARD1 | c.1519G>A | C/T | CT | CT | Y |
| rs2070093 | BARD1 | c.1518T>C | A/G | GG | GG | Y |
| rs2229571 | BARD1 | c.1134G>C | C/G | CG | CG | Y |
| rs1048108 | BARD1 | c.70C>T | A/G | AG | AG | Y |
| rs1881421 | ALK | c.4587C>G | C/G | CC | CC | Y |
| rs1670283 | ALK | c.4381A>G | C/T | CC | CC | Y |
| rs2293564 | ALK | c.1500A>G | C/T | CC | CC | Y |
| rs2246745 | ALK | c.702T>A | A/T | AT | AT | Y |
| rs1800937 | MSH6 | c.642C>T | C/T | CT | CT | Y |
| rs1047972 | AURKA | c.169A>G | C/T | CC | CC | Y |
| rs7688609 | FGFR3 | c.1953G>A | A/G | AA | AA | Y |
| rs1870377 | KDR | c.1416A>T | A/T | AA | AA | Y |
| rs246388 | PDGFRB | c.3252A>G | C/T | CT | CT | Y |
| rs2228439 | PDGFRB | c.1149G>C | C/G | CG | CG | Y |
| rs351855 | FGFR4 | c.1162G>A | A/G | AG | AG | Y |
| rs1126417 | SDHA | c.891T>C | . | TC | TC | Y |
| rs2243378 | ROS1 | c.303A>T | A/T | AT | AT | Y |
| rs4986934 | ESR1 | c.729T>C | C/T | CC | CC | Y |
| rs1051130 | CCND3 | c.775T>G | A/C | CC | CC | Y |
| rs345730 | EPHA7 | c.2076G>A | C/T | CT | CT | Y |
| rs35775721 | MET | c.534C>T | . | CT | CT | Y |
| rs33917957 | MET | c.1124A>G | . | AG | AG | Y |
| rs2228617 | SMO | c.1164G>C | C/G | CG | CG | Y |
| rs1050171 | EGFR | c.2361G>A | A/G | AG | AG | Y |
| rs1805319 | PMS2 | c.780C>G | C/G | CC | CC | Y |
| rs357564 | PTCH1 | c.3944C>T | A/G | AG | AG | Y |
