# Supplementary material for: TumorNext: A comprehensive tumor profiling assay that incorporates high resolution copy number analysis and germline status to improve testing accuracy
Source: Oncotarget. 2016 Sep 8;7(42):68206–28. doi: 10.18632/oncotarget.11910 (PMC5356550; doi:10.18632/oncotarget.11910)
Supplement: Supplementary file 11 [file oncotarget-07-68206-s011.docx]

| **Supplemental Table 13. OncoScan HotSpot Panel** | | |
| --- | --- | --- |
| Gene | Target Mutation | Percent mutant detectable |
| BRAF | BRAFp.V600Kc.1798_1799GT>AA | 20% |
|  | BRAFp.V600Ec.1799T>A | 20% |
|  | BRAFp.G469Ac.1406G>C | 20% |
|  | BRAFp.G469Ec.1406G>A | 30%* |
| EGFR | EGFRp.G719Cc.2155G>T | 20% |
|  | EGFRp.G719Sc.2155G>A | 30% |
|  | EGFRp.G719Ac.2156G>C | 20% |
|  | EGFRp.E746_A750delc.2235_2249del15 | 20% |
|  | EGFRp.E746_A750delc.2236_2250del15 | 30%* |
|  | EGFRp.E746_T751>Ac.2237_2251del15 | 30%* |
|  | EGFRp.L747_A750>Pc.2239_2248TTAAGAGAAG>C | 30%* |
|  | EGFRp.L747_E749delc.2239_2247del9 | 30%* |
|  | EGFRp.L747_T751delc.2240_2254del15 | 20% |
|  | EGFRp.L747_P753>Sc.2240_2257del18 | 20% |
|  | EGFRp.V769_D770insASVc.2307_2308ins9 | 20% |
|  | EGFRp.D770_N771insSVDc.2311_2312ins9 | 20% |
|  | EGFRp.H773_V774insNPHc.2319_2320ins9 | 20% |
|  | EGFRp.T790Mc.2369C>T | 20% |
|  | EGFRp.L858Rc.2573T>G | 30%* |
|  | EGFRp.L861Qc.2582T>A | 30% |
| IDH1 | IDH1p.R132Hc.395G>A | 30% |
| IDH2 | IDH2p.R140Qc.419G>A | 20% |
|  | IDH2p.R172Kc.515G>A | 20% |
| KRAS | KRASp.A146Pc.436G>C | 20% |
|  | KRASp.Q61Hc.183A>C | 20% |
|  | KRASp.Q61Hc.183A>T | 20% |
|  | KRASp.Q61Kc.180_181TC>AA | 30% |
|  | KRASp.Q61Kc.181C>A | 30% |
|  | KRASp.G13Dc.38G>A | 20% |
|  | KRASp.G12Ac.35G>C | 20% |
|  | KRASp.G12Dc.35G>A | 30% |
|  | KRASp.G12Vc.35G>T | 30%* |
|  | KRASp.G12Cc.34G>T | 20% |
|  | KRASp.G12Sc.34G>A | 30% |
| NRAS | NRASp.Q61Lc.182A>T | 20% |
|  | NRASp.Q61Rc.182A>G | 20% |
|  | NRASp.Q61Kc.181C>A | 30%* |
|  | NRASp.G12Dc.35G>A | 20% |
|  | NRASp.G12Vc.35G>T | 20% |
|  | NRASp.G12Cc.34G>T | 30%* |
|  | NRASp.G12Sc.34G>A | 30%* |
| PIK3CA | PIK3CAp.E542Kc.1624G>A | 20% |
|  | PIK3CAp.E545Kc.1633G>A | 30%* |
|  | PIK3CAp.Q546Kc.1636C>A | 30% |
|  | PIK3CAp.H1047Lc.3140A>T | 30% |
|  | PIK3CAp.H1047Rc.3140A>G | 20% |
| PTEN | PTENp.R130*c.388C>T | 20% |
|  | PTENp.R130Gc.388C>G | 20% |
|  | PTENp.R130fs*4c.389delG | 20% |
|  | PTENp.R130Qc.389G>A | 30% |
|  | PTENp.R159Sc.477G>T | 30% |
|  | PTENp.R233*c.697C>T | 30% |
|  | PTENp.P248fs*5c.741_742insA | 30%* |
|  | PTENp.K267fs*9c.800delA | 20% |
| TP53 | TP53p.R306*c.916C>T | 20% |
|  | TP53p.R282Wc.844C>T | 30%* |
|  | TP53p.R273Hc.818G>A | 20% |
|  | TP53p.R273Lc.818G>T | 20% |
|  | TP53p.R273Cc.817C>T | 30% |
|  | TP53p.R273Sc.817C>A | 30% |
|  | TP53p.R249Sc.747G>T | 20% |
|  | TP53p.R248Lc.743G>T | 20% |
|  | TP53p.R248Qc.743G>A | 20% |
|  | TP53p.R248Wc.742C>T | 30%* |
|  | TP53p.G245Cc.733G>T | 30% |
|  | TP53p.G245Sc.733G>A | 20% |
|  | TP53p.Y220Cc.659A>G | 20% |
|  | TP53p.R213*c.637C>T | 20% |
|  | TP53p.R196*c.586C>T | 20% |
|  | TP53p.H179Rc.536A>G | 20% |
|  | TP53p.C176Fc.527G>T | 20% |
|  | TP53p.R175Hc.524G>A | 20% |
|  | TP53p.Y163Cc | 20% |
|  | TP53p.V157Fc.469G>T | 20% |
| Table provided by Affymetrix  * 30% at slightly lower specificity (95% as compared to 99.9% as determined by spike in experiments) | | |
