# Supplementary material for: TumorNext: A comprehensive tumor profiling assay that incorporates high resolution copy number analysis and germline status to improve testing accuracy
Source: Oncotarget. 2016 Sep 8;7(42):68206–28. doi: 10.18632/oncotarget.11910 (PMC5356550; doi:10.18632/oncotarget.11910)
Supplement: Supplementary file 12 [file oncotarget-07-68206-s012.docx]

| **Supplemental Table 14. Concordance between TumorNext and OncoScan hotspot panel** | | |
| --- | --- | --- |
| **Sample** | **Mutation** | **Confirmed on TumorNext** |
| RD_005 | NRAS:p.G12D:c.35G>A | Yes |
|  | PIK3CA:p.E545K:c.1633G>A | Yes |
| RD_006 | KRAS:p.G12D:c.35G>A | Yes |
| RD_009 | TP53:p.R282W:c.844C>T | Yes |
| 10-SU4610-B2 | KRAS:p.G12C:c.34G>T | Yes |
| 1008712-139858 | PIK3CA:p.E542K:c.1624G>A | Yes |
| 1009558 | TP53:p.R306*:c.916C>T | Yes |
| 1009643-249766 | TP53:p.R213*:c.637C>T | Yes |
| 1009953-256930 | TP53:p.R175H:c.524G>A | Yes |
| 1011952-163362 | TP53:p.R248Q/L:c.743G>A/T | Yes |
| 1012372-348183 | KRAS:p.G13D:c.38G>A | Yes |
| 1012408-346144 | PIK3CA:p.E545K:c.1633G>A | Yes |
| 1014032-395561 | TP53:p.R175H:c.524G>A | Yes |
| 1014577-406010 | KRAS:p.G12D/V:c.35G>A/T | Yes |
| 3000629-336003 | PIK3CA:p.E545K:c.1633G>A | Yes |
| 3001006-273598 | EGFR:p.E746_A750del:c.2236_2250del15 | Yes |
| BR14-194 | KRAS:p.G12D:c.35G>A | Yes |
|  | TP53:p.R213*:c.637C>T | Yes |
| BR14-239 | KRAS:p.G12V:c.35G>T | Yes |
| BR13-29 | KRAS:p.Q61K:c.180_181TC>TA | Yes |
|  | TP53:p.R273C:c.817C>T | Yes |
| BR14-67 | TP53:p.R248Q:c.743G>A | Yes |
| BR14-88 | NRAS:p.Q61R:c.182A>G | Yes |
| BR14-194 | KRAS:p.G12D:c.35G>A | Yes |
|  | TP53:p.R213*:c.637C>T | Yes |
| BR14-239 | KRAS:p.G12V:c.35G>T | Yes |
| BR13-10 | TP53:p.R248Q:c.743G>A | Yes |
| BR13-139 | TP53:p.R248Q:c.743G>A | Yes |
| BR14-69 | PIK3CA:p.H1047R:c.3140A>G | Yes |
| BR14-83 | PIK3CA:p.H1047R:c.3140A>G | Yes |
|  | TP53:p.R248Q:c.743G>A | Yes |
| BR13-180 | PIK3CA:p.H1047R:c.3140A>G | Yes |
|  | KRAS:p.G12D:c.35G>A | Yes |
| BR14-75 | EGFR:p.L858R:c.2573T>G | Yes |
| BR11-4 | KRAS:p.G12V:c.35G>T | Yes |
| BR13-25 | KRAS:p.G12D:c.35G>A | Yes |
|  | TP53:p.R273H:c.818G>A | Yes |
| BR13-114 | KRAS:p.Q61H:c.183A>C | Yes |
| BR13-184 | KRAS:p.G13D:c.38G>A | Yes |
| BR11-49 | BRAF:p.V600E:c.1799T>A | Yes |
| BR11-50 | BRAF:p.V600E:c.1799T>A | Yes |
| BR12-30 | BRAF:p.V600E:c.1799T>A | Yes |
| BR12-110 | PTEN:p.K267fs*9:c.800delA | Yes |
|  | KRAS:p.G12D:c.35G>A | Yes |
| BR13-162 | TP53:p.R273C:c.817C>T | Yes |
| BR13-163 | KRAS:p.G12V:c.35G>T | Yes |
|  | TP53:p.R273H:c.818G>A | Yes |
| BR14-209 | PIK3CA:p.H1047R:c.3140A>G | Yes |
|  | KRAS:p.G12S:c.34G>A | Yes |
| BR12-15 | KRAS: p.G12D: c.35G>A | Yes |
