# Supplementary material for: TumorNext: A comprehensive tumor profiling assay that incorporates high resolution copy number analysis and germline status to improve testing accuracy
Source: Oncotarget. 2016 Sep 8;7(42):68206–28. doi: 10.18632/oncotarget.11910 (PMC5356550; doi:10.18632/oncotarget.11910)
Supplement: Supplementary file 13 [file oncotarget-07-68206-s013.docx]

| **Supplemental Table 15. Concordance between TumorNext and CytoSNP850K** | | | | | | |
| --- | --- | --- | --- | --- | --- | --- |
| **Sample** | **Total heterozygous calls** | **Concordant calls** | **Disconcordant calls** | **Genes with discordant calls*** | **Discordant call Sanger confirmed?** | **Concordance (%)** |
| 1005934 | 49 | 45 | 4 | DNMT3A  ERBB2  IGF1R(2) | No | 100 |
| 1006138_169167 | 57 | 55 | 2 | IGF1R S606 IGF1R S24 | No | 100 |
| 1006807_182985 | 61 | 58 | 3 | DNMT3A  IGF1R S609 IGF1R S24 | No | 100 |
| 1006866_189171 | 58 | 55 | 3 | DNMT3A  IGF1R(2) | No | 100 |
| 1009698_250607 | 63 | 55 | 8 | CDKN2A  DNMT3A  FLT4  IGF1R  MUTYH  MYCN  NOTCH3  RET | No | 100 |
| 1009896_256371 | 56 | 54 | 2 | DNMT3A  IGF1R | No | 100 |
| 394820_3000641 | 46 | 44 | 2 | DNMT3A  IGF1R | No | 100 |
| 384350_3000642 | 66 | 59 | 7 | DNMT3A  EPHA2  FANCA  FLT4  IGF1R(2)  NOTCH4 | No | 100 |
| 383734_3000643 | 53 | 45 | 8 | DNMT3A  FANCA  FLT4  IGF1R  MUTYH  MYCN  NOTCH3  RET | No | 100 |
| 1009558 | 64 | 58 | 6 | IGF1R(2)  MUTYH  NOTCH4  NTRK1  RET | No | 100 |
| 251424_1009749 | 50 | 48 | 2 | DNMT3A  IGF1R(2) | No | 100 |
| 256930_1009953 | 66 | 60 | 6 | DNMT3A  EPHA5  FANCA  IGF1R  MYCN  RET | No | 100 |
| 292837_1011150 | 63 | 56 | 7 | CDKN2A  DNMT3A  FANCA  IGF1R(2)  MYCN  NOTCH3  RET | No | 100 |
| 384009_1013490 | 65 | 64 | 1 | DNMT3A | No | 100 |
| 402958_1014378 | 61 | 57 | 4 | DNMT3A  IGF1R(2)  MYCN | No | 100 |
| RD_006 | 53 | 51 | 2 | DNMT3A  IGF1R | No | 100 |
| RD_008 | 44 | 42 | 2 | DNMT3A  IGF1R | No | 100 |
| RD_011F1 | 43 | 42 | 1 | DNMT3A | No | 100 |
| BR13_29_03_01 | 42 | 38 | 4 | DNMT3A  IGF1R(3) | No | 100 |
| BR_14_4305_01 | 57 | 55 | 2 | DNMT3A  IGF1R | No | 100 |
| BR_14_19405_01 | 66 | 63 | 3 | DNMT3A  FANCD2  IGF1R | No | 100 |
| 334325_1012045 | 47 | 45 | 2 | DNMT3A  IGF1R | No | 100 |
| 1002837_84444 | 52 | 50 | 2 | DNMT3A  IGF1R | No | 100 |
| 1014577 | 63 | 63 | 3 | DNMT3A  FANCA  IGF1R | No | 100 |
| RD_005A1 | 60 | 57 | 3 | DNMT3A  IGF1R(2) | No | 100 |
| 1012374_346270 | 57 | 53 | 4 | DNMT3A  EPHA2  IGF1R(2) | No | 100 |
| 1008712 | 67 | 64 | 3 | DNMT3A  IGF1R(2) | No | 100 |
| 1010098 | 66 | 64 | 2 | DNMT3A  IGF1R | No | 100 |
| 1011142 | 53 | 51 | 2 | DNMT3A  IGF1R | No | 100 |
| 1013301_375808 | 51 | 49 | 2 | DNMT3A  IGF1R | No | 100 |
| 1014032 | 37 | 35 | 2 | DNMT3A  IGF1R | No | 100 |
| S002_53032B_109436 | 64 | 52 | 12 | CDKN2A  DNMT3A  EPHA2  FANCA  IDH2  IGF1R(2)  MUTYH  MYCN  NOTCH4(2)  RET | No | 100 |
| S005_10601C_154417 | 53 | 50 | 3 | DNMT3A  IGF1R  NOTCH4 | No | 100 |
| S09_259 | 68 | 66 | 2 | DNMT3A  IGF1R | No | 100 |
| SP11_180A14 | 59 | 56 | 3 | DNMT3A  IGF1R  EPHA2 | Yes – EPHA2 SNP confirmed | 98 |
| **Total** | **1,980** | **1,845** | **135** |  |  | **99.9** |
| **Concordance = Number of common calls/ total calls x 100** | | | | |  |  |
| *See supplemental table 16 for discordant SNPs  Note: A number in parentheses next to a gene symbol indicates multiple discordant SNPs in that gene | | | | | | |
