# Supplementary material for: TumorNext: A comprehensive tumor profiling assay that incorporates high resolution copy number analysis and germline status to improve testing accuracy
Source: Oncotarget. 2016 Sep 8;7(42):68206–28. doi: 10.18632/oncotarget.11910 (PMC5356550; doi:10.18632/oncotarget.11910)
Supplement: Supplementary file 14 [file oncotarget-07-68206-s014.docx]

| **Supplemental Table 1. Simulated SNV Dataset** | | | | | | | | |
| --- | --- | --- | --- | --- | --- | --- | --- | --- |
|  | **Allele Frequencies** | | | | | | | |
| **Coverage** | 0 - 3% | 3% - 5% | 5% -10% | 10% - 20% | 20% - 30% | 30% - 50% | 50% - 80% | 80% - 100% |
| **100x** | 4,815 | 8,420 | 6,677 | 6,653 | 6,662 | 6,643 | 6,665 | 6,648 |
| **250x** | 6,796 | 6,794 | 6,787 | 6,764 | 6,777 | 6,780 | 6,757 | 6,817 |
| **500x** | 6,815 | 6,842 | 6,834 | 6,837 | 6,835 | 6,823 | 6,805 | 6,838 |
| **1000x** | 6,866 | 6,828 | 6,864 | 6,852 | 6,875 | 6,871 | 6,863 | 6,855 |
| **Total** | 25,292 | 28,884 | 27,162 | 27,106 | 27,149 | 27,117 | 27,090 | 27,158 |
| Values indicate the number of simulated random test fragments | | | | | | | | |

| **Supplemental Table 2. Simulated SNV Sensitivity** | | | | | | | | |
| --- | --- | --- | --- | --- | --- | --- | --- | --- |
|  | **Allele Frequencies** | | | | | | | |
| **Coverage** | 0 - 3% | 3% - 5% | 5% -10% | 10% - 20% | 20% - 30% | 30% - 50% | 50% - 80% | 80% - 100% |
| **100x** | 80.04% | 88.57% | 99.93% | 100.00% | 100.00% | 100.00% | 100.00% | 100.00% |
| **250x** | 90.38% | 99.26% | 100.00% | 100.00% | 100.00% | 100.00% | 100.00% | 100.00% |
| **500x** | 99.91% | 100.00% | 100.00% | 100.00% | 100.00% | 100.00% | 100.00% | 100.00% |
| **1000x** | 99.97% | 100.00% | 100.00% | 100.00% | 100.00% | 100.00% | 100.00% | 100.00% |

| **Supplemental Table 3. Simulated SNV Specificity** | | | | | | | | |
| --- | --- | --- | --- | --- | --- | --- | --- | --- |
|  | **Allele Frequencies** | | | | | | | |
| **Coverage** | 0 - 3% | 3% - 5% | 5% -10% | 10% - 20% | 20% - 30% | 30% - 50% | 50% - 80% | 80% - 100% |
| **100x** | 100.00% | 100.00% | 100.00% | 100.00% | 100.00% | 100.00% | 100.00% | 100.00% |
| **250x** | 100.00% | 100.00% | 100.00% | 100.00% | 100.00% | 100.00% | 100.00% | 100.00% |
| **500x** | 100.00% | 100.00% | 100.00% | 100.00% | 100.00% | 100.00% | 100.00% | 100.00% |
| **1000x** | 100.00% | 100.00% | 100.00% | 100.00% | 100.00% | 100.00% | 100.00% | 100.00% |

| **Supplemental Table 4. Simulated Deletion Dataset** | | | | | | | | | | | | | | | | |
| --- | --- | --- | --- | --- | --- | --- | --- | --- | --- | --- | --- | --- | --- | --- | --- | --- |
|  |  | **Simulated Deletions** | | | | | | | | | | | | | | |
| **Coverage** | **Allele Frequency** | **1bp** | **2bp** | **3bp** | **4bp** | **5bp** | **6bp** | **7bp** | **8bp** | **9bp** | **10bp** | **11-20bp** | **21-30bp** | **31-40bp** | **41-50bp** | **>50bp** |
| **100x** | **Total** | **813** | **346** | **256** | **211** | **168** | **134** | **129** | **107** | **117** | **93** | **604** | **321** | **155** | **82** | **59** |
|  | [0,0.03] | 168 | 51 | 53 | 38 | 36 | 27 | 25 | 23 | 17 | 20 | 123 | 65 | 29 | 16 | 13 |
|  | (0.03,0.05] | 125 | 58 | 36 | 40 | 25 | 26 | 21 | 14 | 15 | 14 | 104 | 57 | 21 | 14 | 16 |
|  | (0.05,0.1] | 123 | 53 | 40 | 31 | 23 | 15 | 16 | 17 | 16 | 6 | 67 | 47 | 28 | 9 | 6 |
|  | (0.1,0.2] | 138 | 64 | 50 | 30 | 23 | 19 | 19 | 19 | 22 | 21 | 101 | 58 | 25 | 18 | 10 |
|  | (0.2,0.3] | 81 | 40 | 24 | 25 | 19 | 12 | 17 | 11 | 12 | 9 | 57 | 36 | 15 | 7 | 5 |
|  | (0.3,0.5] | 105 | 45 | 29 | 24 | 21 | 23 | 18 | 12 | 17 | 13 | 92 | 33 | 24 | 13 | 2 |
|  | (0.5,0.8] | 66 | 32 | 22 | 20 | 19 | 11 | 10 | 8 | 15 | 8 | 54 | 23 | 12 | 4 | 7 |
|  | (0.8,1] | 7 | 3 | 2 | 3 | 2 | 1 | 3 | 3 | 3 | 2 | 6 | 2 | 1 | 1 | 0 |
| **250x** | **Total** | **840** | **352** | **265** | **219** | **172** | **135** | **131** | **107** | **119** | **97** | **624** | **334** | **161** | **84** | **59** |
|  | [0,0.03] | 203 | 76 | 71 | 56 | 46 | 43 | 36 | 26 | 25 | 24 | 167 | 92 | 36 | 24 | 23 |
|  | (0.03,0.05] | 93 | 29 | 23 | 24 | 15 | 12 | 10 | 9 | 10 | 10 | 59 | 33 | 13 | 6 | 6 |
|  | (0.05,0.1] | 133 | 56 | 41 | 31 | 23 | 13 | 17 | 19 | 16 | 10 | 72 | 50 | 32 | 10 | 6 |
|  | (0.1,0.2] | 141 | 65 | 50 | 33 | 25 | 20 | 19 | 18 | 20 | 20 | 113 | 61 | 28 | 17 | 11 |
|  | (0.2,0.3] | 86 | 42 | 25 | 25 | 18 | 12 | 18 | 10 | 12 | 10 | 58 | 40 | 14 | 8 | 4 |
|  | (0.3,0.5] | 105 | 47 | 31 | 26 | 24 | 23 | 18 | 14 | 18 | 13 | 91 | 33 | 25 | 14 | 2 |
|  | (0.5,0.8] | 72 | 34 | 21 | 21 | 19 | 11 | 10 | 8 | 15 | 8 | 56 | 23 | 11 | 4 | 7 |
|  | (0.8,1] | 7 | 3 | 3 | 3 | 2 | 1 | 3 | 3 | 3 | 2 | 8 | 2 | 2 | 1 | 0 |
| **500x** | **Total** | **842** | **352** | **267** | **220** | **173** | **136** | **130** | **108** | **119** | **98** | **630** | **336** | **161** | **84** | **59** |
|  | [0,0.03] | 204 | 76 | 73 | 55 | 47 | 43 | 35 | 26 | 24 | 24 | 167 | 86 | 36 | 24 | 23 |
|  | (0.03,0.05] | 92 | 29 | 23 | 24 | 14 | 13 | 11 | 9 | 10 | 10 | 59 | 38 | 13 | 6 | 6 |
|  | (0.05,0.1] | 133 | 55 | 40 | 33 | 24 | 13 | 17 | 18 | 18 | 10 | 75 | 52 | 32 | 10 | 6 |
|  | (0.1,0.2] | 143 | 62 | 49 | 33 | 25 | 20 | 19 | 20 | 20 | 20 | 117 | 62 | 28 | 17 | 10 |
|  | (0.2,0.3] | 86 | 46 | 27 | 25 | 18 | 12 | 18 | 10 | 11 | 10 | 56 | 39 | 14 | 8 | 5 |
|  | (0.3,0.5] | 107 | 47 | 30 | 26 | 24 | 23 | 18 | 13 | 18 | 13 | 91 | 34 | 25 | 14 | 2 |
|  | (0.5,0.8] | 70 | 34 | 22 | 21 | 19 | 11 | 9 | 9 | 15 | 9 | 58 | 23 | 11 | 4 | 7 |
|  | (0.8,1] | 7 | 3 | 3 | 3 | 2 | 1 | 3 | 3 | 3 | 2 | 7 | 2 | 2 | 1 | 0 |
| **1000x** | **Total** | **847** | **353** | **269** | **222** | **172** | **137** | **133** | **107** | **121** | **98** | **631** | **336** | **162** | **83** | **59** |
|  | [0,0.03] | 202 | 75 | 72 | 55 | 46 | 43 | 35 | 25 | 22 | 24 | 164 | 88 | 35 | 24 | 23 |
|  | (0.03,0.05] | 92 | 31 | 24 | 26 | 16 | 13 | 11 | 10 | 12 | 10 | 63 | 38 | 14 | 6 | 6 |
|  | (0.05,0.1] | 134 | 55 | 40 | 32 | 23 | 13 | 18 | 18 | 19 | 10 | 74 | 51 | 32 | 10 | 6 |
|  | (0.1,0.2] | 146 | 63 | 51 | 32 | 25 | 20 | 19 | 19 | 20 | 19 | 117 | 59 | 28 | 16 | 10 |
|  | (0.2,0.3] | 87 | 44 | 27 | 27 | 18 | 12 | 18 | 11 | 11 | 11 | 56 | 40 | 15 | 9 | 5 |
|  | (0.3,0.5] | 108 | 47 | 30 | 26 | 23 | 24 | 18 | 12 | 19 | 13 | 93 | 35 | 25 | 13 | 2 |
|  | (0.5,0.8] | 71 | 35 | 22 | 21 | 19 | 11 | 11 | 9 | 15 | 9 | 57 | 23 | 11 | 4 | 7 |
|  | (0.8,1] | 7 | 3 | 3 | 3 | 2 | 1 | 3 | 3 | 3 | 2 | 7 | 2 | 2 | 1 | 0 |
|  | **Grand Total** | **3342** | **1403** | **1057** | **872** | **685** | **542** | **523** | **429** | **476** | **386** | **2489** | **1327** | **639** | **333** | **236** |
| Values indicate the number of simulated random test fragments  Note: [0,0.03] = 0% to 3%, (0.03,0.05] = >3% to 5%, (0.05,0.1] = >5 to 10%, etc. | | | | | | | | | | | | | | | | |

| **Supplemental Table 5. Sensitivity for Deletion Detection** | | | | | | | | | | | | | | | | |
| --- | --- | --- | --- | --- | --- | --- | --- | --- | --- | --- | --- | --- | --- | --- | --- | --- |
| **Coverage** | **Allele Frequency** | **Simulated Deletions** | | | | | | | | | | | | | | |
|  |  | **1bp** | **2bp** | **3bp** | **4bp** | **5bp** | **6bp** | **7bp** | **8bp** | **9bp** | **10bp** | **11-20bp** | **21-30bp** | **31-40bp** | **41-50bp** | **>50bp** |
| 100x | [0,0.03] | 79.76% | 76.47% | 67.92% | 78.95% | 91.67% | 74.07% | 84.00% | 65.22% | 70.59% | 85.00% | 65.04% | 56.92% | 51.72% | 50.00% | 33.33% |
|  | (0.03,0.05] | 90.40% | 84.48% | 86.11% | 75.00% | 80.00% | 69.23% | 61.90% | 92.86% | 93.33% | 71.43% | 70.19% | 47.37% | 42.86% | 57.14% | 60.00% |
|  | (0.05,0.1] | 94.31% | 96.23% | 82.50% | 90.32% | 95.65% | 93.33% | 93.75% | 88.24% | 93.75% | 100% | 92.54% | 91.49% | 96.43% | 88.89% | 50.00% |
|  | (0.1,0.2] | 100% | 100% | 100% | 100% | 100% | 94.74% | 100% | 100% | 95.45% | 100% | 100% | 98.28% | 96.00% | 100% | 100% |
|  | (0.2,0.3] | 100% | 100% | 100% | 100% | 100% | 100% | 100% | 100% | 100% | 100% | 100% | 100% | 100% | 100% | 100% |
|  | (0.3,0.5] | 100% | 100% | 100% | 100% | 100% | 100% | 100% | 100% | 100% | 100% | 100% | 100% | 100% | 100% | 100% |
|  | (0.5,0.8] | 100% | 100% | 100% | 100% | 100% | 100% | 100% | 100% | 100% | 100% | 100% | 100% | 100% | 100% | 100% |
|  | (0.8,1] | 100% | 100% | 100% | 100% | 100% | 100% | 100% | 100% | 100% | 100% | 100% | 100% | 100% | 100% | NA |
| 250x | [0,0.03] | 63.05% | 59.21% | 63.38% | 55.36% | 65.22% | 58.14% | 58.33% | 34.62% | 68.00% | 66.67% | 56.29% | 54.35% | 50.00% | 33.33% | 53.85% |
|  | (0.03,0.05] | 98.92% | 89.66% | 95.65% | 95.83% | 100% | 100% | 80.00% | 88.89% | 90.00% | 100% | 89.83% | 93.94% | 84.62% | 100% | 100% |
|  | (0.05,0.1] | 100% | 100% | 100% | 100% | 100% | 100% | 100% | 100% | 100% | 100% | 100% | 100% | 100% | 100% | 100% |
|  | (0.1,0.2] | 100% | 100% | 100% | 100% | 100% | 100% | 100% | 100% | 100% | 100% | 100% | 100% | 100% | 100% | 100% |
|  | (0.2,0.3] | 100% | 100% | 100% | 100% | 100% | 100% | 100% | 100% | 100% | 100% | 100% | 100% | 100% | 100% | 100% |
|  | (0.3,0.5] | 100% | 100% | 100% | 100% | 100% | 100% | 100% | 100% | 100% | 100% | 100% | 100% | 100% | 100% | 100% |
|  | (0.5,0.8] | 100% | 100% | 100% | 100% | 100% | 100% | 100% | 100% | 100% | 100% | 100% | 100% | 100% | 100% | 100% |
|  | (0.8,1] | 100% | 100% | 100% | 100% | 100% | 100% | 100% | 100% | 100% | 100% | 100% | 100% | 100% | 100% | NA |
| 500x | [0,0.03] | 62.25% | 56.58% | 60.27% | 56.36% | 57.45% | 46.51% | 48.57% | 61.54% | 70.83% | 50.00% | 55.09% | 48.84% | 66.67% | 54.17% | 53.85% |
|  | (0.03,0.05] | 100% | 100% | 100% | 100% | 100% | 100% | 100% | 100% | 100% | 100% | 100% | 97.37% | 100% | 100% | 100% |
|  | (0.05,0.1] | 100% | 100% | 100% | 100% | 100% | 100% | 100% | 100% | 100% | 100% | 100% | 100% | 100% | 100% | 100% |
|  | (0.1,0.2] | 100% | 100% | 100% | 100% | 100% | 100% | 100% | 100% | 100% | 100% | 100% | 100% | 100% | 100% | 100% |
|  | (0.2,0.3] | 100% | 100% | 100% | 100% | 100% | 100% | 100% | 100% | 100% | 100% | 100% | 100% | 100% | 100% | 100% |
|  | (0.3,0.5] | 100% | 100% | 100% | 100% | 100% | 100% | 100% | 100% | 100% | 100% | 100% | 100% | 100% | 100% | 100% |
|  | (0.5,0.8] | 100% | 100% | 100% | 100% | 100% | 100% | 100% | 100% | 100% | 100% | 100% | 100% | 100% | 100% | 100% |
|  | (0.8,1] | 100% | 100% | 100% | 100% | 100% | 100% | 100% | 100% | 100% | 100% | 100% | 100% | 100% | 100% | NA |
| 1000x | [0,0.03] | 70.79% | 65.33% | 73.61% | 65.45% | 65.22% | 62.79% | 60.00% | 72.00% | 72.73% | 66.67% | 62.20% | 55.68% | 60.00% | 54.17% | 69.23% |
|  | (0.03,0.05] | 100% | 100% | 100% | 100% | 100% | 100% | 100% | 100% | 100% | 100% | 100% | 100% | 100% | 100% | 100% |
|  | (0.05,0.1] | 100% | 100% | 100% | 100% | 100% | 100% | 100% | 100% | 100% | 100% | 100% | 100% | 100% | 100% | 100% |
|  | (0.1,0.2] | 100% | 100% | 100% | 100% | 100% | 100% | 100% | 100% | 100% | 100% | 100% | 100% | 100% | 100% | 100% |
|  | (0.2,0.3] | 100% | 100% | 100% | 100% | 100% | 100% | 100% | 100% | 100% | 100% | 100% | 100% | 100% | 100% | 100% |
|  | (0.3,0.5] | 100% | 100% | 100% | 100% | 100% | 100% | 100% | 100% | 100% | 100% | 100% | 100% | 100% | 100% | 100% |
|  | (0.5,0.8] | 100% | 100% | 100% | 100% | 100% | 100% | 100% | 100% | 100% | 100% | 100% | 100% | 100% | 100% | 100% |
|  | (0.8,1] | 100% | 100% | 100% | 100% | 100% | 100% | 100% | 100% | 100% | 100% | 100% | 100% | 100% | 100% | NA |
| Note: [0,0.03] = 0% to 3%, (0.03,0.05] = >3% to 5%, (0.05,0.1] = >5 to 10%, etc. | | | | | | | | | | | | | | | | |

| **Supplemental Table 6. Specificity for Deletion Detection** | | | | | | | | | | | | | | | | |
| --- | --- | --- | --- | --- | --- | --- | --- | --- | --- | --- | --- | --- | --- | --- | --- | --- |
| Coverage | Allele Frequency | **Simulated Deletions** | | | | | | | | | | | | | | |
|  |  | 1bp | 2bp | 3bp | 4bp | 5bp | 6bp | 7bp | 8bp | 9bp | 10bp | 11-20bp | 21-30bp | 31-40bp | 41-50bp | >50bp |
| 100x | [0,0.03] | 100% | 100% | 100% | 100% | 100% | 100% | 100% | 100% | 100% | 100% | 100% | 100% | 100% | 100% | 100% |
|  | (0.03,0.05] | 100% | 100% | 100% | 100% | 100% | 100% | 100% | 100% | 100% | 100% | 100% | 100% | 100% | 100% | 100% |
|  | (0.05,0.1] | 100% | 100% | 100% | 100% | 100% | 100% | 100% | 100% | 100% | 100% | 100% | 100% | 100% | 100% | 100% |
|  | (0.1,0.2] | 100% | 100% | 100% | 100% | 100% | 100% | 100% | 100% | 100% | 100% | 100% | 100% | 100% | 100% | 100% |
|  | (0.2,0.3] | 100% | 100% | 100% | 100% | 100% | 100% | 100% | 100% | 100% | 100% | 100% | 100% | 100% | 100% | 100% |
|  | (0.3,0.5] | 100% | 100% | 100% | 100% | 100% | 100% | 100% | 100% | 100% | 100% | 100% | 100% | 100% | 100% | 100% |
|  | (0.5,0.8] | 100% | 100% | 100% | 100% | 100% | 100% | 100% | 100% | 100% | 100% | 100% | 100% | 100% | 100% | 100% |
|  | (0.8,1] | 100% | 100% | 100% | 100% | 100% | 100% | 100% | 100% | 100% | 100% | 100% | 100% | 100% | 100% | NA |
| 250x | [0,0.03] | 100% | 100% | 100% | 100% | 100% | 100% | 100% | 100% | 100% | 100% | 100% | 100% | 100% | 100% | 100% |
|  | (0.03,0.05] | 100% | 100% | 100% | 100% | 100% | 100% | 100% | 100% | 100% | 100% | 100% | 100% | 100% | 100% | 100% |
|  | (0.05,0.1] | 100% | 100% | 100% | 100% | 100% | 100% | 100% | 100% | 100% | 100% | 100% | 100% | 100% | 100% | 100% |
|  | (0.1,0.2] | 100% | 100% | 100% | 100% | 100% | 100% | 100% | 100% | 100% | 100% | 100% | 100% | 100% | 100% | 100% |
|  | (0.2,0.3] | 100% | 100% | 100% | 100% | 100% | 100% | 100% | 100% | 100% | 100% | 100% | 100% | 100% | 100% | 100% |
|  | (0.3,0.5] | 100% | 100% | 100% | 100% | 100% | 100% | 100% | 100% | 100% | 100% | 100% | 100% | 100% | 100% | 100% |
|  | (0.5,0.8] | 100% | 100% | 100% | 100% | 100% | 100% | 100% | 100% | 100% | 100% | 100% | 100% | 100% | 100% | 100% |
|  | (0.8,1] | 100% | 100% | 100% | 100% | 100% | 100% | 100% | 100% | 100% | 100% | 100% | 100% | 100% | 100% | NA |
| 500x | [0,0.03] | 100% | 100% | 100% | 100% | 100% | 100% | 100% | 100% | 100% | 100% | 100% | 100% | 100% | 100% | 100% |
|  | (0.03,0.05] | 100% | 100% | 100% | 100% | 100% | 100% | 100% | 100% | 100% | 100% | 100% | 100% | 100% | 100% | 100% |
|  | (0.05,0.1] | 100% | 100% | 100% | 100% | 100% | 100% | 100% | 100% | 100% | 100% | 100% | 100% | 100% | 100% | 100% |
|  | (0.1,0.2] | 100% | 100% | 100% | 100% | 100% | 100% | 100% | 100% | 100% | 100% | 100% | 100% | 100% | 100% | 100% |
|  | (0.2,0.3] | 100% | 100% | 100% | 100% | 100% | 100% | 100% | 100% | 100% | 100% | 100% | 100% | 100% | 100% | 100% |
|  | (0.3,0.5] | 100% | 100% | 100% | 100% | 100% | 100% | 100% | 100% | 100% | 100% | 100% | 100% | 100% | 100% | 100% |
|  | (0.5,0.8] | 100% | 100% | 100% | 100% | 100% | 100% | 100% | 100% | 100% | 100% | 100% | 100% | 100% | 100% | 100% |
|  | (0.8,1] | 100% | 100% | 100% | 100% | 100% | 100% | 100% | 100% | 100% | 100% | 100% | 100% | 100% | 100% | NA |
| 1000x | [0,0.03] | 100% | 100% | 100% | 100% | 100% | 100% | 100% | 100% | 100% | 100% | 100% | 100% | 100% | 100% | 100% |
|  | (0.03,0.05] | 100% | 100% | 100% | 100% | 100% | 100% | 100% | 100% | 100% | 100% | 100% | 100% | 100% | 100% | 100% |
|  | (0.05,0.1] | 100% | 100% | 100% | 100% | 100% | 100% | 100% | 100% | 100% | 100% | 100% | 100% | 100% | 100% | 100% |
|  | (0.1,0.2] | 100% | 100% | 100% | 100% | 100% | 100% | 100% | 100% | 100% | 100% | 100% | 100% | 100% | 100% | 100% |
|  | (0.2,0.3] | 100% | 100% | 100% | 100% | 100% | 100% | 100% | 100% | 100% | 100% | 100% | 100% | 100% | 100% | 100% |
|  | (0.3,0.5] | 100% | 100% | 100% | 100% | 100% | 100% | 100% | 100% | 100% | 100% | 100% | 100% | 100% | 100% | 100% |
|  | (0.5,0.8] | 100% | 100% | 100% | 100% | 100% | 100% | 100% | 100% | 100% | 100% | 100% | 100% | 100% | 100% | 100% |
|  | (0.8,1] | 100% | 100% | 100% | 100% | 100% | 100% | 100% | 100% | 100% | 100% | 100% | 100% | 100% | 100% | NA |
| Note: [0,0.03] = 0% to 3%, (0.03,0.05] = >3% to 5%, (0.05,0.1] = >5 to 10%, etc. | | | | | | | | | | | | | | | | |

| **Supplemental Table 7. Simulated Insertion Dataset** | | | | | | | | | | | | | | | | |
| --- | --- | --- | --- | --- | --- | --- | --- | --- | --- | --- | --- | --- | --- | --- | --- | --- |
| **Coverage** | **Allele Frequency** | **Simulated Deletions** | | | | | | | | | | | | | | |
|  |  | **1bp** | **2bp** | **3bp** | **4bp** | **5bp** | **6bp** | **7bp** | **8bp** | **9bp** | **10bp** | **11-20bp** | **21-30bp** | **31-40bp** | **41-50bp** | **>50bp** |
| **100X** | **Total** | **809** | **305** | **239** | **205** | **177** | **158** | **150** | **113** | **105** | **92** | **683** | **330** | **171** | **78** | **54** |
|  | [0,0.03] | 144 | 56 | 39 | 36 | 33 | 37 | 23 | 18 | 26 | 19 | 128 | 71 | 34 | 16 | 12 |
|  | (0.03,0.05] | 129 | 56 | 40 | 28 | 30 | 21 | 25 | 17 | 15 | 19 | 122 | 56 | 25 | 13 | 7 |
|  | (0.05,0.1] | 134 | 38 | 35 | 28 | 27 | 17 | 21 | 12 | 12 | 13 | 98 | 46 | 23 | 10 | 8 |
|  | (0.1,0.2] | 161 | 60 | 41 | 38 | 25 | 31 | 29 | 20 | 15 | 15 | 125 | 53 | 38 | 15 | 13 |
|  | (0.2,0.3] | 86 | 26 | 28 | 24 | 29 | 17 | 11 | 20 | 11 | 11 | 68 | 34 | 17 | 7 | 4 |
|  | (0.3,0.5] | 87 | 32 | 31 | 28 | 19 | 20 | 23 | 18 | 11 | 11 | 71 | 41 | 19 | 11 | 5 |
|  | (0.5,0.8] | 59 | 31 | 21 | 20 | 12 | 14 | 15 | 8 | 12 | 4 | 58 | 24 | 9 | 5 | 5 |
|  | (0.8,1] | 9 | 6 | 4 | 3 | 2 | 1 | 3 | 0 | 3 | 0 | 13 | 5 | 6 | 1 | 0 |
| **250X** | **Total** | **836** | **310** | **240** | **211** | **180** | **161** | **152** | **119** | **110** | **97** | **696** | **332** | **176** | **79** | **55** |
|  | [0,0.03] | 202 | 83 | 56 | 48 | 48 | 44 | 27 | 30 | 34 | 31 | 179 | 103 | 42 | 21 | 13 |
|  | (0.03,0.05] | 71 | 28 | 23 | 19 | 17 | 17 | 19 | 6 | 10 | 9 | 75 | 24 | 18 | 7 | 7 |
|  | (0.05,0.1] | 138 | 40 | 33 | 31 | 27 | 17 | 25 | 15 | 13 | 14 | 93 | 44 | 26 | 10 | 9 |
|  | (0.1,0.2] | 178 | 62 | 46 | 38 | 26 | 31 | 29 | 21 | 15 | 18 | 132 | 54 | 39 | 17 | 12 |
|  | (0.2,0.3] | 83 | 26 | 26 | 23 | 28 | 17 | 12 | 19 | 12 | 9 | 70 | 36 | 19 | 6 | 4 |
|  | (0.3,0.5] | 94 | 33 | 32 | 27 | 20 | 20 | 23 | 19 | 10 | 12 | 76 | 43 | 17 | 12 | 5 |
|  | (0.5,0.8] | 60 | 32 | 21 | 22 | 12 | 14 | 13 | 9 | 13 | 4 | 58 | 21 | 9 | 5 | 5 |
|  | (0.8,1] | 10 | 6 | 3 | 3 | 2 | 1 | 4 | 0 | 3 | 0 | 13 | 7 | 6 | 1 | 0 |
| **500X** | **Total** | **834** | **313** | **244** | **211** | **180** | **163** | **152** | **120** | **111** | **97** | **699** | **333** | **176** | **79** | **55** |
|  | [0,0.03] | 201 | 85 | 54 | 46 | 47 | 44 | 27 | 31 | 30 | 29 | 175 | 101 | 41 | 21 | 12 |
|  | (0.03,0.05] | 72 | 28 | 24 | 22 | 16 | 16 | 14 | 6 | 14 | 9 | 81 | 26 | 19 | 7 | 8 |
|  | (0.05,0.1] | 138 | 41 | 35 | 30 | 29 | 18 | 30 | 15 | 14 | 16 | 95 | 44 | 25 | 10 | 8 |
|  | (0.1,0.2] | 177 | 62 | 48 | 38 | 26 | 33 | 29 | 21 | 15 | 18 | 132 | 54 | 39 | 17 | 13 |
|  | (0.2,0.3] | 83 | 26 | 27 | 22 | 29 | 17 | 12 | 19 | 12 | 9 | 67 | 36 | 19 | 6 | 4 |
|  | (0.3,0.5] | 94 | 33 | 31 | 28 | 19 | 20 | 23 | 19 | 10 | 12 | 77 | 44 | 18 | 12 | 5 |
|  | (0.5,0.8] | 60 | 32 | 21 | 22 | 12 | 14 | 13 | 9 | 13 | 4 | 59 | 21 | 9 | 5 | 5 |
|  | (0.8,1] | 9 | 6 | 4 | 3 | 2 | 1 | 4 | 0 | 3 | 0 | 13 | 7 | 6 | 1 | 0 |
| **1000X** | **Total** | **838** | **312** | **242** | **210** | **180** | **161** | **153** | **121** | **111** | **99** | **708** | **333** | **178** | **79** | **55** |
|  | [0,0.03] | 200 | 84 | 54 | 46 | 46 | 43 | 26 | 30 | 30 | 31 | 175 | 99 | 44 | 21 | 11 |
|  | (0.03,0.05] | 72 | 29 | 24 | 21 | 17 | 17 | 16 | 6 | 14 | 9 | 79 | 28 | 19 | 6 | 9 |
|  | (0.05,0.1] | 141 | 40 | 34 | 30 | 29 | 16 | 30 | 17 | 14 | 16 | 99 | 42 | 23 | 11 | 8 |
|  | (0.1,0.2] | 177 | 62 | 47 | 38 | 26 | 33 | 29 | 21 | 15 | 17 | 130 | 56 | 40 | 17 | 13 |
|  | (0.2,0.3] | 83 | 26 | 26 | 22 | 29 | 17 | 12 | 19 | 12 | 10 | 73 | 36 | 18 | 6 | 4 |
|  | (0.3,0.5] | 94 | 33 | 32 | 28 | 19 | 20 | 22 | 19 | 10 | 12 | 78 | 43 | 19 | 12 | 5 |
|  | (0.5,0.8] | 62 | 32 | 22 | 22 | 12 | 14 | 14 | 9 | 13 | 4 | 60 | 23 | 9 | 5 | 5 |
|  | (0.8,1] | 9 | 6 | 3 | 3 | 2 | 1 | 4 | 0 | 3 | 0 | 14 | 6 | 6 | 1 | 0 |
|  | **Grand Total** | **3317** | **1240** | **965** | **837** | **717** | **643** | **607** | **473** | **437** | **385** | **2786** | **1328** | **701** | **315** | **219** |
| Values indicate the number of simulated random test fragments  Note: [0,0.03] = 0% to 3%, (0.03,0.05] = >3% to 5%, (0.05,0.1] = >5 to 10%, etc. | | | | | | | | | | | | | | | | |

| **Supplemental Table 8. Sensitivity for Insertion Detection** | | | | | | | | | | | | | | | | |
| --- | --- | --- | --- | --- | --- | --- | --- | --- | --- | --- | --- | --- | --- | --- | --- | --- |
| **Coverage** | **Allele Frequency** | **Simulated Deletions** | | | | | | | | | | | | | | |
|  |  | **1bp** | **2bp** | **3bp** | **4bp** | **5bp** | **6bp** | **7bp** | **8bp** | **9bp** | **10bp** | **11-20bp** | **21-30bp** | **31-40bp** | **41-50bp** | **>50bp** |
| 100x | [0,0.03] | 76.39% | 73.21% | 74.36% | 72.22% | 72.73% | 59.46% | 73.91% | 61.11% | 69.23% | 68.42% | 64.06% | 40.85% | 17.65% | 25.00% | 20.00% |
|  | (0.03,0.05] | 85.27% | 80.36% | 90.00% | 82.14% | 70.00% | 71.43% | 88.00% | 82.35% | 66.67% | 47.37% | 63.11% | 51.79% | 44.00% | 7.69% | 0.00% |
|  | (0.05,0.1] | 92.54% | 100% | 88.57% | 85.71% | 96.30% | 100% | 85.71% | 100% | 83.33% | 92.31% | 94.90% | 84.78% | 78.26% | 90.00% | 33.33% |
|  | (0.1,0.2] | 99.38% | 100% | 100% | 100% | 100% | 100% | 100% | 100% | 100% | 100% | 97.60% | 100% | 89.47% | 93.33% | 100% |
|  | (0.2,0.3] | 100% | 100% | 100% | 100% | 100% | 100% | 100% | 100% | 100% | 100% | 100% | 100% | 100% | 100% | 100% |
|  | (0.3,0.5] | 100% | 100% | 100% | 100% | 100% | 100% | 100% | 100% | 100% | 100% | 100% | 100% | 100% | 100% | 100% |
|  | (0.5,0.8] | 100% | 100% | 100% | 100% | 100% | 100% | 100% | 100% | 100% | 100% | 100% | 100% | 100% | 100% | 100% |
|  | (0.8,1] | 100% | 100% | 100% | 100% | 100% | 100% | 100% | NA | 100% | NA | 100% | 100% | 100% | 100% | NA |
| 250x | [0,0.03] | 58.42% | 61.45% | 53.57% | 64.58% | 52.08% | 68.18% | 48.15% | 66.67% | 52.94% | 58.06% | 44.69% | 24.27% | 23.81% | 23.81% | 7.69% |
|  | (0.03,0.05] | 95.77% | 96.43% | 95.65% | 94.74% | 100% | 94.12% | 94.74% | 100% | 80.00% | 77.78% | 89.33% | 83.33% | 83.33% | 71.43% | 14.29% |
|  | (0.05,0.1] | 100% | 100% | 96.97% | 100% | 100% | 100% | 100% | 100% | 100% | 100% | 100% | 93.18% | 96.15% | 60.00% | 100% |
|  | (0.1,0.2] | 100% | 100% | 100% | 100% | 100% | 100% | 100% | 100% | 100% | 100% | 100% | 100% | 100% | 100% | 100% |
|  | (0.2,0.3] | 100% | 100% | 100% | 100% | 100% | 100% | 100% | 100% | 100% | 100% | 100% | 100% | 100% | 100% | 100% |
|  | (0.3,0.5] | 100% | 100% | 100% | 100% | 100% | 100% | 100% | 100% | 100% | 100% | 100% | 100% | 100% | 100% | 100% |
|  | (0.5,0.8] | 100% | 100% | 100% | 100% | 100% | 100% | 100% | 100% | 100% | 100% | 100% | 100% | 100% | 100% | 100% |
|  | (0.8,1] | 100% | 100% | 100% | 100% | 100% | 100% | 100% | NA | 100% | NA | 100% | 100% | 100% | 100% | NA |
| 500x | [0,0.03] | 57.21% | 58.82% | 72.22% | 60.87% | 48.94% | 61.36% | 40.74% | 70.97% | 60.00% | 62.07% | 44.00% | 29.70% | 34.15% | 19.05% | 0.00% |
|  | (0.03,0.05] | 100% | 100% | 100% | 100% | 100% | 100% | 100% | 83.33% | 100% | 100% | 97.53% | 100% | 78.95% | 57.14% | 66.67% |
|  | (0.05,0.1] | 100% | 100% | 100% | 100% | 100% | 100% | 100% | 100% | 100% | 100% | 100% | 100% | 100% | 100% | 100% |
|  | (0.1,0.2] | 100% | 100% | 100% | 100% | 100% | 100% | 100% | 100% | 100% | 100% | 100% | 100% | 100% | 100% | 100% |
|  | (0.2,0.3] | 100% | 100% | 100% | 100% | 100% | 100% | 100% | 100% | 100% | 100% | 100% | 100% | 100% | 100% | 100% |
|  | (0.3,0.5] | 100% | 100% | 100% | 100% | 100% | 100% | 100% | 100% | 100% | 100% | 100% | 100% | 100% | 100% | 100% |
|  | (0.5,0.8] | 100% | 100% | 100% | 100% | 100% | 100% | 100% | 100% | 100% | 100% | 100% | 100% | 100% | 100% | 100% |
|  | (0.8,1] | 100% | 100% | 100% | 100% | 100% | 100% | 100% | NA | 100% | NA | 100% | 100% | 100% | 100% | NA |
| 1000x | [0,0.03] | 62.00% | 61.90% | 75.93% | 69.57% | 56.52% | 74.42% | 46.15% | 73.33% | 70.00% | 67.74% | 53.71% | 41.41% | 22.73% | 19.05% | 0.00% |
|  | (0.03,0.05] | 100% | 100% | 100% | 100% | 100% | 100% | 100% | 100% | 100% | 100% | 100% | 100% | 89.47% | 66.67% | 100% |
|  | (0.05,0.1] | 100% | 100% | 100% | 100% | 100% | 100% | 100% | 100% | 100% | 100% | 100% | 100% | 100% | 100% | 100% |
|  | (0.1,0.2] | 100% | 100% | 100% | 100% | 100% | 100% | 100% | 100% | 100% | 100% | 100% | 100% | 100% | 100% | 100% |
|  | (0.2,0.3] | 100% | 100% | 100% | 100% | 100% | 100% | 100% | 100% | 100% | 100% | 100% | 100% | 100% | 100% | 100% |
|  | (0.3,0.5] | 100% | 100% | 100% | 100% | 100% | 100% | 100% | 100% | 100% | 100% | 100% | 100% | 100% | 100% | 100% |
|  | (0.5,0.8] | 100% | 100% | 100% | 100% | 100% | 100% | 100% | 100% | 100% | 100% | 100% | 100% | 100% | 100% | 100% |
|  | (0.8,1] | 100% | 100% | 100% | 100% | 100% | 100% | 100% | NA | 100% | NA | 100% | 100% | 100% | 100% | NA |
| Note: [0,0.03] = 0% to 3%, (0.03,0.05] = >3% to 5%, (0.05,0.1] = >5 to 10%, etc. | | | | | | | | | | | | | | | | |

| **Supplemental Table 9. Specificity for Insertion Detection** | | | | | | | | | | | | | | | | |
| --- | --- | --- | --- | --- | --- | --- | --- | --- | --- | --- | --- | --- | --- | --- | --- | --- |
| Coverage | Allele Frequency | **Simulated Deletions** | | | | | | | | | | | | | | |
|  |  | 1bp | 2bp | 3bp | 4bp | 5bp | 6bp | 7bp | 8bp | 9bp | 10bp | 11-20bp | 21-30bp | 31-40bp | 41-50bp | >50bp |
| 100x | [0,0.03] | 100% | 100% | 100% | 100% | 100% | 100% | 100% | 100% | 100% | 100% | 100% | 100% | 100% | 100% | 100% |
|  | (0.03,0.05] | 100% | 100% | 100% | 100% | 100% | 100% | 100% | 100% | 100% | 100% | 100% | 100% | 100% | 100% | 100% |
|  | (0.05,0.1] | 100% | 100% | 100% | 100% | 100% | 100% | 100% | 100% | 100% | 100% | 100% | 100% | 100% | 100% | 100% |
|  | (0.1,0.2] | 100% | 100% | 100% | 100% | 100% | 100% | 100% | 100% | 100% | 100% | 100% | 100% | 100% | 100% | 100% |
|  | (0.2,0.3] | 100% | 100% | 100% | 100% | 100% | 100% | 100% | 100% | 100% | 100% | 100% | 100% | 100% | 100% | 100% |
|  | (0.3,0.5] | 100% | 100% | 100% | 100% | 100% | 100% | 100% | 100% | 100% | 100% | 100% | 100% | 100% | 100% | 100% |
|  | (0.5,0.8] | 100% | 100% | 100% | 100% | 100% | 100% | 100% | 100% | 100% | 100% | 100% | 100% | 100% | 100% | 100% |
|  | (0.8,1] | 100% | 100% | 100% | 100% | 100% | 100% | 100% | 100% | 100% | 100% | 100% | 100% | 100% | 100% | NA |
| 250x | [0,0.03] | 100% | 100% | 100% | 100% | 100% | 100% | 100% | 100% | 100% | 100% | 100% | 100% | 100% | 100% | 100% |
|  | (0.03,0.05] | 100% | 100% | 100% | 100% | 100% | 100% | 100% | 100% | 100% | 100% | 100% | 100% | 100% | 100% | 100% |
|  | (0.05,0.1] | 100% | 100% | 100% | 100% | 100% | 100% | 100% | 100% | 100% | 100% | 100% | 100% | 100% | 100% | 100% |
|  | (0.1,0.2] | 100% | 100% | 100% | 100% | 100% | 100% | 100% | 100% | 100% | 100% | 100% | 100% | 100% | 100% | 100% |
|  | (0.2,0.3] | 100% | 100% | 100% | 100% | 100% | 100% | 100% | 100% | 100% | 100% | 100% | 100% | 100% | 100% | 100% |
|  | (0.3,0.5] | 100% | 100% | 100% | 100% | 100% | 100% | 100% | 100% | 100% | 100% | 100% | 100% | 100% | 100% | 100% |
|  | (0.5,0.8] | 100% | 100% | 100% | 100% | 100% | 100% | 100% | 100% | 100% | 100% | 100% | 100% | 100% | 100% | 100% |
|  | (0.8,1] | 100% | 100% | 100% | 100% | 100% | 100% | 100% | 100% | 100% | 100% | 100% | 100% | 100% | 100% | NA |
| 500x | [0,0.03] | 100% | 100% | 100% | 100% | 100% | 100% | 100% | 100% | 100% | 100% | 100% | 100% | 100% | 100% | 100% |
|  | (0.03,0.05] | 100% | 100% | 100% | 100% | 100% | 100% | 100% | 100% | 100% | 100% | 100% | 100% | 100% | 100% | 100% |
|  | (0.05,0.1] | 100% | 100% | 100% | 100% | 100% | 100% | 100% | 100% | 100% | 100% | 100% | 100% | 100% | 100% | 100% |
|  | (0.1,0.2] | 100% | 100% | 100% | 100% | 100% | 100% | 100% | 100% | 100% | 100% | 100% | 100% | 100% | 100% | 100% |
|  | (0.2,0.3] | 100% | 100% | 100% | 100% | 100% | 100% | 100% | 100% | 100% | 100% | 100% | 100% | 100% | 100% | 100% |
|  | (0.3,0.5] | 100% | 100% | 100% | 100% | 100% | 100% | 100% | 100% | 100% | 100% | 100% | 100% | 100% | 100% | 100% |
|  | (0.5,0.8] | 100% | 100% | 100% | 100% | 100% | 100% | 100% | 100% | 100% | 100% | 100% | 100% | 100% | 100% | 100% |
|  | (0.8,1] | 100% | 100% | 100% | 100% | 100% | 100% | 100% | 100% | 100% | 100% | 100% | 100% | 100% | 100% | NA |
| 1000x | [0,0.03] | 100% | 100% | 100% | 100% | 100% | 100% | 100% | 100% | 100% | 100% | 100% | 100% | 100% | 100% | 100% |
|  | (0.03,0.05] | 100% | 100% | 100% | 100% | 100% | 100% | 100% | 100% | 100% | 100% | 100% | 100% | 100% | 100% | 100% |
|  | (0.05,0.1] | 100% | 100% | 100% | 100% | 100% | 100% | 100% | 100% | 100% | 100% | 100% | 100% | 100% | 100% | 100% |
|  | (0.1,0.2] | 100% | 100% | 100% | 100% | 100% | 100% | 100% | 100% | 100% | 100% | 100% | 100% | 100% | 100% | 100% |
|  | (0.2,0.3] | 100% | 100% | 100% | 100% | 100% | 100% | 100% | 100% | 100% | 100% | 100% | 100% | 100% | 100% | 100% |
|  | (0.3,0.5] | 100% | 100% | 100% | 100% | 100% | 100% | 100% | 100% | 100% | 100% | 100% | 100% | 100% | 100% | 100% |
|  | (0.5,0.8] | 100% | 100% | 100% | 100% | 100% | 100% | 100% | 100% | 100% | 100% | 100% | 100% | 100% | 100% | 100% |
|  | (0.8,1] | 100% | 100% | 100% | 100% | 100% | 100% | 100% | 100% | 100% | 100% | 100% | 100% | 100% | 100% | NA |

| **Supplemental Table 10. TumorNext Solid Tumor Panel** | | | | | | | | | |
| --- | --- | --- | --- | --- | --- | --- | --- | --- | --- |
| Genes with full exon coverage | | | | | | | | | |
| ABL1 | BCL2L1 | CSF1R | FANCA | GNAQ | MAP2K2 | MYD88 | PDGFRB | RB1 | TET2 |
| ABL2* | BCL2L2 | CTNNB1 | FANCC | GNAS | MAP2K4 | NBN | PIK3CA | RET | TMEM127 |
| AKT1 | BMPR1A | DDR2 | FANCD2 | HRAS | MAX | NF1 | PIK3CB | ROS1 | TOP1 |
| AKT2 | BRAF | DNMT3A | FBXW7 | HSP90AA1 | MDM2 | NF2 | PIK3CG | RUNX1 | TP53 |
| AKT3 | BRCA1 | EGFR | FGFR1 | IDH1 | MET | NOTCH1 | PIK3R1 | SDHA | TSC1 |
| ALK | BRCA2 | EPHA2 | FGFR2 | IDH2 | MITF | NOTCH2 | PIK3R2** | SDHAF2 | TSC2 |
| APC | BRIP1 | EPHA3 | FGFR3 | IGF1R | MLH1 | NOTCH3 | PMS2*** | SDHB | VHL |
| AR | BTK | EPHA5 | FGFR4 | IL7R | MLL | NOTCH4 | PTCH1 | SDHC |  |
| ARAF | CBL | EPHA7 | FH | JAK1 | MPL | NRAS | PTEN | SDHD |  |
| ATM | CCND1 | EPHB1 | FLCN | JAK2 | MRE11A | NTRK1 | PTPN11 | SMAD4 |  |
| AURKA | CCND3 | ERBB2 | FLT1 | JAK3 | MSH2 | PAK7 | RAD50 | SMARCB1 |  |
| AURKB | CDH1 | ERBB3 | FLT3 | KDR | MSH6 | PALB2 | RAD51C | SMO |  |
| AXL | CDK4 | ERBB4 | FLT4 | KIT | MTOR | PARP1 | RAD51D | SRC |  |
| BARD1 | CDKN2A | ESR1 | FOXL2 | KRAS | MUTYH | PARP2 | RAF1 | STAT3 |  |
| BCL2 | CHEK2 | EZH2 | GNA11 | MAP2K1 | MYCN | PDGFRA | RARA | STK11 |  |
| Genes analyzed for stuctural variants | | | | | | | | | |
| ALK | BRAF | BRCA2 | FGFR2 | KIT | NTRK1 | RAF1 | ROS1 |  |  |
| BCL2 | BRCA1 | FGFR1 | FGFR3 | NOTCH2 | PDGFRA | RET |  |  |  |
| *ABL2 exon 2 is not included in the assay  **PIK3R2 exon 13 is not included in the assay  ***Only *PMS2* exons 1-10 are analyzed | | | | | | | | | |

| **Supplemental Table 11. Concordance between TumorNext and HapMap NA07019 Reference** | | | | | | |
| --- | --- | --- | --- | --- | --- | --- |
| **HapMap SNP** | **Gene** | **Variant** | **Hapmap Alleles** | **NA07019 Reference Genotype** | **TumorNext Result** | **Concordant?** |
| rs6685892 | NOTCH2 | c.7341T>A | A/T | AT | TT | N* |
| rs3899528 | NOTCH2 | c.680C>A | G/T | GG | GT | N* |
| rs2298258 | DDR2 | c.1260C>G | C/G | CG | CG | Y |
| rs2746462 | SDHB | c.18C>A | . | TT | TT | Y |
| rs1805415 | PARP1 | c.1056A>G | C/T | CT | CT | Y |
| rs3219489 | MUTYH | c.1014G>C | C/G | CG | CG | Y |
| rs1800861 | RET | c.2307G>T | . | GT | GT | Y |
| rs2071702 | MLL | c.7254C>T | C/T | CT | CT | Y |
| rs7993418 | FLT1 | c.3639C>T | A/G | AA | AA | Y |
| rs206076 | BRCA2 | c.6513G>C | C/G | CC | CC | Y |
| rs169547 | BRCA2 | c.7397T>C | C/T | CC | CC | Y |
| rs1058808 | ERBB2 | c.3508C>G | C/G | CG | CG | Y |
| rs1799949 | BRCA1 | c.2082C>T | A/G | AG | AG | Y |
| rs1042522 | TP53 | c.215C>G | C/G | CG | CG | Y |
| rs1548555 | NOTCH3 | c.5362+3T>C | A/G | GG | GG | Y |
| rs273269 | PIK3R2 | c.1911T>C | C/T | CC | CC | Y |
| rs10250 | MAP2K2 | c.660C>A | G/T | GT | GT | Y |
| rs2070094 | BARD1 | c.1519G>A | C/T | CT | CT | Y |
| rs2070093 | BARD1 | c.1518T>C | A/G | GG | GG | Y |
| rs2229571 | BARD1 | c.1134G>C | C/G | CG | CG | Y |
| rs2070096 | BARD1 | c.1053G>C | C/G | CG | CG | Y |
| rs1048108 | BARD1 | c.70C>T | A/G | AG | AG | Y |
| rs1670283 | ALK | c.4381A>G | C/T | CC | CC | Y |
| rs2293564 | ALK | c.1500A>G | C/T | CC | CC | Y |
| rs2246745 | ALK | c.702T>A | A/T | AT | AT | Y |
| rs1047972 | AURKA | c.169A>G | C/T | CC | CC | Y |
| rs7688609 | FGFR3 | c.1953G>A | A/G | AA | AA | Y |
| rs1870377 | KDR | c.1416A>T | A/T | AT | AT | Y |
| rs67622085 | APC | c.4326T>A | . | TA | TA | Y |
| rs246388 | PDGFRB | c.3252A>G | C/T | CC | CC | Y |
| rs351855 | FGFR4 | c.1162G>A | A/G | AG | AG | Y |
| rs1126417 | SDHA | c.891T>C | . | CC | CC | Y |
| rs619203 | ROS1 | c.6686C>G | C/G | GG | GC | N* |
| rs2243378 | ROS1 | c.303A>T | A/T | AA | AA | Y |
| rs4986934 | ESR1 | c.729T>C | C/T | CC | CC | Y |
| rs423023 | NOTCH4 | c.1044C>G | C/G | CG | CG | Y |
| rs1051130 | CCND3 | c.775T>G | A/C | AC | AC | Y |
| rs345730 | EPHA7 | c.2076G>A | C/T | TT | TT | Y |
| rs849389 | PIK3CG | c.972A>G | A/G | GG | GG | Y |
| rs2230460 | PIK3CG | c.2850C>T | C/T | CT | CT | Y |
| rs2228617 | SMO | c.1164G>C | C/G | CG | CG | Y |
| rs1050171 | EGFR | c.2361G>A | A/G | AG | AG | Y |
| rs4489420 | NOTCH1 | c.312T>C | A/G | GG | GG | Y |
| *Note: For HapMap NA07019, the reference genotypes for rs6685892, rs3899528 and rs619203 were initially discordant, but all were Sanger sequenced and found to be concordant with the NGS calls. | | | | | | |

| **Supplemental Table 12. Concordance between TumorNext and HapMap NA10857 Reference** | | | | | | |
| --- | --- | --- | --- | --- | --- | --- |
| **HapMap SNP** | **Gene** | **Variant** | **Hapmap Alleles** | **NA10857 Reference Genotype** | **TumorNext Result** | **Concordant?** |
| rs6685892 | NOTCH2 | c.7341T>A | A/T | AT | AT | Y |
| rs2746462 | SDHB | c.18C>A | . | TT | TT | Y |
| rs1805415 | PARP1 | c.1056A>G | C/T | CC | CC | Y |
| rs3219489 | MUTYH | c.1014G>C | C/G | CG | CG | Y |
| rs3737139 | JAK1 | c.2097C>G | C/G | CG | CG | Y |
| rs1800861 | RET | c.2307G>T | . | GT | GT | Y |
| rs1800863 | RET | c.2712C>G | C/G | CG | CG | Y |
| rs17537350 | FLT1 | c.1704G>A | C/T | CT | CT | Y |
| rs28897727 | BRCA2 | c.4258G>T | . | GT | GT | Y |
| rs206076 | BRCA2 | c.6513G>C | C/G | CC | CC | Y |
| rs1799955 | BRCA2 | c.7242A>G | A/G | AG | AG | Y |
| rs169547 | BRCA2 | c.7397T>C | C/T | CC | CC | Y |
| rs2231301 | BCL2L2 | c.123G>A | A/G | AG | AG | Y |
| rs1748 | TSC2 | c.5202T>C | C/T | CT | CT | Y |
| rs1051771 | TSC2 | c.5397G>C | C/G | CG | CG | Y |
| rs11649210 | FANCA | c.3807G>C | C/G | CG | CG | Y |
| rs2285892 | NF1 | c.2034G>A | A/G | AG | AG | Y |
| rs56013763 | NF1 | c.7045C>T | . | CT | CT | Y |
| rs9901455 | RAD51D | c.234C>T | . | GA | GA | Y |
| rs1058808 | ERBB2 | c.3508C>G | C/G | GG | GG | Y |
| rs1799949 | BRCA1 | c.2082C>T | A/G | GA | GA | Y |
| rs1548555 | NOTCH3 | c.5362+3T>C | A/G | GG | GG | Y |
| rs273269 | PIK3R2 | c.1911T>C | C/T | CC | CC | Y |
| rs4900 | GNA11 | c.771C>T | C/T | TT | TT | Y |
| rs10250 | MAP2K2 | c.660C>A | G/T | TT | TT | Y |
| rs2070094 | BARD1 | c.1519G>A | C/T | CT | CT | Y |
| rs2070093 | BARD1 | c.1518T>C | A/G | GG | GG | Y |
| rs2229571 | BARD1 | c.1134G>C | C/G | CG | CG | Y |
| rs1048108 | BARD1 | c.70C>T | A/G | AG | AG | Y |
| rs1881421 | ALK | c.4587C>G | C/G | CC | CC | Y |
| rs1670283 | ALK | c.4381A>G | C/T | CC | CC | Y |
| rs2293564 | ALK | c.1500A>G | C/T | CC | CC | Y |
| rs2246745 | ALK | c.702T>A | A/T | AT | AT | Y |
| rs1800937 | MSH6 | c.642C>T | C/T | CT | CT | Y |
| rs1047972 | AURKA | c.169A>G | C/T | CC | CC | Y |
| rs7688609 | FGFR3 | c.1953G>A | A/G | AA | AA | Y |
| rs1870377 | KDR | c.1416A>T | A/T | AA | AA | Y |
| rs246388 | PDGFRB | c.3252A>G | C/T | CT | CT | Y |
| rs2228439 | PDGFRB | c.1149G>C | C/G | CG | CG | Y |
| rs351855 | FGFR4 | c.1162G>A | A/G | AG | AG | Y |
| rs1126417 | SDHA | c.891T>C | . | TC | TC | Y |
| rs2243378 | ROS1 | c.303A>T | A/T | AT | AT | Y |
| rs4986934 | ESR1 | c.729T>C | C/T | CC | CC | Y |
| rs1051130 | CCND3 | c.775T>G | A/C | CC | CC | Y |
| rs345730 | EPHA7 | c.2076G>A | C/T | CT | CT | Y |
| rs35775721 | MET | c.534C>T | . | CT | CT | Y |
| rs33917957 | MET | c.1124A>G | . | AG | AG | Y |
| rs2228617 | SMO | c.1164G>C | C/G | CG | CG | Y |
| rs1050171 | EGFR | c.2361G>A | A/G | AG | AG | Y |
| rs1805319 | PMS2 | c.780C>G | C/G | CC | CC | Y |
| rs357564 | PTCH1 | c.3944C>T | A/G | AG | AG | Y |

| **Supplemental Table 13. OncoScan HotSpot Panel** | | |
| --- | --- | --- |
| Gene | Target Mutation | Percent mutant detectable |
| BRAF | BRAFp.V600Kc.1798_1799GT>AA | 20% |
|  | BRAFp.V600Ec.1799T>A | 20% |
|  | BRAFp.G469Ac.1406G>C | 20% |
|  | BRAFp.G469Ec.1406G>A | 30%* |
| EGFR | EGFRp.G719Cc.2155G>T | 20% |
|  | EGFRp.G719Sc.2155G>A | 30% |
|  | EGFRp.G719Ac.2156G>C | 20% |
|  | EGFRp.E746_A750delc.2235_2249del15 | 20% |
|  | EGFRp.E746_A750delc.2236_2250del15 | 30%* |
|  | EGFRp.E746_T751>Ac.2237_2251del15 | 30%* |
|  | EGFRp.L747_A750>Pc.2239_2248TTAAGAGAAG>C | 30%* |
|  | EGFRp.L747_E749delc.2239_2247del9 | 30%* |
|  | EGFRp.L747_T751delc.2240_2254del15 | 20% |
|  | EGFRp.L747_P753>Sc.2240_2257del18 | 20% |
|  | EGFRp.V769_D770insASVc.2307_2308ins9 | 20% |
|  | EGFRp.D770_N771insSVDc.2311_2312ins9 | 20% |
|  | EGFRp.H773_V774insNPHc.2319_2320ins9 | 20% |
|  | EGFRp.T790Mc.2369C>T | 20% |
|  | EGFRp.L858Rc.2573T>G | 30%* |
|  | EGFRp.L861Qc.2582T>A | 30% |
| IDH1 | IDH1p.R132Hc.395G>A | 30% |
| IDH2 | IDH2p.R140Qc.419G>A | 20% |
|  | IDH2p.R172Kc.515G>A | 20% |
| KRAS | KRASp.A146Pc.436G>C | 20% |
|  | KRASp.Q61Hc.183A>C | 20% |
|  | KRASp.Q61Hc.183A>T | 20% |
|  | KRASp.Q61Kc.180_181TC>AA | 30% |
|  | KRASp.Q61Kc.181C>A | 30% |
|  | KRASp.G13Dc.38G>A | 20% |
|  | KRASp.G12Ac.35G>C | 20% |
|  | KRASp.G12Dc.35G>A | 30% |
|  | KRASp.G12Vc.35G>T | 30%* |
|  | KRASp.G12Cc.34G>T | 20% |
|  | KRASp.G12Sc.34G>A | 30% |
| NRAS | NRASp.Q61Lc.182A>T | 20% |
|  | NRASp.Q61Rc.182A>G | 20% |
|  | NRASp.Q61Kc.181C>A | 30%* |
|  | NRASp.G12Dc.35G>A | 20% |
|  | NRASp.G12Vc.35G>T | 20% |
|  | NRASp.G12Cc.34G>T | 30%* |
|  | NRASp.G12Sc.34G>A | 30%* |
| PIK3CA | PIK3CAp.E542Kc.1624G>A | 20% |
|  | PIK3CAp.E545Kc.1633G>A | 30%* |
|  | PIK3CAp.Q546Kc.1636C>A | 30% |
|  | PIK3CAp.H1047Lc.3140A>T | 30% |
|  | PIK3CAp.H1047Rc.3140A>G | 20% |
| PTEN | PTENp.R130*c.388C>T | 20% |
|  | PTENp.R130Gc.388C>G | 20% |
|  | PTENp.R130fs*4c.389delG | 20% |
|  | PTENp.R130Qc.389G>A | 30% |
|  | PTENp.R159Sc.477G>T | 30% |
|  | PTENp.R233*c.697C>T | 30% |
|  | PTENp.P248fs*5c.741_742insA | 30%* |
|  | PTENp.K267fs*9c.800delA | 20% |
| TP53 | TP53p.R306*c.916C>T | 20% |
|  | TP53p.R282Wc.844C>T | 30%* |
|  | TP53p.R273Hc.818G>A | 20% |
|  | TP53p.R273Lc.818G>T | 20% |
|  | TP53p.R273Cc.817C>T | 30% |
|  | TP53p.R273Sc.817C>A | 30% |
|  | TP53p.R249Sc.747G>T | 20% |
|  | TP53p.R248Lc.743G>T | 20% |
|  | TP53p.R248Qc.743G>A | 20% |
|  | TP53p.R248Wc.742C>T | 30%* |
|  | TP53p.G245Cc.733G>T | 30% |
|  | TP53p.G245Sc.733G>A | 20% |
|  | TP53p.Y220Cc.659A>G | 20% |
|  | TP53p.R213*c.637C>T | 20% |
|  | TP53p.R196*c.586C>T | 20% |
|  | TP53p.H179Rc.536A>G | 20% |
|  | TP53p.C176Fc.527G>T | 20% |
|  | TP53p.R175Hc.524G>A | 20% |
|  | TP53p.Y163Cc | 20% |
|  | TP53p.V157Fc.469G>T | 20% |
| Table provided by Affymetrix  * 30% at slightly lower specificity (95% as compared to 99.9% as determined by spike in experiments) | | |

| **Supplemental Table 14. Concordance between TumorNext and OncoScan hotspot panel** | | |
| --- | --- | --- |
| **Sample** | **Mutation** | **Confirmed on TumorNext** |
| RD_005 | NRAS:p.G12D:c.35G>A | Yes |
|  | PIK3CA:p.E545K:c.1633G>A | Yes |
| RD_006 | KRAS:p.G12D:c.35G>A | Yes |
| RD_009 | TP53:p.R282W:c.844C>T | Yes |
| 10-SU4610-B2 | KRAS:p.G12C:c.34G>T | Yes |
| 1008712-139858 | PIK3CA:p.E542K:c.1624G>A | Yes |
| 1009558 | TP53:p.R306*:c.916C>T | Yes |
| 1009643-249766 | TP53:p.R213*:c.637C>T | Yes |
| 1009953-256930 | TP53:p.R175H:c.524G>A | Yes |
| 1011952-163362 | TP53:p.R248Q/L:c.743G>A/T | Yes |
| 1012372-348183 | KRAS:p.G13D:c.38G>A | Yes |
| 1012408-346144 | PIK3CA:p.E545K:c.1633G>A | Yes |
| 1014032-395561 | TP53:p.R175H:c.524G>A | Yes |
| 1014577-406010 | KRAS:p.G12D/V:c.35G>A/T | Yes |
| 3000629-336003 | PIK3CA:p.E545K:c.1633G>A | Yes |
| 3001006-273598 | EGFR:p.E746_A750del:c.2236_2250del15 | Yes |
| BR14-194 | KRAS:p.G12D:c.35G>A | Yes |
|  | TP53:p.R213*:c.637C>T | Yes |
| BR14-239 | KRAS:p.G12V:c.35G>T | Yes |
| BR13-29 | KRAS:p.Q61K:c.180_181TC>TA | Yes |
|  | TP53:p.R273C:c.817C>T | Yes |
| BR14-67 | TP53:p.R248Q:c.743G>A | Yes |
| BR14-88 | NRAS:p.Q61R:c.182A>G | Yes |
| BR14-194 | KRAS:p.G12D:c.35G>A | Yes |
|  | TP53:p.R213*:c.637C>T | Yes |
| BR14-239 | KRAS:p.G12V:c.35G>T | Yes |
| BR13-10 | TP53:p.R248Q:c.743G>A | Yes |
| BR13-139 | TP53:p.R248Q:c.743G>A | Yes |
| BR14-69 | PIK3CA:p.H1047R:c.3140A>G | Yes |
| BR14-83 | PIK3CA:p.H1047R:c.3140A>G | Yes |
|  | TP53:p.R248Q:c.743G>A | Yes |
| BR13-180 | PIK3CA:p.H1047R:c.3140A>G | Yes |
|  | KRAS:p.G12D:c.35G>A | Yes |
| BR14-75 | EGFR:p.L858R:c.2573T>G | Yes |
| BR11-4 | KRAS:p.G12V:c.35G>T | Yes |
| BR13-25 | KRAS:p.G12D:c.35G>A | Yes |
|  | TP53:p.R273H:c.818G>A | Yes |
| BR13-114 | KRAS:p.Q61H:c.183A>C | Yes |
| BR13-184 | KRAS:p.G13D:c.38G>A | Yes |
| BR11-49 | BRAF:p.V600E:c.1799T>A | Yes |
| BR11-50 | BRAF:p.V600E:c.1799T>A | Yes |
| BR12-30 | BRAF:p.V600E:c.1799T>A | Yes |
| BR12-110 | PTEN:p.K267fs*9:c.800delA | Yes |
|  | KRAS:p.G12D:c.35G>A | Yes |
| BR13-162 | TP53:p.R273C:c.817C>T | Yes |
| BR13-163 | KRAS:p.G12V:c.35G>T | Yes |
|  | TP53:p.R273H:c.818G>A | Yes |
| BR14-209 | PIK3CA:p.H1047R:c.3140A>G | Yes |
|  | KRAS:p.G12S:c.34G>A | Yes |
| BR12-15 | KRAS: p.G12D: c.35G>A | Yes |

| **Supplemental Table 15. Concordance between TumorNext and CytoSNP850K** | | | | | | |
| --- | --- | --- | --- | --- | --- | --- |
| **Sample** | **Total heterozygous calls** | **Concordant calls** | **Disconcordant calls** | **Genes with discordant calls*** | **Discordant call Sanger confirmed?** | **Concordance (%)** |
| 1005934 | 49 | 45 | 4 | DNMT3A  ERBB2  IGF1R(2) | No | 100 |
| 1006138_169167 | 57 | 55 | 2 | IGF1R S606 IGF1R S24 | No | 100 |
| 1006807_182985 | 61 | 58 | 3 | DNMT3A  IGF1R S609 IGF1R S24 | No | 100 |
| 1006866_189171 | 58 | 55 | 3 | DNMT3A  IGF1R(2) | No | 100 |
| 1009698_250607 | 63 | 55 | 8 | CDKN2A  DNMT3A  FLT4  IGF1R  MUTYH  MYCN  NOTCH3  RET | No | 100 |
| 1009896_256371 | 56 | 54 | 2 | DNMT3A  IGF1R | No | 100 |
| 394820_3000641 | 46 | 44 | 2 | DNMT3A  IGF1R | No | 100 |
| 384350_3000642 | 66 | 59 | 7 | DNMT3A  EPHA2  FANCA  FLT4  IGF1R(2)  NOTCH4 | No | 100 |
| 383734_3000643 | 53 | 45 | 8 | DNMT3A  FANCA  FLT4  IGF1R  MUTYH  MYCN  NOTCH3  RET | No | 100 |
| 1009558 | 64 | 58 | 6 | IGF1R(2)  MUTYH  NOTCH4  NTRK1  RET | No | 100 |
| 251424_1009749 | 50 | 48 | 2 | DNMT3A  IGF1R(2) | No | 100 |
| 256930_1009953 | 66 | 60 | 6 | DNMT3A  EPHA5  FANCA  IGF1R  MYCN  RET | No | 100 |
| 292837_1011150 | 63 | 56 | 7 | CDKN2A  DNMT3A  FANCA  IGF1R(2)  MYCN  NOTCH3  RET | No | 100 |
| 384009_1013490 | 65 | 64 | 1 | DNMT3A | No | 100 |
| 402958_1014378 | 61 | 57 | 4 | DNMT3A  IGF1R(2)  MYCN | No | 100 |
| RD_006 | 53 | 51 | 2 | DNMT3A  IGF1R | No | 100 |
| RD_008 | 44 | 42 | 2 | DNMT3A  IGF1R | No | 100 |
| RD_011F1 | 43 | 42 | 1 | DNMT3A | No | 100 |
| BR13_29_03_01 | 42 | 38 | 4 | DNMT3A  IGF1R(3) | No | 100 |
| BR_14_4305_01 | 57 | 55 | 2 | DNMT3A  IGF1R | No | 100 |
| BR_14_19405_01 | 66 | 63 | 3 | DNMT3A  FANCD2  IGF1R | No | 100 |
| 334325_1012045 | 47 | 45 | 2 | DNMT3A  IGF1R | No | 100 |
| 1002837_84444 | 52 | 50 | 2 | DNMT3A  IGF1R | No | 100 |
| 1014577 | 63 | 63 | 3 | DNMT3A  FANCA  IGF1R | No | 100 |
| RD_005A1 | 60 | 57 | 3 | DNMT3A  IGF1R(2) | No | 100 |
| 1012374_346270 | 57 | 53 | 4 | DNMT3A  EPHA2  IGF1R(2) | No | 100 |
| 1008712 | 67 | 64 | 3 | DNMT3A  IGF1R(2) | No | 100 |
| 1010098 | 66 | 64 | 2 | DNMT3A  IGF1R | No | 100 |
| 1011142 | 53 | 51 | 2 | DNMT3A  IGF1R | No | 100 |
| 1013301_375808 | 51 | 49 | 2 | DNMT3A  IGF1R | No | 100 |
| 1014032 | 37 | 35 | 2 | DNMT3A  IGF1R | No | 100 |
| S002_53032B_109436 | 64 | 52 | 12 | CDKN2A  DNMT3A  EPHA2  FANCA  IDH2  IGF1R(2)  MUTYH  MYCN  NOTCH4(2)  RET | No | 100 |
| S005_10601C_154417 | 53 | 50 | 3 | DNMT3A  IGF1R  NOTCH4 | No | 100 |
| S09_259 | 68 | 66 | 2 | DNMT3A  IGF1R | No | 100 |
| SP11_180A14 | 59 | 56 | 3 | DNMT3A  IGF1R  EPHA2 | Yes – EPHA2 SNP confirmed | 98 |
| **Total** | **1,980** | **1,845** | **135** |  |  | **99.9** |
| **Concordance = Number of common calls/ total calls x 100** | | | | |  |  |
| *See supplemental table 16 for discordant SNPs  Note: A number in parentheses next to a gene symbol indicates multiple discordant SNPs in that gene | | | | | | |

| **Supplemental Table 16. Discordant CytoSNP850K Array SNPs** | | | | | |
| --- | --- | --- | --- | --- | --- |
| **Gene** | **Refseq** | **c_dot** | **p_dot** | Sanger Confirmed? | NGS Call Correct? |
| CDKN2A | NM_000077 | c.379G>T | p.A127S | No | Yes |
| DNMT3A | NM_175629 | c.89A>C | p.E30A | No | Yes |
| EPHA2 | NM_004431 | c.987C>T | p.P329P | No | Yes |
| EPHA5 | NM_004439 | c.2895G>A | p.G965G | No | Yes |
| ERBB2 | NM_004448 | c.2570A>G | p.N857S | No | Yes |
| FANCA | NM_000135 | c.4036G>A | p.A1346T | No | Yes |
| FANCD2 | NM_033084 | c.1156T>G | p.F386V | No | Yes |
| FLT4 | NM_002020 | c.2196G>A | p.K732K | No | Yes |
| IDH2 | NM_002168 | c.207+1G>C |  | No | Yes |
| IGF1R | NM_000875 | c.15C>T | p.S5S | No | Yes |
| IGF1R | NM_000875 | c.72G>C | p.S24S | No | Yes |
| IGF1R | NM_000875 | c.1825T>C | p.S609P | No | Yes |
| MUTYH | NM_001128425 | c.1187G>A | p.G396D | No | Yes |
| MYCN | NM_005378 | c.207G>A | p.E69E | No | Yes |
| NOTCH1 | NM_017617 | c.312T>C | p.N104N | No | Yes |
| NOTCH3 | NM_000435 | c.3547G>A | p.V1183M | No | Yes |
| NOTCH4 | NM_004557 | c.5614G>A | p.G1872R | No | Yes |
| NOTCH4 | NM_004557 | c.2281G>T | p.G761W | No | Yes |
| NTRK1 | NM_001012331 | c.865C>A | p.Q289K | No | Yes |
| RET | NM_020975 | c.2071G>A | p.G691S | No | Yes |

| **Supplemental Table 17. Genes that are actionable based on CNV status** | | | | | | | | | |
| --- | --- | --- | --- | --- | --- | --- | --- | --- | --- |
| ABL1 |  | CCNE1 | CTNNB1 | EWSR1 | IDH1 | MAP2K1 | MTOR | PIK3R1 | RUNX1 |
| AKT1 | BAP1 | CDH1 | DDR2 | FBXW7 | IDH2 | MAP2k4 | MYC | PML | SMAD4 |
| AKT2 | BCL2L11 | CDK4 | DNMT3A | FGF4 | IGF1R | MCL1 | NF1 | PTEN | SMARCA4 |
| ALK | BCR | CDK6 | E2F3 | FGFR1 | JAK2 | MDM2 | NF2 | PTPRD | SOX2 |
| APC | BRAF | CDK8 | EGFR | FGFR2 | KDM6A | MDM4 | NKX2-1 | RARA | STK11 |
| AR | BRCA1 | CDKN1A | EML4 | FGFR3 | KDR | MET | NOTCH1 | RB1 | TET2 |
| ARID1A | BRCA2 | CDKN1B | EPHB2 | FLT3 | KIF5B | MGMT |  | RET | TP53 |
| ASXL1 | CCND1 | CDKN2A | ERBB2 | FRS2 | KIT | MLL | NRAS | RICTOR | TSC1 |
| ATM | CCND2 | CDKN2B | ERBB3 | HIF1A | KRAS | MPL | PDGFRA | ROS1 | TSC2 |
| AURKA | CCND3 | CEBPA | ESR1 | HRAS | LRP1B | MSH6 | PIK3CA | RPTOR | VHL |

| **Supplemental Table 18. Examples of discordant calls between OncoScan and CytoSNP850K** | | | |
| --- | --- | --- | --- |
| CytoSNP850K Call | OncoScan Call | BAF/logR Review | Notes |
| CN Gain | CN Loss | CN Loss | 22 calls limited to 5 samples – these 5 samples showed identical BAF and LogR plots for all calls on both platforms. |
| CN Gain | LOH | LOH | 20 calls - all calls determined to be LOH |
| LOH | No Event | No Event | 18 calls – CytoSNP850K listed genes on the X chromosome in males as LOH and OncoScan did not list a CNV event. One LOH event on Chromosome 2 was not reported by OncoScan. |
| CN Gain | No Event | CN Gain and No Event | 2 calls – logR and BAF plots matched for both calls. OncoScan did not call a CN Gain for 1 sample and CytoSNP incorrectly called a CN Gain for the other sample. |
| LOH | CN Gain | CN Gain | 5 calls – all calls determined to be CN Gain |
| LOH | CN Loss | CN Loss | 1 call |
